# Evaluation of methods for characterizing the fine particulate matter emissions from aircraft and other diffusion flame combustion aerosol sources

**DOI:** 10.1016/j.jaerosci.2024.106352

**Published:** 2024-05

**Authors:** Robert Giannelli, Jeffrey Stevens, John S. Kinsey, David Kittelson, Alla Zelenyuk, Robert Howard, Mary Forde, Brandon Hoffman, Cullen Leggett, Bruce Maeroff, Nick Bies, Jacob Swanson, Kaitlyn Suski, Gregory Payne, Julien Manin, Richard Frazee, Timothy B. Onasch, Andrew Freedman, Imad Khalek, Huzeifa Badshah, Daniel Preece, Vinay Premnath, Scott Agnew

**Affiliations:** a U. S. Environmental Protection Agency, Office of Transportation and Air Quality, National Vehicle and Fuels Emissions Laboratory, Ann Arbor, MI, 48105, USA; b Shu Research LLC, Mebane, NC 27302, Formerly U. S. Environmental Protection Agency, Office of Research and Development, Research Triangle Park, NC, 27711, USA; c University of Minnesota, Department of Mechanical Engineering, Minneapolis, MN, 55455, USA; d U.S. Department of Energy, Pacific Northwest National Laboratory (PNNL), Richland, WA, 99352, USA; e Arnold Engineering Development Complex, Arnold Air Force Base, TN, 37389, USA; f U.S. Air Force, Wright Patterson Air Force Base, OH (Formerly Arnold Engineering Development Complex, Arnold Air Force Base, TN, 37389, USA; g Minnesota State University, Bloomington, MN, 55431, USA; h Artium Technologies Inc., Sunnyvale, CA, 94085, USA; i Singularity Scientific Consulting Services, LLC, Whitmore Lake, MI, 48189, USA; j Aerodyne Research Inc., Billerica, MA, 01821, USA; k Southwest Research Institute, San Antonio, TX, 78238, USA

**Keywords:** Combustion aerosols, Aircraft turbine engines, Black carbon, Elemental carbon, Laboratory generated soot

## Abstract

The U. S. Environmental Protection Agency in collaboration with the U. S. Air Force Arnold Engineering Development Complex conducted the VAriable Response In Aircraft nvPM Testing (VARIAnT) 3 and 4 test campaigns to compare nonvolatile particulate matter (nvPM) emissions measurements from a variety of diffusion flame combustion aerosol sources (DFCASs), including a Cummins diesel engine, a diesel powered generator, two gas turbine start carts, a J85-GE-5 turbojet engine burning multiple fuels, and a Mini-CAST soot generator. The VARIAnT research program was devised to understand reported variability in the ARP6320A sampling system nvPM measurements. The VARIAnT research program has conducted four test campaigns to date with the VARIAnT 3 and 4 campaigns devoted to: (1) assessing the response of three different black carbon mass analyzers to particles of different size, morphology, and chemical composition; (2) characterizing the particles generated by 6 different combustion sources according to morphology, effective density, and chemical composition; and (3) assessing any significant difference between black carbon as determined by the 3 mass analyzers and the total PM determined via other techniques. Results from VARIAnT 3 and 4 campaigns revealed agreement of about 20% between the Micro-Soot Sensor, the Cavity Attenuated Phase Shift (CAPS PM_SSA_) monitor and the thermal-optical reference method for elemental carbon (EC) mass, independent of the calibration source used. For the LII-300, the measured mass concentrations in VARIAnT 3 fall within 18% and in VARIAnT 4 fall within 27% of the reference EC mass concentration when calibrated on a combustor rig in VARIAnT 3 and on an LGT-60 start cart in VARIAnT 4, respectively. It was also found that the three mass instrument types (MSS, CAPS PM_SSA_, and LII-300) can exhibit different BC to reference EC ratios depending on the emission source that appear to correlate to particle geometric mean mobility diameter, morphology, or some other parameter associated with particle geometric mean diameter (GMD) with the LII-300 showing a slightly stronger apparent trend with GMD. Systematic differences in LII-300 measured mass concentrations have been reduced by calibrating with a turbine combustion as a particle source (combustor or turbine engine). With respect to the particle size measurements, the sizing instruments (TSI SMPS, TSI EEPS, and Cambustion DMS 500) were found to be in general agreement in terms of size distributions and concentrations with some exceptions. Gravimetric measurements of the total aerosol mass produced by the various DFCAs differed from the reference EC, BC and integrated particle size distribution measured aerosol masses. The measurements of particle size distributions and single particle analysis performed using the miniSPLAT indicated the presence of larger particles (≳150 nm) having more compact morphologies, higher effective density, and a composition dominated by OC and containing ash. This increased large particle fraction is also associated with higher values of single scattering albedo measured by the CAPS PM_SSA_ instrument and higher OC measurements. These measurements indicate gas turbine engine emissions can be a more heterogeneous mix of particle types beyond the original E–31 assumption that engine exit exhaust particles are mainly composed of black carbon.

## 1. Introduction

Although the aviation sector currently contributes about 3% to the U.S. inventory of regulated pollutant emissions (U.S. Environmental Protection Agency, 2023), the commercial aviation industry has grown substantially in the United States and worldwide. As reported in a 2009 U. S. Government Accountability Office (GAO) report (United States Government Accountability Office, 2009), from 1981 through 2008, airline passenger traffic increased 226 % in the United States on a revenue passenger mile basis and 257% globally on a revenue passenger kilometer basis. During the period from 2010 to 2019 the growth in demand of over 5% annually was greater than aircraft fuel efficiency gains of 1.8% annually during the same period. CO_2_ emissions are estimated to more than double by 2050 (IEA, 2023; McKinseyCompany, 2020). At this point, most of the efforts for air transport to reduce net CO_2_ and other combustion emissions has been to substitute petroleum based Jet-A aviation fuel with sustainable aviation fuels (SAFs) (IATA, n. d.; U.S. DOE, n. d. a; U.S. DOE, n. d. b; ICAO, n. d.; EASA, n. d.).

As a consequence, particulate matter emissions from aircraft engine exhaust will continue to increase. Since particulate matter (PM) has well known public health consequences and combustion black carbon aerosols contribute to climate change (IEA, 2022; Lee et al., 2021; Stjern et al., 2017), aircraft emissions as a source will continue to be a concern for public health and climate forcing.

Until recently, PM generated by commercial aircraft engines has been regulated through a visibility standard (i.e., smoke number) rather than a direct measure of PM mass and number emissions (ICAO, 2021a; U.S. EPA, n. d.). Although total PM emissions consist of volatile, semi-volatile, and non-volatile PM (nvPM), currently, the International Civil Aviation Organization (ICAO) only sets a limit on the emissions of nvPM number and mass from commercial engines as determined over a regulated landing and take-off cycle (LTO) consisting of take-off, climb out, approach, and idle power (ICAO, 2021b; SAE International, 2013; SAE International, 2019; SAE International, 2021) and measured at, or near, the exit of the engine. Recent surveys of the nvPM emissions from the U. S. and European regulated fleet over the LTO have indicated a range of nvPM mass emissions from 0.80 to 267 mg/kg fuel with the range of nvPM number emissions being 7.6 × 10^12^ to 5.8 × 10^15^ particles/kg fuel (ICAO, 2021b).

At the request of the ICAO, the SAE International’s E–31 Aircraft Engine Gas and Particulate Emissions Measurement Committee developed Aerospace Recommended Practice (ARP) 6320A for the measurement of nvPM mass and number concentrations from gas turbine engines (SAE International, 2021). In ARP6320A, nvPM is defined as those particles that exist in the exhaust at a temperature of 350 °C with nvPM mass measured as black carbon determined by optical absorption methods based on studies indicating that nvPM is mainly (90% or more) composed of refractory carbonaceous particles (Petzold et al., 1999; SAE International, 2012) and trace amounts (10% or less) of other materials. The sampling and measurement system used is about 35 m long, temperature controlled, dilutes the sample at a location about 8 m from the tip of the sample probe, and splits the flow for separate measurements of gaseous constituents and nvPM mass and number emissions (SAE International, 2021). For nvPM mass emissions, two optical-based instruments currently specified in ARP6320A for the determination of black carbon (BC) are the Artium Technologies LII-300 (Bachalo et al., 2002) and the AVL Model 483 Micro Soot Sensor, (Schindler et al., 2004). A third optical-based instrument, the Aerodyne CAPS PM_SSA_ (Onasch et al., 2015), has also been evaluated as part of the program described here. For nvPM particle number concentration measurements, ARP6320A specifies an additional serial diluter, volatile particle remover, and condensation particle counter (CPC) with a 10 nm cut-point.

Extensive research has been conducted on PM emissions from aircraft gas turbines (e.g., Corbin, Schripp, et al., 2022; Drozd et al., 2012; Elser et al., 2019; Kinsey et al., 2011; Timko, Onasch, et al., 2010). The majority of these studies have focused on characterizing the source with relatively few dedicated to assessing the response of the instrumentation (Kinsey, Squier, Timko, Dong, & Logan, 2019). A number of test campaigns (Kinsey et al., 2021; Kittelson et al., 2022; Lobo et al., 2015, 2016, 2020) have also been conducted to study the performance of the ARP6320A sampling system including the VAriable Response In Aircraft nvPM Testing (VARIAnT) 1 and 2 research programs conducted in 2014 and 2015 (Kinsey et al., 2021; Kittelson et al., 2022). This program was sponsored by the U. S. Environmental Protection Agency’s National Vehicle and Fuel Emissions Laboratory (NVFEL) in Ann Arbor, MI in collaboration with the U. S. Air Force Arnold Engineering Development Center (AEDC) in Tullahoma, TN, involving the comparison of two ARP6320A compliant sampling systems while concurrently sampling the emissions from a J85-GE-5 turbojet engine. As follow on to this work, in January and March 2017 and August 2018, EPA conducted the VARIAnT 3 and 4 test campaigns. The objectives of these latter two test campaigns were to:

1. Assess the response of the three mass analyzer types to particles of different size, morphology, and chemical composition as generated by 6 different DFCASs after performing the ARP6320A prescribed mass instrument calibrations on the three different types of mass analyzers using a common DFCAS. Included was an evaluation of the exclusive use of a turbine DFCAS for calibration of the LII-300 as compared to earlier VARIAnT campaign results.
2. Characterize the particles generated by each DFCAS according to morphology by transmission electron microscopy (TEM), effective density versus particle size using a Cambustion Centrifugal Particle Mass Analyzer/Differential Mobility Analyzer (DMA/CPMA/CPC)^1^ system, and relating particle size, morphology, chemical composition and mass using the single particle mass spectrometer (or miniSPLAT; Zelenyuk et al., 2015).
3. Assess any significant differences between black carbon as measured by the three mass analyzer types to the total PM determined via other techniques [i.e., gravimetric analysis of Teflon filters and integrated particle size distribution (IPSD) (Liu et al., 2009; Maricq, & Xu, 2004; Park,Cao, Kittelson, & McMurry, 2003)]. In addition, an assessment was made of the larger particle sizes (particles with diameters ≳ 150 nm) found to be present in the engine exhaust, but not previously characterized, and their impact on the calculated mass emissions.

This paper summarizes the results of the VARIAnT 3 and 4 test campaigns with future papers detailing the determinations of effective density and chemical composition as a function of particle size. The TEM analyses conducted on samples collected in the two campaigns have been described previously by Singh (2019), Singh and Vander Wal (2019) and Kumal et al. (2020). Researchers and supporting personnel participating in each campaign are listed in the SI.

## 2. Experimental

### 2.1. Combustion sources and fuels

In the VARIAnT 3 and 4 test campaigns, a total of 40 test series were performed on a variety of DFCASs burning multiple fuel types. In VARIAnT 3, exhaust emissions from a Cummins Model ISX15 heavy duty diesel engine, Libby Welding Model GT-05 and LGT-60 turbine powered start carts, a General Electric J85-GE-5 turbojet engine, and an ISUZU 4LE2T diesel-generator set were evaluated using standard and alternative fuels (see Table 1). During VARIAnT 4, the different fuels used in the Model LGT-60 start cart and J85-GE-5 aircraft gas turbine were similar to those used in VARIAnT 3. Additionally, Jing Model 5201 and 6200 miniCAST (Combustion Aerosol STandard) propane laboratory burners were evaluated. Except for the Cummins diesel engine, all measurements were conducted at the University of Tennessee Space Institute (UTSI) Propulsion Research Facility. The Cummins engine measurements were performed at NVFEL. A summary of each test series conducted during the two campaigns is provided in Tables S-1 of the SI along with a description of each DFCAS and its associated operating characteristics in Tables S-2 through S-6.

Note that there were day-to-day variations in the DFCAS combustion conditions for the same nominal set points which introduced scatter in the data not attributable to measurement error. This is discussed below and in Section 7.5 of the SI.

With respect to fuel types, Table 1 provides a summary of the various DFCASs, fuels, and number of test series conducted in each campaign. The certification diesel fuel used in the Cummins engine met the general specifications outlined in 40 U. S. Code of Federal Regulations, Part 1065 (U. S. Environmental Protection Agency (EPA a)). The HDRD fuel is a biofuel made from hydrodeoxygenation and catalytic hydroisomerization of animal fats and vegetable oils followed by distillate fractionation. It consists mostly of branched and linear paraffins having carbon numbers ranging from C9 to C18. The Camelina fuel was manufactured by catalytic hydroprocessing of extracts from the *camelina sativa* plant to produce a sustainable aviation fuel (SAF) consisting of primarily *n*-paraffins and iso-paraffins.

The composition of the Jet-A and Camelina/Jet-A blends were determined on-site by the AEDC laboratories using applicable ASTM methods. The composition of the certification diesel fuel was determined by NVFEL. Table 2 is a summary of the analyses of each fuel tested in the program.

### 2.2. Equipment layout and instrumentation

**Fig. 1.**
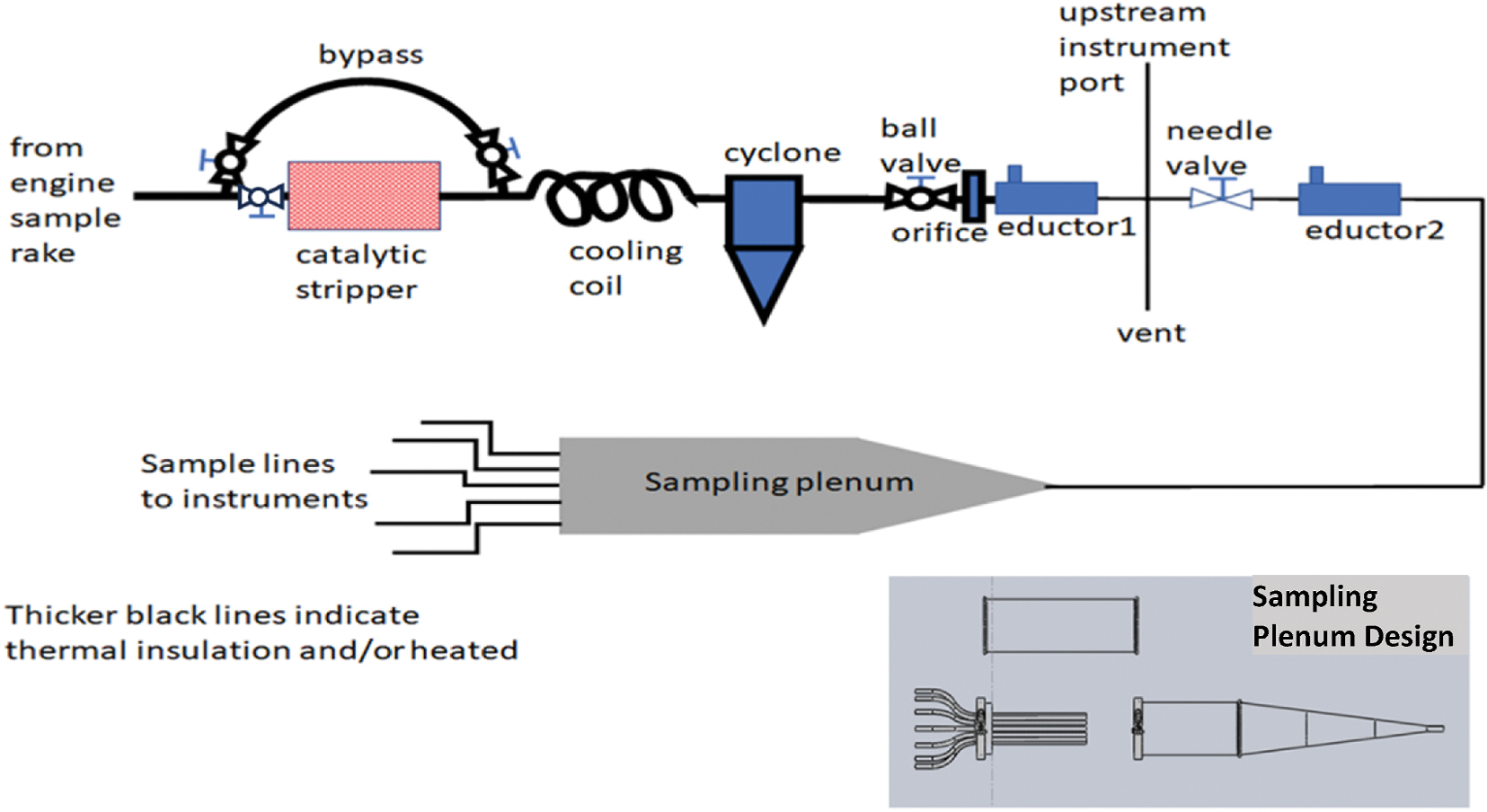
Diagram of the general sampling and analysis apparatus used in VARIAnT 3 and 4.

Unlike VARIAnT 1 and 2 which used AIR6241 (SAE International, 2013) compliant sampling systems,^2^ a custom-designed sampling system was used in VARIAnT 3 and 4 campaigns to collect, condition, and deliver the exhaust sample to an extensive suite of analytical instruments which was not possible with the compliant system. A general diagram of the system is shown in Fig. 1 with further details provided in the SI (see Sections 3 through 6) for each test campaign.

As shown in Fig. 1, an exhaust gas sample extracted by the probe was sent to a Catalytic Instruments model CS15 catalytic stripper operated at a temperature of 350 °C which could be bypassed as desired. Next, the sample temperature was reduced in a cooling coil and particles with nominal diameters larger than 0.6 μm were removed by a BGI Model Scc 2.354 cyclone separator^3^ with the specific size cutoff depending upon the flow rate through the cyclone. Downstream of the cyclone, the sample was diluted in one or more AirVac Model TD110HSS, TD190HSS, and TD260HSS eductors operated by high pressure (50–60 psi) N_2_ to achieve dilution factors of approximately 3:1 to 150:1, depending on test conditions. Downstream of the eductors, the diluted exhaust flow was directed to a multi-port sampling plenum (inset in Fig. 1) containing a series of individual probes arranged coaxially. The plenum operated with excess flow which was vented through a large dump port located in the center of the probe array. Each individual probe in the plenum was connected to the appropriate analytical instrument, the diameter of which was sized to accommodate the nominal sample flow to the instrument. The sampling plenums used in the two campaigns were of the same basic design as shown in the inset to Fig. 1. Additional information on the sampling plenums and their design is provided in the SI.

A wide variety of instrument types were used to characterize the aerosol produced by each DFCAS tested in the two campaigns. The parameters measured included: total particle mass and number; BC mass; organic and elemental carbon (OC and EC) mass; particle size distribution (PSD) by both number and mass; particle morphology; particle chemical composition (VARIAnT 4), and various gaseous components including CO, CO_2_, SO_2_, NO_x_, and total hydrocarbons. In the case of BC mass and overall particle size distribution, multiple instruments of the same type were operated simultaneously for comparison. Table 3 provides a description of each instrument type broken down by operating principle, campaign, and number of analyzers used in each campaign. As indicated in Table 3, an extensive array of sizing instruments was employed during VARIAnT 4 including Scanning Mobility Particle Sizers (SMPSs), centrifugal mass analyzer (CPMA), and a single particle mass spectrometer.

### 2.3. Sample and data analyses

As specified in ARP6320A, the EC concentration from quartz filter sample collection was used as the mass reference and determined by dividing the net mass collected on the filter by the total volume of sample gas passing through the filter during each test point. The filter sampling configuration for these tests included a primary single quartz filter together and in parallel with a dual filter holder. The dual filter holder consisted of a Teflon filter for removal of total PM in series with a back-up quartz filter used for OC gaseous artifact corrections (Turpin et al., 1994).

Analysis of the reference method, manual, OCEC filters was conducted daily on-site using the thermal-optical transmittance method as originally described by Birch and Cary (1996) and published as ASTM 6877–13 (ASTM International, 2018). Test point averages from the BC mass analyzers used during each campaign were compared to the corresponding reference method, using both manual and semi-continuous, EC mass values for the DFCAS sources sampled. This procedure allowed for a continual evaluation of the mass instrument data collected and allowed for identification of any potential measurement issues.

The net EC mass from thermal-optical analysis of the primary single filter was calculated by subtracting the average EC mass determined from thermal-optical analysis of the blank filters routinely collected during each campaign. The OC mass from the OCEC thermal-optical analysis of the primary filters were corrected for gas phase artefacts according to the method described by Turpin et al. (1994) by subtracting the OC mass determined by thermal-optical analysis of the quartz filter located downstream of the Teflon filter from that determined on the single, primary quartz filter.

### 2.4. Instrument calibration and quality control checks

A series of standard calibrations and quality control checks were performed during both campaigns. Prior to both VARIAnT 3 and 4, the mass instruments were calibrated according to the requirements of ARP6320A and both the manual and semi-continuous OCEC analyzers were calibrated using a series of standard sucrose solutions and method blanks. Quality control checks were also performed on a daily basis which included an evaluation of the sizing instruments using a standard test aerosol and completion of operational checks using standardized procedures for each instrument. Calibrations of the BC analyzers for VARIAnT 3 were conducted at Southwest Research Institute (SwRI) using a combustor sector rig and for VARIAnT 4 at AEDC using a turbine start cart. For VARIAnT 3 the additional MSS plus was calibrated (denoted by “AVL cal.“) at AVL in Graz, Austria. Details of the instrument calibrations and quality checks conducted in VARIAnT 3 and 4 are provided in Section 7 of the SI.

## 3. Experimental results

### 3.1. Mass instrument response by source type

**Fig. 2.**
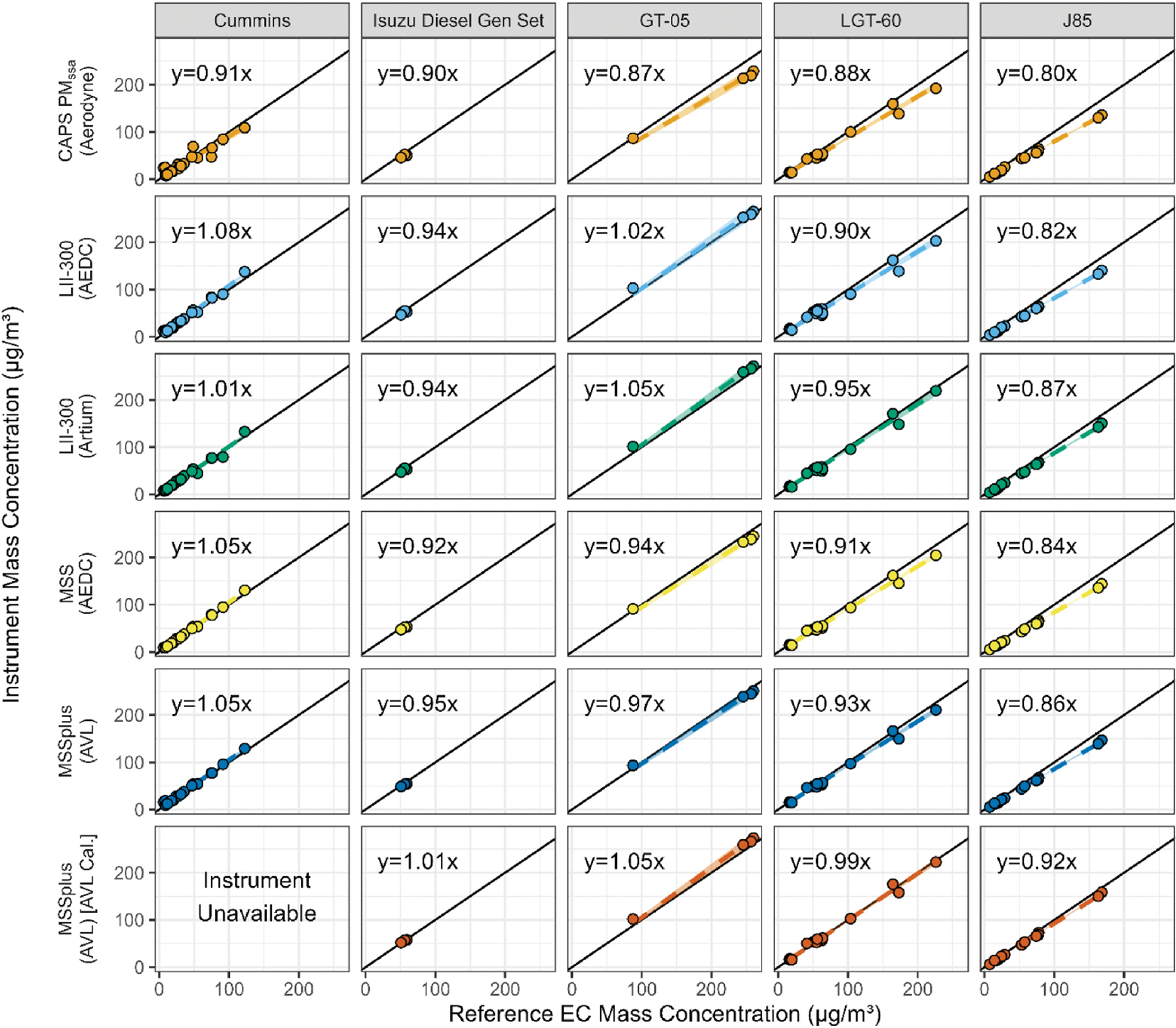
VARIAnT 3 comparison of the different BC instrument mass concentration measurements to the manual EC concentration grouped by DFCAS type for all engine sources and stripper configurations. The instrument owner is listed in parenthesis after each instrument name. Note the MSSplus instrument shown in the bottom row was calibrated using the AVL MiniCAST, instead of the SwRI combustor. In each of the graphs the shaded regions represent a 95% confidence interval for the fit and there is a solid black 1:1 line for reference.

**Fig. 3.**
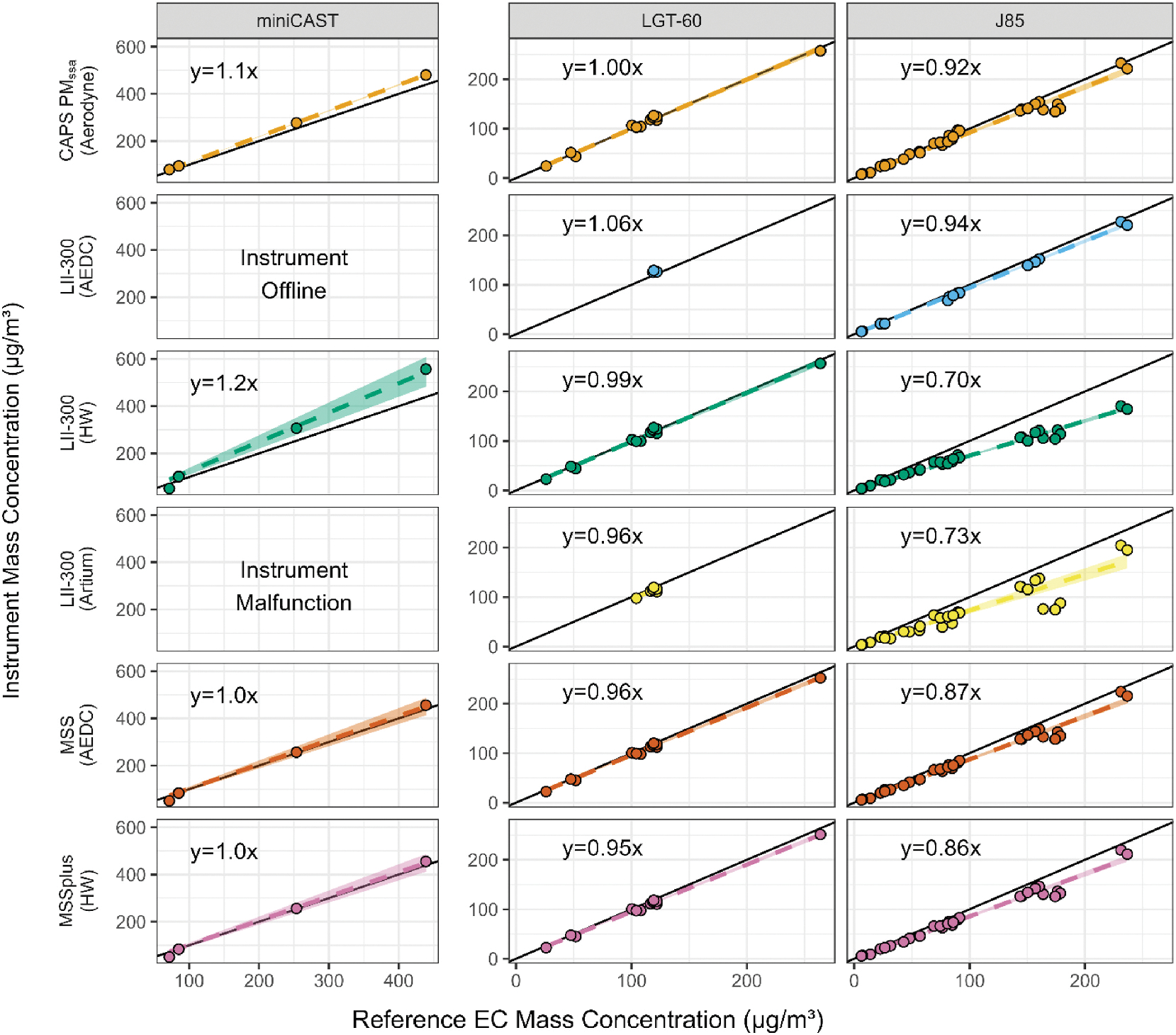
VARIAnT 4 comparison of manual EC to BC instrument mass concentration measurements for the J85, miniCAST, and LGT-60 start cart DFCAS sources with and without use of a catalytic stripper. The instrument owner is listed in parenthesis after each instrument name. Note that LII-300s were owned by AEDC, Honeywell (HW), and a loaner LII-300 provided by Artium Technologies. In each of the graphs the shaded regions represent a 95% confidence interval for the fit and there is a solid black 1:1 line for reference.

For both VARIAnT 3 and 4, a variety of DFCASs were used to compare the response of different real time mass instruments to the elemental carbon (EC) determined using ASTM Method D6877–13 (ASTM International, 2018). In each test campaign these comparison tests were performed after the calibration was conducted and calibration constants (see SI Tables S-8 and S-10) were applied to the instruments. Note that although both the manual and semi-continuous methods for determining EC were used, all data presented here are for the manual method of analysis only. A comparison of the two methods will be included in a subsequent publication. Figs. 2 and 3 show the response of each real time mass instrument to the reference EC method for all sources, fuels, and run conditions during each campaign. Each point on the plots shows the results for a single test point, with the mass concentration determined from the reference EC method on the x-axis and the average mass concentration from the real time instrument on the y-axis. A single row of graphs in Figs. 2 and 3 show the response of an individual real-time mass instrument compared to the EC reference method for each DFCAS tested. Each panel of Figs. 2 and 3 also shows the results of a simple linear fit with no intercept,^4^ the dashed line representing the fit, a shaded region showing a 95% confidence interval for the fit and a solid 1:1 line for reference. Summary statistics for each fit are provided in Tables S-13 and S-14 of the SI, respectively.

For both diesel engine sources tested in VARIAnT 3 (first 2 columns of Fig. 2), all slopes from the fit of the data show agreement with the reference EC at 10% or better. There is better agreement among types of similar instruments that were calibrated at the same calibration laboratory with the same calibration source (i.e., SwRI calibration). In these cases, the LII-300s had slopes that were within 7% of each other on the Cummins diesel engine and both had the same slope for the Isuzu diesel generator set. For the MSSplus and AEDC MSS that were calibrated at SwRI, the agreement between the two instruments was 3% or better. A difference of 9% was observed between fit slopes of the MSSplus calibrated at AVL and the AEDC MSS calibrated at SwRI. Note that the MSSplus calibrated at AVL was not available during the Cummins diesel engine tests at NVFEL. The Isuzu diesel generator was run for a limited number of test points and range of concentrations.

For the two start carts (third and fourth columns of Fig. 2), all slopes from the fit of the data show that each mass instrument agreed with the reference EC method at 13% or better. Between the two LII-300s that were calibrated at SwRI, the slopes from the fit were within 5% of each other. For the two MSSs that were calibrated at SwRI, the slopes from the fit were within 3% of each other. A difference of 8% was observed between fit slopes of the MSSplus calibrated at AVL and the AEDC MSS that was calibrated at SwRI indicating differences between the two laboratory calibrations.

For both start carts, the MSSplus calibrated at AVL had systematically higher response than the MSSplus and MSS calibrated at SwRI; as mentioned above, indicating differences between the two laboratory calibrations.

The J85 data are shown in the last column of Fig. 2. All the slopes for these mass instruments were within 20% of reference EC. Between the two LII-300s that were calibrated at SwRI, the slopes from the fit were within 5% of each other. For the two MSS instruments that were calibrated at SwRI, the slopes from the fit were within 2% of each other. A difference of 8% was found between fit slopes of the MSSplus calibrated at AVL and the AEDC MSS that was calibrated at SwRI. Again, the MSSplus that was calibrated at AVL exhibited systematically higher response than the MSSplus and MSS calibrated at SwRI.

For the VARIAnT 4 J85 data shown in Fig. 3, all real time instruments had systemically lower response relative to reference EC compared to the other sources. All of the slopes for these mass instruments were within 30% of the reference EC method with poor agreement observed between the three fully operational LII-300s. Between the three LII-300s, the slopes from the fit were within 24% whereas the slopes for the two MSS instruments were within 1%. It is also noteworthy that the Honeywell and loaner (Artium) LII-300s showed the poorest performance against EC with slopes of 0.70 and 0.73, respectively.

In the case of the start cart during VARIAnT 4, Fig. 3 shows that all instruments were within 6% of the reference EC method. The best agreement (i.e., slope of 1) was for the CAPS PM_SSA_ followed by the Honeywell LII-300 with a slope of 0.99. For the three LII-300s, the slopes from the fit were within 10% of each other whereas the slopes from the two MSSs were again within 1%.

Finally, for the miniCAST in Fig. 3, except for the loaner (Artium) LII-300, all instruments were within 24% of the reference EC method. The loaner (Artium) LII-300 was apparently not operating properly during the miniCAST tests. For the two MSSs, the slopes from the fit were within 1% of each other. Note the limited data for the CAST measurements, however.

### 3.2. Mass instrument response by electrical mobility particle size

**Fig. 4.**
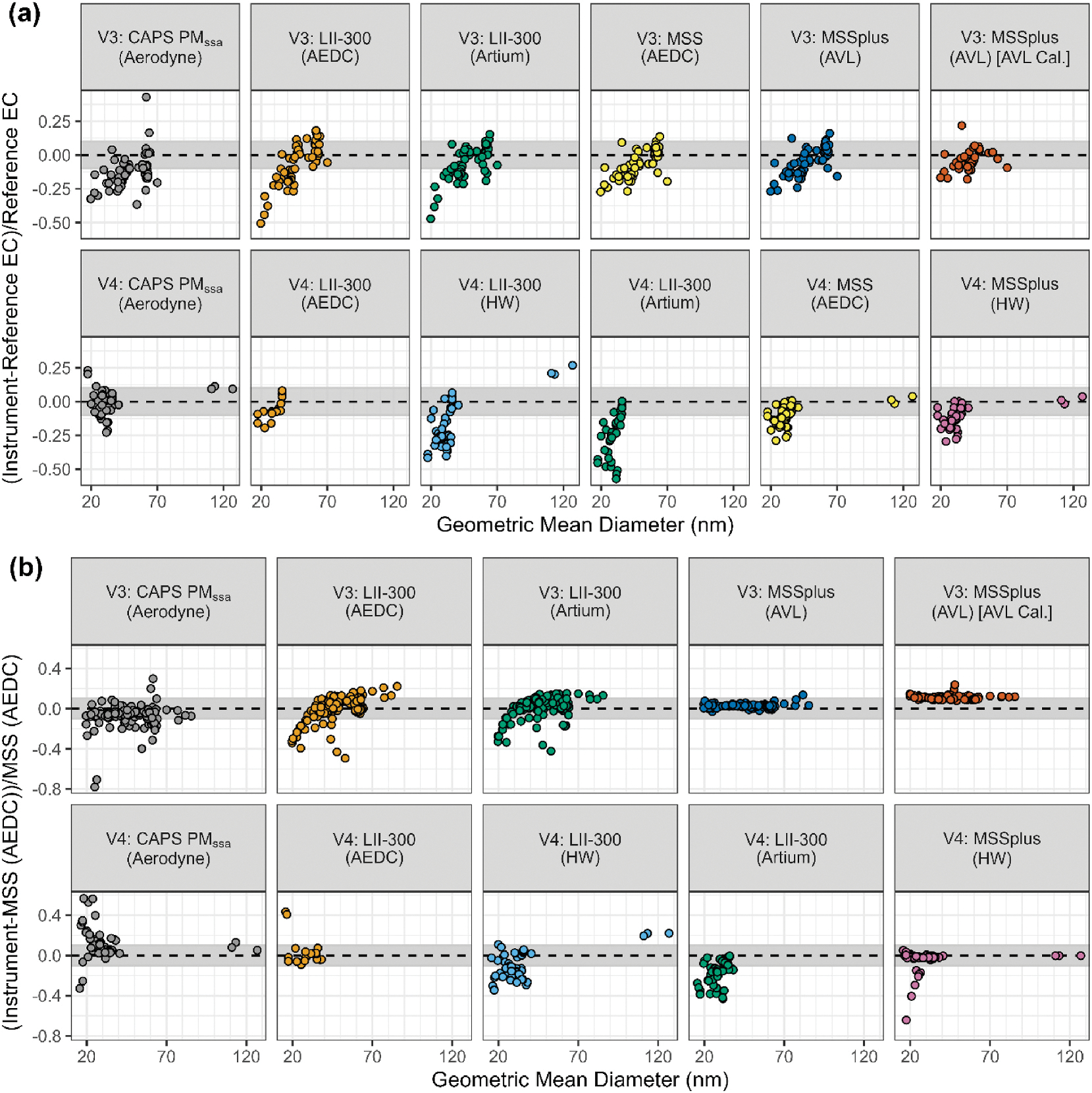
The relative response of the various mass analyzers to measured geometric mean particle diameter to: (a) manually determined reference EC when filters were collected; and (b) BC from the AEDC MSS for all engine sources where data are available. The grey shaded area indicates the range from −0.1 to 0.1. Note that the MSSplus in VARIAnT 3 had a different calibration at AVL from the other instruments. Also, the CAPS PM_SSA_ used in the two campaigns operated at different wavelengths as indicated in Table 1. The instrument owner is listed in parenthesis after each instrument name.

The particle size distribution of the aerosol generated by each DFCAS was determined by a variety of different sizing instruments depending on test campaign. Using the data generated by the NVFEL 3936 SMPS for VARIAnT 3 and the NVFEL 3938 SMPS in VARIAnT 4, the sensitivity of the mass analyzers to particle size was assessed as shown in Fig. 4 for all fuel types and engine sources during VARIAnT 3 and 4. In Fig. 4a, the relative response of each mass instrument as referenced to EC versus the calculated geometric mean particle diameters are graphed. Fig. 4b shows a similar plot, but the mass instrument response is referenced to BC from the AEDC MSS. Although the MSS is not a true standard, it was selected for this comparison since it exhibited the least variability with respect to particle geometric mean diameter and more MSS data are available as compared to EC.

Although physical processes associated with light scattering from particulate matter such as soot internal structure and/or absorptive properties are not necessarily directly associated with particle geometric mean diameters, as shown in Fig. 4a, all of the mass instrument responses tended to deviate from EC with decreasing particle size. These data, however, only illustrate the trend with geometric mean diameter and are not meant to make a statement about the physics behind this trend. There are other works [Michelson (2017); Van de Hulst (1981)] containing more detail on the possible physical mechanisms. The recent work by Vander Wal et al. (2023) has a subset of the VARIAnT 3 data (2 diesel and 2 J85 points) presented here and does not address the difference at the lowest diameter.

The CAPS PM_SSA_ and MSS instruments generally show less sensitivity to particle size below ~35 nm than the LII-300. Except for the AEDC LII-300, instrument responses relative to the EC reference were significantly lower for smaller particles independent of mass concentrations (concentrations were varied by dilution for each engine operation point). Deviations were as high as ~50%. The apparent association with particle geometric mean diameter may be due to particle optical properties or other factors, e.g., Durdina et al. (2016), Michelson (2017). These results are consistent with previous data collected during VARIAnT 1 and 2 as reported by Kinsey et al. (2021). However, an exception to this general trend was found for the AEDC LII-300 in VARIAnT 4, whereby the deviation is similar to that of the MSS instruments and CAPS PM_SSA_. This sensitivity is particularly more noticeable for J85 nvPM emissions since it produces more particles *<*35 nm than the other DFCASs evaluated.

Similar results are shown in Fig. 4b as referenced to MSS BC where more data are available as compared to EC. Except for the VARIAnT 4 AEDC LII-300, the LII-300 instruments appear to deviate from the AEDC MSS at particle geometric mean mobility diameters below ~35 nm similar to their response to EC. A statistically significant difference in response was found for particles smaller than ~35 nm compared to larger particles (see the *t*-test results in the SI Table S-17). The CAPS PM_SSA_ and MSS instruments have a relatively flat response relative to the AEDC MSS with respect to particle geometric mean mobility diameter and the CAPS PM_SSA_ data show more scatter compared to the MSS instruments. Also note that the MSSplus with the AVL calibration had a constant offset from the AEDC MSS in VARIAnT 3 probably related to the fact it was calibrated at AVL using a different calibration source and laboratory whereas all of the other VARIAnT 3 BCE instruments were calibrated at SwRI using a turbine engine combustor sector.

### 3.3. Sizing instrument comparison

**Fig. 5.**
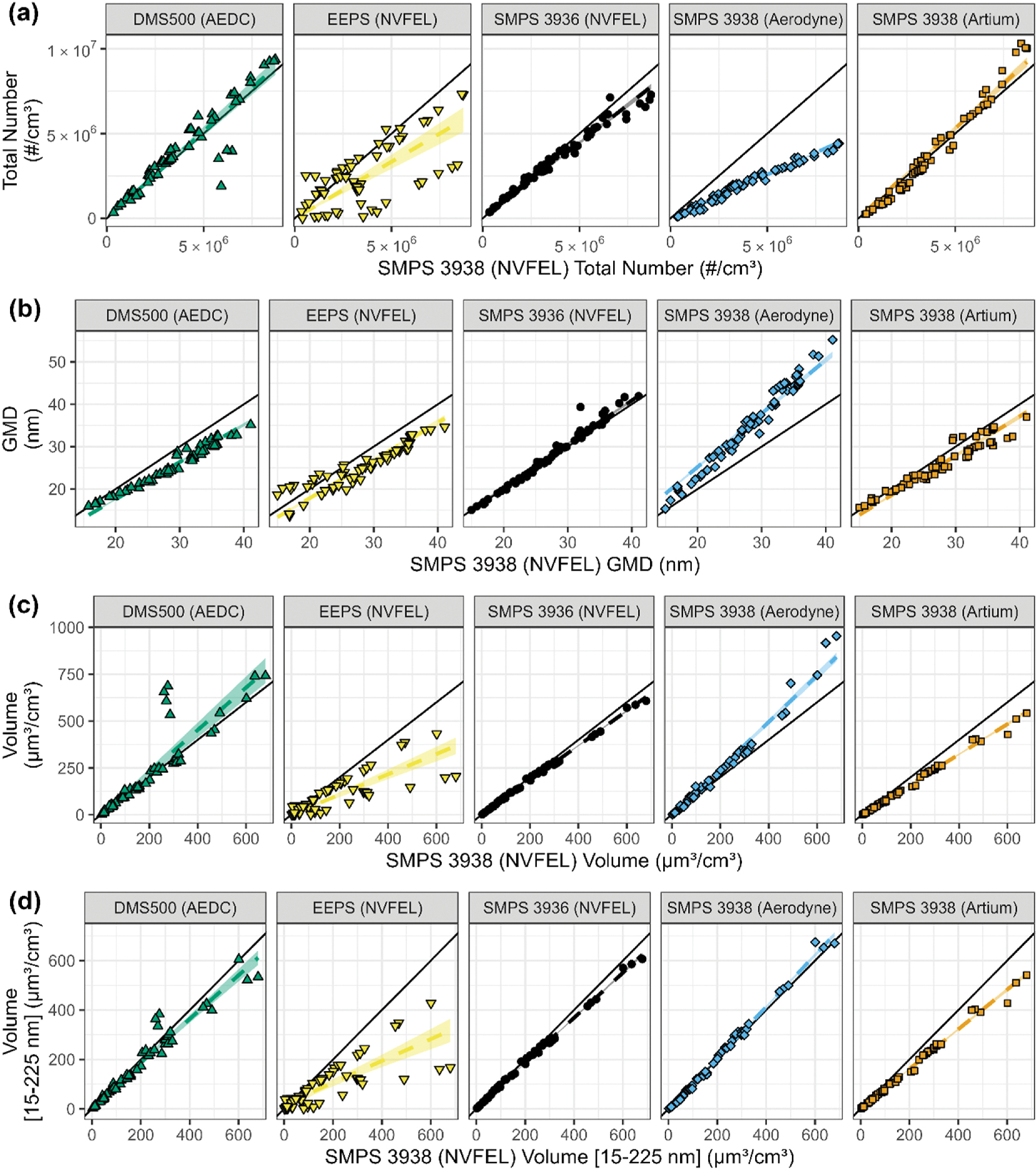
Comparison of particle sizing instruments from VARIAnT 4 by: (a) particle number concentration; (b) geometric mean diameter (GMD) (c) concentration by particle volume of all the particles measured for each instrument’s nominal size range for the J85 and LGT-60 start cart. In panels (a) through (c) the nominal size ranges of each instrument were: DMS size range 4.87 nm–1000 nm, EEPS size range 6.04 nm–523.3 nm, SMPS 3936 (NVFEL) size range 5.94 nm–224.7 nm, SMPS 3938 (Aerodyne) size range 14.6 nm–685.4 nm, and SMPS 3938 (Artium) size range 5.94–224.7 nm. In (d) the computed volume distribution is from 15 to 225 nm, i.e., the range of the instruments are restricted to a common range of 15–225 nm to allow for an unbiased comparison between all instruments. The variability of the EEPS is discussed further in the SI. The instrument owner is listed in parenthesis after each instrument name. In each of the graphs the shaded regions represent a 95% confidence interval for the fit and there is a solid black 1:1 line for reference.

A comparison was made of the six instruments used in VARIAnT 4 all of which measure particle size by electrical mobility. Five of the sizing instruments were located at the end of the plenum and the Artium SMPS was sampling immediately before the plenum. A comparison of total number, geometric mean diameter (GMD) and particle volume measurements made with these instruments is shown in Fig. 5.

In Fig. 5a, the average total number concentration of the EEPS, DMS500, and three of the SMPS instruments were compared to the NVFEL 3938 SMPS, which was used as the reference instrument.^5^ (Note that each SMPS has a unique identifier.) Fig. 5b provides a similar comparison for the GMD of the particles measured. Fig. 5c is a comparison of volume distribution computed for the full range of sizes each instrument was configured to measure.^6^ Fig. 5d is a comparison of the volume distribution computed in the size range of 15–225 nm which was common to all these instruments. Summary statistics for each regression provided in Fig. 5 can be found in Tables S-15 of the SI.

Several observations can be made from the data shown in Fig. 5. For the number concentrations provided in Fig. 5a, the two high flow SMPSs and DMS500 agreed well with each other with the slopes within 11%. The EEPS on the other hand, showed a substantial amount of erratic behavior. For about a quarter of the test points, the EEPS tended to significantly under report number concentration by more than 50% but agreed within 15% for about a third of the points (see SI for test point and daily average comparisons). In the case of the GMD comparisons in Fig. 5b, except for the low flow SMPS, all the instruments generally agreed with slopes within 12% for GMD. In the case of the low flow SMPS, this instrument measured smaller concentrations and larger GMDs as compared to the equivalent high flow analyzers. In fact, the low flow SMPS determined about 50% lower number concentrations. These differences were due to: (1) the low flow SMPS has a minimum sizing range of 15 nm compared to the high flow instrument which has a minimum size range of 6 nm; and (2) the instrument significantly undercounted particles smaller than about 50 nm (e.g., Figure S-19), while agreeing with the other instruments for larger particles. These differences may in part be due to diffusional losses and particle coagulation, for which estimates were made, but do not fully reconcile the undercounting differences. The low flow SMPS, the EEPS and the DMS500 (upper sizing limits 685, 560, and 1000 nm, respectively) sometimes showed a second mode consisting of particles larger than about 150 nm which was barely detectable by the high flow SMPSs (upper sizing limit 225 nm).

Thus, both the DMS500 and the low flow SMPS measured higher volumes than the reference SMPS as shown in Fig. 5c indicating the presence of volume in particles with diameters larger than the reference SMPS was configured to detect. Hence to make a comparison on the same basis among the instruments, the volume was computed for the common size range of 15–225 nm as shown in Fig. 5d.

When comparing volume on the common size, the low flow SMPS showed good agreement with the reference SMPS while the DMS500 measurements were somewhat lower. However, comparing the slopes of the measurements for the entire instrument size range (Fig. 5c) to the slopes for the common size range (Fig. 5d) showed an increase of 21% for the DMS500 and 20% for the low flow SMPS; essentially agreeing, indicating the presence of significant volume in particles larger than 225 nm. We attribute this additional volume to a secondary mode which is discussed below. The secondary particle mode was also observed by the single particle mass spectrometer, miniSPLAT, as discussed in more detail later.

The Artium SMPS which measured the size distribution just before the aerosol entered the sampling plenum was used to monitor possible coagulation due to the residence time in the plenum. The Artium SMPS concentrations and GMDs were consistent with the SMPSs connected to the sampling plenum indicating that coagulation was not likely occurring in the sampling plenum.

Finally, it is worthy of mention that there were slight differences in the size distributions measured by the various sizing instruments as discussed in Section 9 of the SI. The shape of the particle size distributions produced by the various sizing instruments varied slightly as illustrated in Figure S-19 of the SI due to the differing instrument measurement characteristics and inversion methods (see SI for further explanation). However, the general trends of the sizing instruments all agree, even in respect to detection of the secondary size mode discussed in the following sections.

## 4. Source Characterization Results

The particles generated by each DFCAS were characterized according to morphology (VARIAnT 3 and 4), effective density by particle size (VARIAnT 3 and 4), and chemical composition/mass properties according to particle size (VARIAnT 4). As mentioned in the Introduction, particle morphology is discussed in earlier publications (Kumal et al., 2020; Singh, 2019; Singh & Vander Wal, 2019) and thus will not be addressed here. Comparison of carbonaceous PM to total PM and a description of the secondary particle mode are provided in the following sections.

### 4.1. Measurement of carbonaceous particles vs. total particulate matter

**Fig. 6.**
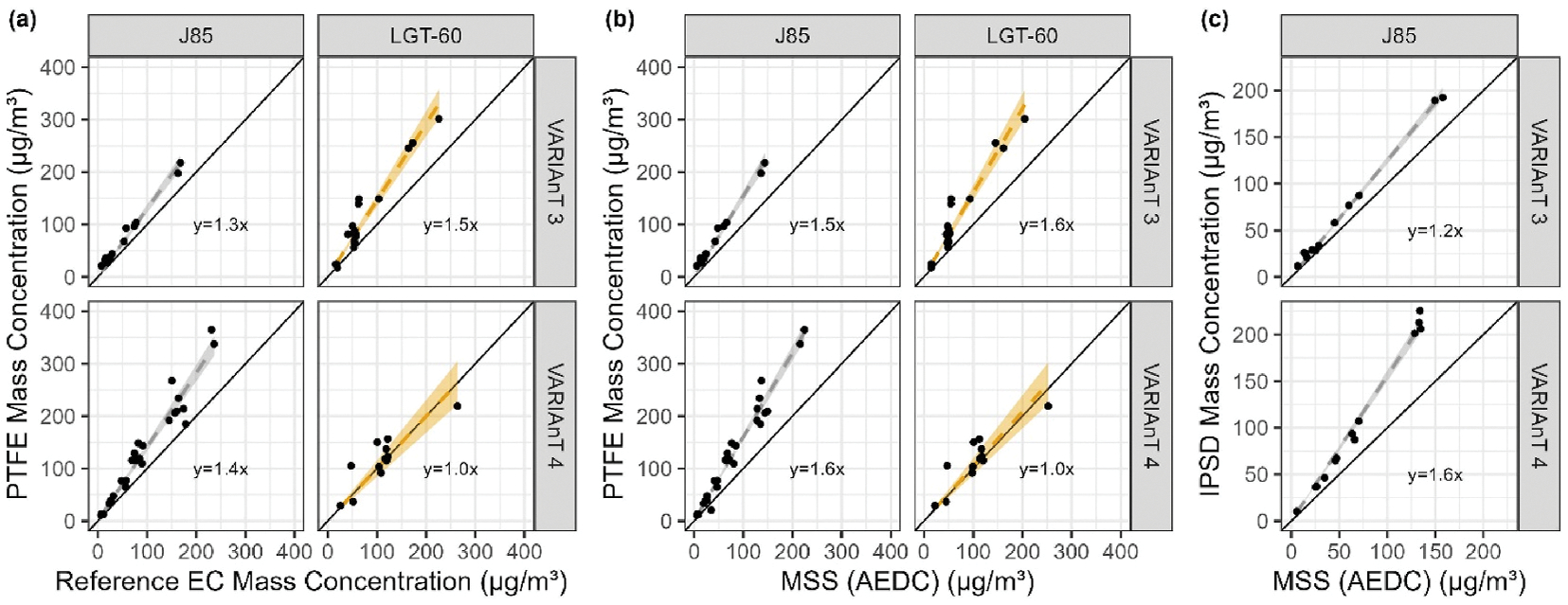
Comparison of the concentrations of EC particles to total PM determined by Teflon filter sampling for the J85 and LGT-60 start cart during VARIAnT 3 and 4. Panel (a) shows data for EC mass and panel (b) present data for BC mass as determined by the AEDC MSS. Also shown in panel (c) are similar comparisons between the integrated particle size distribution (IPSD) mass for particle diameters between 6 and 225 nm, as determined by the DMA-CPMA-CPC, and the AEDC MSS BC mass. In each of the panels the shaded regions represent a 95% confidence interval for the fit and there is a solid black 1:1 line for reference.

When the original ARP6320 was published, an assumption was made by the SAE E–31 Committee that the nvPM produced from aircraft gas turbine engines was composed mainly of black carbon (BC) as measured by the qualified^7^ mass analyzers with only relatively minor amounts of other PM (e.g., metal oxides) present in the engine exhaust. To investigate the difference between the BC mass and total PM (TPM), gravimetric analyses were conducted on the Teflon filters used in the multi-filter sampler during the two campaigns. As a result of this determination, it was found that substantial amounts of additional PM mass not measured by the BC mass analyzers can be present in the aerosol sampled as illustrated in Fig. 6 with the regression statistics provided in Tables S-16 of the SI.

In Fig. 6a, the undiluted concentration of EC is plotted against undiluted TPM as determined from the Teflon filter analyses for the two gas turbines tested in VARIAnT 3 and 4. As shown, the TPM can be as much as ~50% higher than EC depending on campaign and source type. For the J85, the gravimetric mass measurements were 30 and 40% higher than the measured EC mass for VARIAnT 3 and VARIAnT 4, respectively. Similar differences between the MSS measured BC and TPM for all the turbine engine DFCASs is shown Fig. 6b where the BC concentration as determined by the AEDC MSS is plotted against the TPM from the Teflon filters. As shown for the J85, the TPM was 50–60 % higher than the BC measured by the AEDC MSS (Fig. 6b) for the two campaigns.

**Fig. 7.**
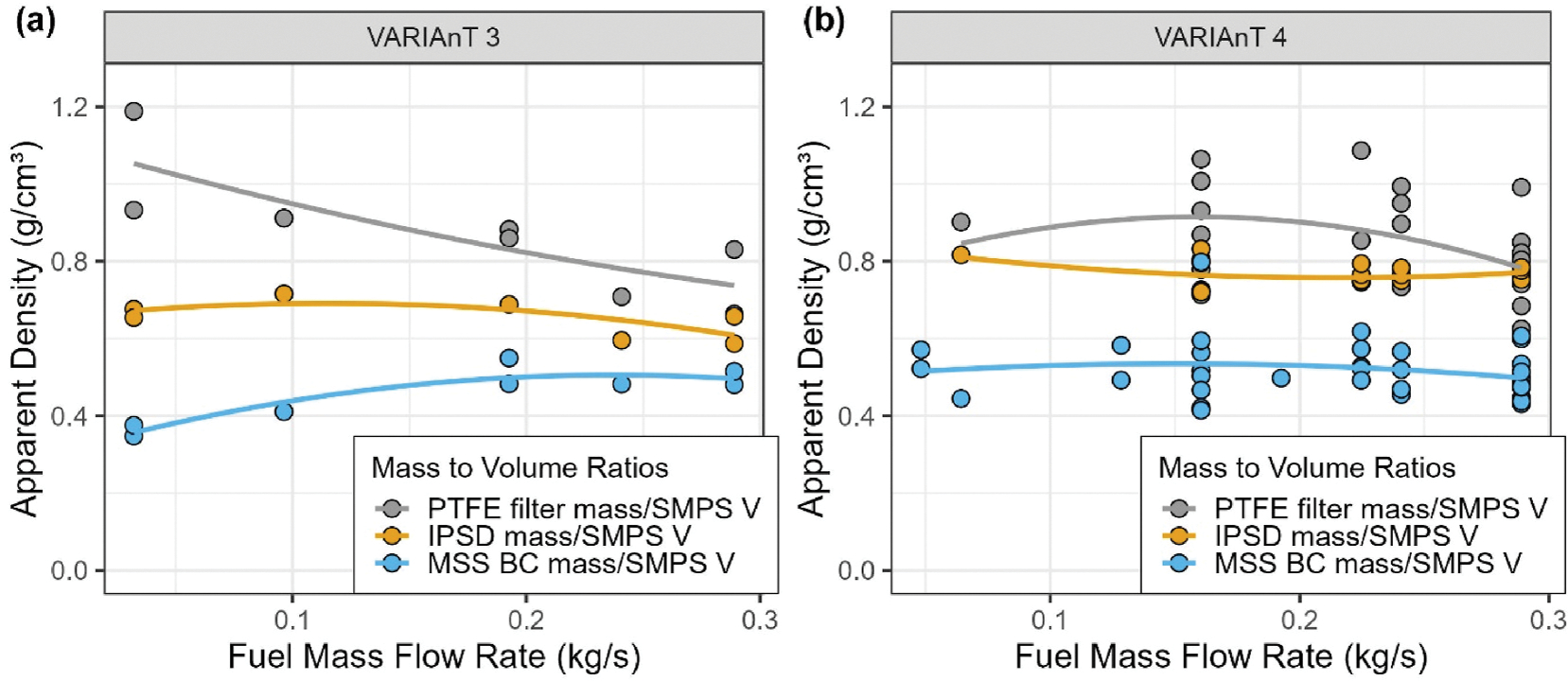
Apparent PM densities for VARIAnT 3 and 4 calculated as the ratios of gravimetric PM mass, IPSD, and MSS (AEDC) masses to SMPS volume (6–225 nm) for the J85 burning Jet-A. These apparent PM densities are plotted versus the Jet-A fuel mass flow rates. Lines are drawn only to guide the readers eyes; data scatter is dependent on the variability observed in day-to-day engine operation (see Section 7.5 of the SI).

Fig. 6c also shows that the PM mass calculated from integrated particle size distribution (IPSD) and size-dependent particle densities as determined by measuring the mass of mobility-selected particles (DMA-CPMA-CPC) during both campaigns. As can be seen from Fig. 6c, the IPSD mass is 20–60 % higher than the MSS-measured BC mass for the two campaigns. As a result, the average apparent PM densities calculated based on the ratio of mass of Teflon filter or IPSD measured PM mass to SMPS volume are higher than those calculated based on the ratio of MSS-measured BC mass to SMPS volume (Fig. 7). These average apparent densities are significantly smaller than the soot material density given the complex shapes and morphologies of these particles. IPSD mass is based on measured size dependent effective density distributions, which, in principle, should include not only BC, but also sulfates, OC, and ash from the fuel and lubrication oil (Abegglen et al., 2016; Drozd et al., 2012; Elser et al., 2019; Fushimi et al., 2019; Onasch et al., 2009; Saitoh et al., 2019, pp. 41–52; Smith et al., 2022; Timko et al., 2010; Timko et al., 2010; Timko et al., 2014). However, it is consistently lower than gravimetric mass. The difference is likely due, at least in part, to the presence of a “secondary mode,” described below, but excluded (except between 150 and 225 nm) from the IPSD calculations. The size range used to calculate IPSD mass was limited to 6–225 nm (NVFEL 3938 SMPS). Larger particles were excluded due to uncertainties in the nature and composition of the secondary mode.

The above results suggest that the measurement of BC alone may not be sufficient to completely quantify the PM mass emissions from aircraft turbines. As will be briefly discussed below, the analysis of single particle composition indicates that in addition to fractal soot particles, the composition of which is dominated by EC, turbine engines can emit larger, more compact particles, that contain ash and high fractions of organic carbon, and can significantly contribute to the measurements of TPM, calculated apparent densities, and measurements by the OCEC analyzer. Additionally, in VARIAnT 3 and 4, the single scattering albedo (SSA, ratio of scattering to extinction) measured by the CAPS PM_SSA_ (Figure S-22) increases with decreasing fuel mass flow rate (or lower PLA) indicating the presence of material in addition to EC.

### 4.2. Secondary mode analysis

Most of the non-volatile particle number emitted by aircraft turbine engines is typically found in the size range from 10 to 100 nm (Zhang, et al., 2019), while most of the mass and volume are typically found between about 25 and 250 nm (Boies et al., 2015; Corbin et al., 2022; Crayford et al., 2012; Durdina et al., 2019; Moore et al., 2015; Timko et al., 2010). Although a distinct coarse mode, consisting of particles larger than a few hundred nm is typically found in roadside (Whitby, et al., 1975) and piston engine tailpipe measurements (Kittelson, 1998), in aircraft exhaust, particles in this size range while sometimes observed (e.g., Corbin et al., 2022; Onasch et al., 2009; Timko et al., 2010) have rarely been examined in detail. In this work a *separate* size mode consisting of particles larger than about 150 nm was observed for some sources and fuels. Three independent observations provide evidence that a secondary mode exists, i.e., various size distributions, miniSPLAT and CAPS ensemble averaged optical properties (single scattering albedo) measurements. These particles will be referred to below as secondary mode particles.

**Fig. 8.**
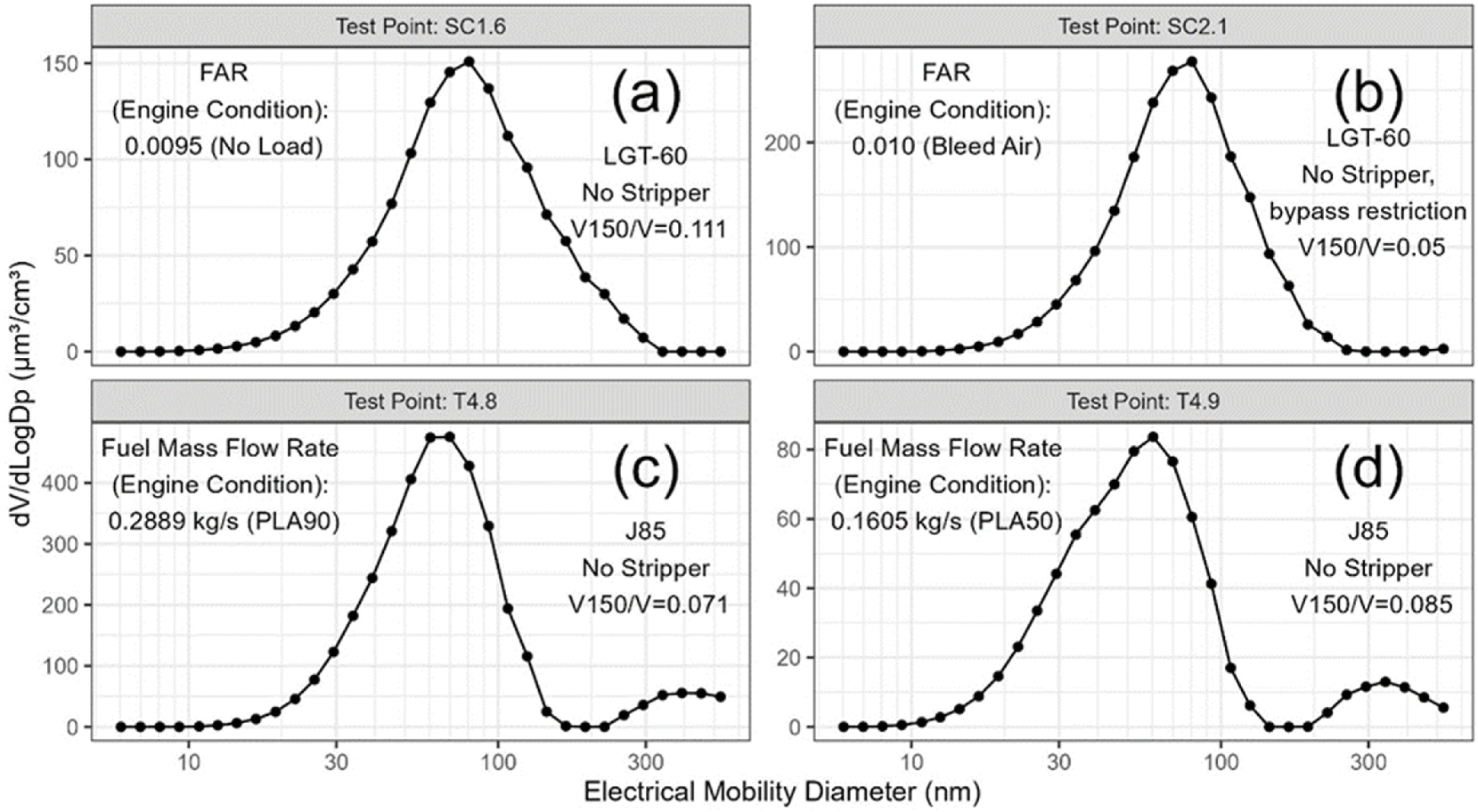
Differential volume (dV/dlogDp) particle size distributions for: the LGT-60 start cart (a) and (b); and J85 (c) and (d) at low and high load operating conditions during VARIAnT 4. Also shown in the caption of each panel is the volume fraction of particles >150 nm in electrical mobility diameter.

**Fig. 9.**
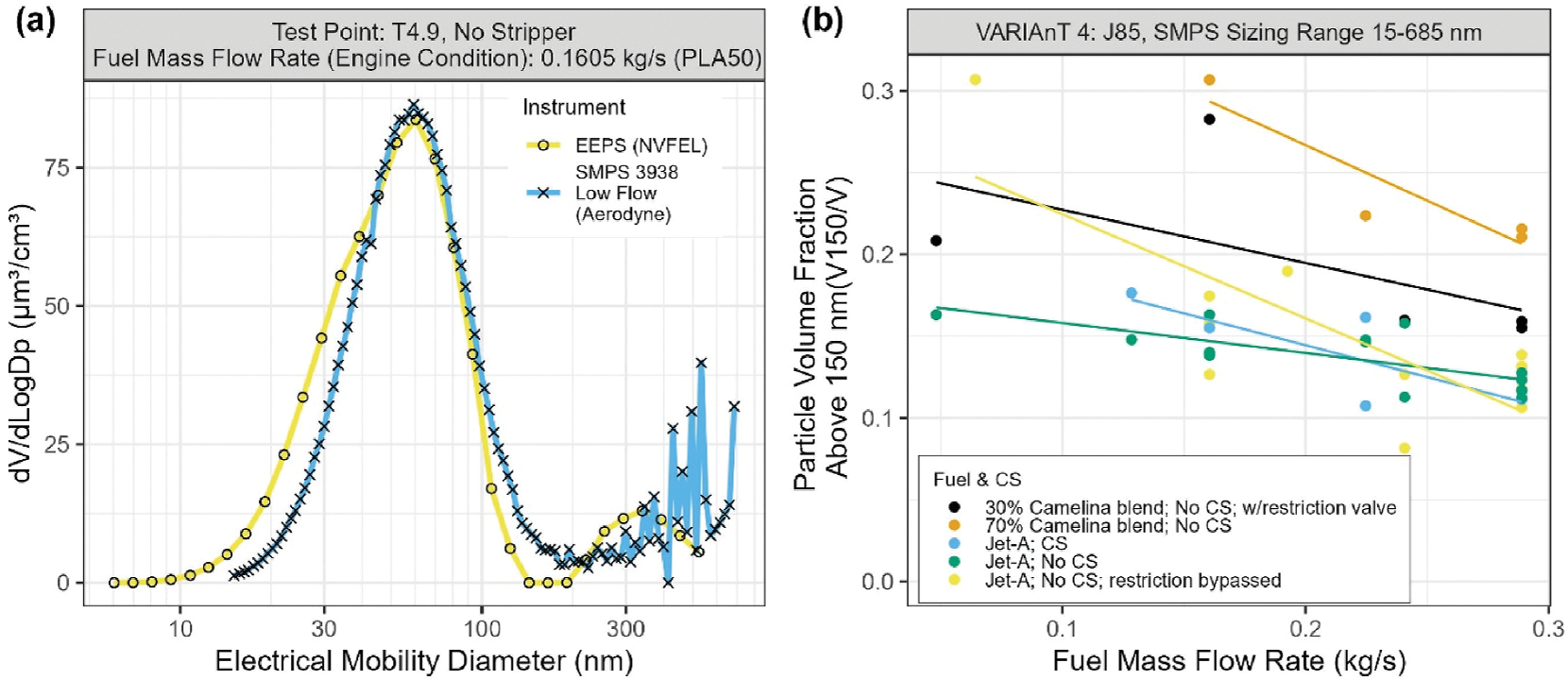
(a) Differential volume (dV/dlogDp) particle size distributions measured by low flow SMPS and EEPS for the J85 engine (fuel flow ratio of 0.160 kg/s), indicating the presence of secondary particle mode; (b) particle volume fraction >150 nm as measured using the low flow SMPS (15–685 nm) as a function of engine power and fuel type for the J85. Lines are drawn only to guide the readers’ eyes.

The presence of the secondary mode is illustrated in the examples shown in Fig. 8 where the volume weighted PSDs measured with the EEPS are plotted for the LGT-60 start cart and J85 operating on Jet-A fuel under low and high load conditions. As shown in Fig. 8a and b for the start cart, a unimodal PSD was observed for the two example test points. On the other hand, for the J85 test points shown in Fig. 8c, d, and 9a, the PSDs are distinctly bimodal with a primary mode similar to that observed for the start cart along with a smaller secondary mode comprised of particles with mobility diameters >150 nm.

When present, the secondary mode was usually detected by all the electrical mobility sizing instruments. Fig. 9a compares volume weighted size distributions measured with the EEPS and the low flow (15–685 nm) Aerodyne SMPS for a typical J85 test. Fig. 9b shows that the volume fraction in the secondary mode increases with decreasing fuel flow rate and with increasing Camelina SAF content in the blended fuel (Table 2). The presence of a secondary particle mode is also associated with an increase in the SSA measured by the CAPS PM_SSA_ (Figure S-22) and appearance of particles with different compositions and morphologies in the miniSPLAT data. Also note that no evidence was found for particle shedding from the cyclone or other parts of the sampling system (e.g., SI Section 7.4) as demonstrated during the daily pre-test zero checks of the system.

We do not have absolute accuracy by which the secondary mode mass can be quantified. Previous studies have suggested that the accuracy of the IPSD method is in the range of 16–23% for a 95% confidence interval (Momenimovahed & Olfert, 2015; Liu, & VasysDettmannSchauerKittelsonSwanson, 2009). There is little information on the use of IPSD in the size range above a few hundred nm, so that the absolute uncertainty in this study could be even higher.

**Fig. 10.**
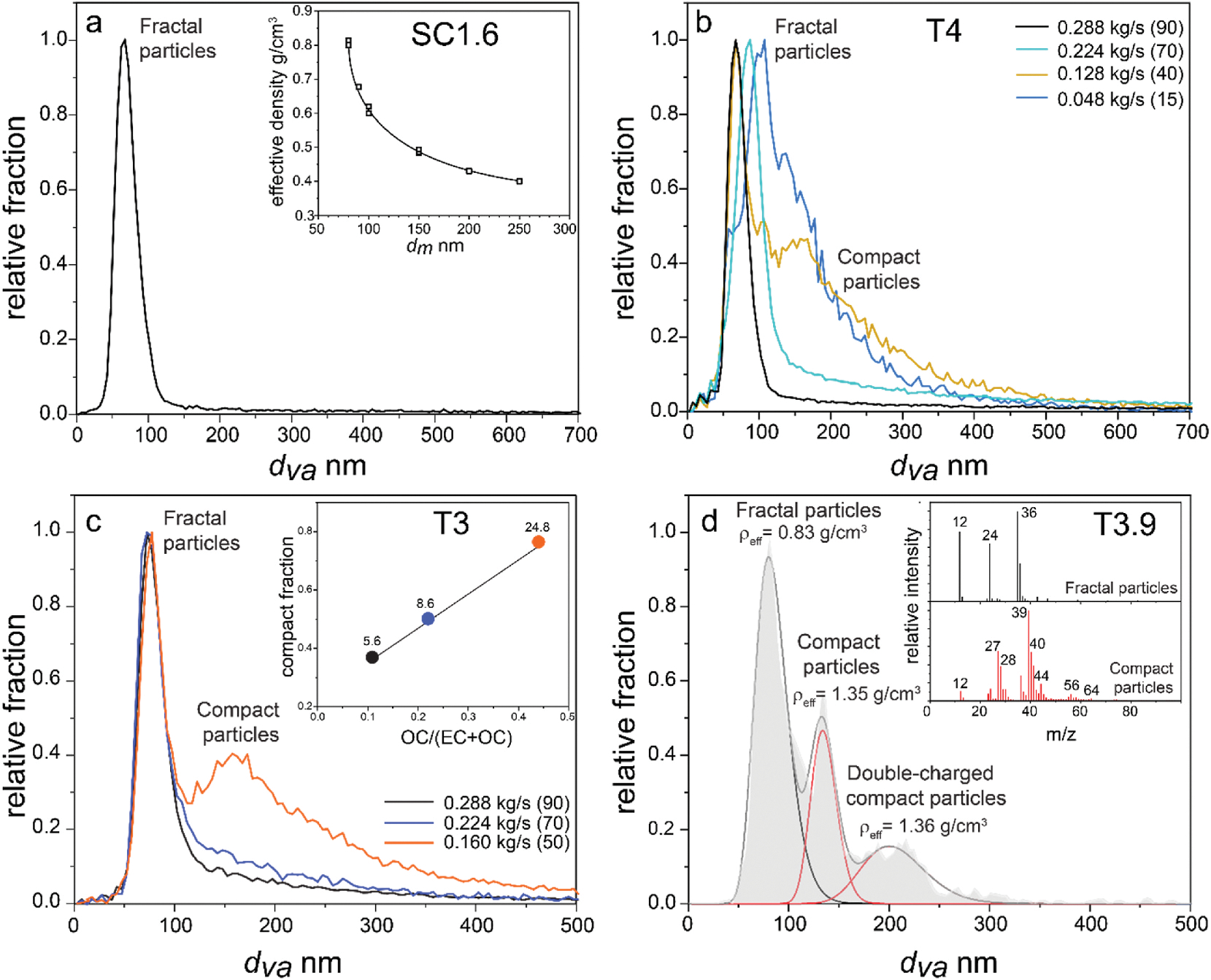
**(a)** MiniSPLAT-measured *d*_*va*_ size distribution of fractal particles generated by the LGT-60 start cart with the effective density of these particles decreasing with increasing particle *d*_*m*_ as shown in the inset; **(b)** *d*_*va*_ size distributions of particles generated by J85 engine operated with Jet-A fuel at different fuel flow rates in kg/s and PLAs in parenthesis, as indicated in the figure legend; **(c)** *d*_*va*_ size distributions of particles generated by J85 engine operated with 70% Camelina/30% Jet-A fuel blend at different fuel flow rates in kg/s and PLAs in parenthesis, as indicated in figure legend. The figure inset shows that the relative fraction of compact particles is increasing with decrease in fuel flow rates and correlates with OC/(EC + OC) ratios and concentrations of gas-phase hydrocarbons indicated by numbers (in units of ppmC) above the datapoints colored to match the *d*_*va*_ size distributions for different fuel flow rates in kg/s and PLAs in parenthesis; **(d)** *d*_*va*_ size distributions of fractal and compact mobility selected particles with *d*_*m*_ = 100 nm and doubly-charged particles with *d*_*m*_ = 151 nm, generated by J85 engine operated with 70% Camelina/30% Jet-A fuel blend at PLA50 (T3.9), which shows that compact particles have higher effective density and contain ash and high fraction of organic carbon (figure inset).

Fig. 10 shows examples of miniSPLAT data for particulates generated by the LGT-60 start cart (Fig. 10a) and J85 engine operated with Jet-A (Figs. 10b) and 70% Camelina/30% Jet-A blend (Fig. 10c and d). Fig. 10a shows that particles emitted by the start cart have unimodal vacuum aerodynamic diameter (d_va_) size distribution, with a narrow linewidth that are indicative of fractal particles whose d_va_s are nearly independent of particle mobility diameters (d_m_) and masses (Zelenyuk et al., 2014). As expected for fractal particles, their effective density, calculated based on the measurements of d_va_ of mobility-selected particles (Zelenyuk et al. 2014, 2017), decreases with increasing particle d_m_ (inset of Fig. 10a). Similarly, miniSPLAT-measured single particle mass spectra indicate that virtually all particles generated by the start cart are composed of EC with very small amounts of OC (mostly oxygenated organics), consistent with EC/(EC + OC) ratio of ~1 measured by the OCEC analyzer and a good agreement between Teflon filter-measured TPM and BC mass observed for the start cart.

In contrast, for J85 tests, especially those operated at low fuel flow rates, miniSPLAT data clearly indicate the presence of both, fractal EC-dominated particles and more compact particles with larger *d*_*va*_ (Fig. 10(b–d)). MiniSPLAT measurements of size- and mass-selected particles indicate that the compact particles have larger sizes (*d*_*va*_*, d*_*m*_*, d*_*a*_), masses, and higher effective densities. The compact particles also have distinctly different mass spectra which indicate the presence of a small amount of ash, with characteristic peaks corresponding to Ca^+^, CaO^+^/Fe^+^, Zn^+^, and a high fraction of organic carbon (inset in Fig. 10d). Fig. 10(b, c) shows that the fraction of particles with larger vacuum aerodynamic diameter (*d*_*va*_) increases with decreasing fuel flow ratios, consistent with the observations of the secondary particle mode by the low flow SMPS and EEPS instruments (Figs. 8 and 9) and an increase in volume fraction of particles >150 nm as a function of engine power and fuel type (Fig. 9b). The insert to Fig. 10c shows that an increase in relative fraction of compact particles, that contain large fractions of organic compounds, is in agreement with the observed increase in measured OC/(EC + OC) ratios and gas-phase concentrations of hydrocarbons (numbers next to the datapoint in ppmC by volume), in accord with previously reported trends of increased OC/(EC + OC) ratios at low thrust levels observed for modern aircraft turbofan engines (e.g., Elser et al. (2019); Corbin et al. (2022); Timko et al., 2014). Similar large compact structures have been reported elsewhere in engines and very rich propane flames (Slowik et al., 2004; Shapiro et al., 2012; Zelenyuk et al., 2017). Fig. 10d shows that compared to fractal EC-dominated particles, compact particles have significantly higher effective densities and, as a result, can significantly contribute to particulate mass loadings, providing supporting evidence regarding the observed differences between TPM and BC mass for J85 engine as compared to those observed for the start cart. The presence of the secondary mode as shown by both the miniSPLAT data and the coarse volume fraction is not influenced by the catalytic stripper, indicating that the OC present is nonvolatile, not removed by the CS at 350 °C.

## 5. Conclusions

A number of conclusions were reached as a result of the study.

1. The MSS and CAPS PM_SSA_ instruments have generally good agreement in their response to EC, with measured mass concentrations falling within ~20% of the reference EC mass concentrations and independent of calibration source used. During VARIAnT 3, all instruments except for the MSSplus were calibrated on a combustor rig at SwRI. The MSSplus during VARIAnT 3 was calibrated by the manufacturer. During VARIAnT 4, all instruments were calibrated using an LGT-60 start cart (see Figs. 2 and 3).
2. For the LII-300, the measured mass concentrations in VARIAnT 3 fall within 18% and in VARIAnT 4 within 30% of the reference EC mass concentration when calibrated on a combustor rig in VARIAnT 3 at SwRI and on an LGT-60 start cart in VARIAnT 4, respectively (see Figs. 2 and 3).
3. All three mass instrument types (MSS, CAPS PM_SSA_, and LII-300) can exhibit different BC to reference EC ratios depending on the emission source (Figs. 2 and 3). The mass instruments also showed the lowest response relative to reference EC for the J85 as compared to the other DFCASs tested.
4. All three mass instrument types exhibit BC to reference EC ratios that appear to be related to particle geometric mean mobility diameter, morphology, or some other parameter associated with particle geometric mean diameter. In most cases, the LII-300s response showed a slightly stronger apparent trend with geometric mean diameter (Fig. 4).
5. Systematic differences in LII-300 measured mass concentrations relative to reference EC have been reduced by calibrating with a turbine combustion source (combustor or turbine engine). Although there was a clear correlation between LII-300 response and EC concentration, the relative response of these instruments can vary significantly depending on the DFCAS (Figs. 2 and 3). Additionally, there are unexplained LII-300 instrument-to-instrument differences observed (see Figs. 2–4).
6. With respect to the particle size measurements made, the sizing instruments were found to be in general agreement in terms of size distributions and concentrations with some exceptions (Fig. 5). For some conditions, the EEPS measured significantly lower concentrations but similar geometric mean diameter and geometric standard deviation for both number and volume. The low flow SMPS consistently underestimated number concentrations but generally agreed in volume concentrations. When a secondary size mode was present, there was a consistency among instruments in its detection.
7. Gravimetric measurements of the aerosol mass produced by the various DFCASs differed from the reference EC, BC, and IPSD measured aerosol masses (Fig. 6). The gravimetric measured mass was substantially greater than both the reference EC and the BC mass for the J85 in both test campaigns. In VARIAnT 3 the LGT-60 start cart aerosol mass emissions measured with the Teflon filters was substantially higher than the BC mass. In VARIAnT 4 for the LGT-60 start cart the differences were less clear due to the large variability. The slope from fitting the gravimetric mass to reference EC mass and gravimetric mass to BC mass was 1.0, but each relationship had larger variability compared to the other fits in Fig. 6 as demonstrated by the spread of the 95% confidence interval of the fit. This variability is also shown by the standard deviations in the average Teflon mass to reference EC mass ratios and the average Teflon mass to BC mass ratios. These mass ratios and standard deviations were 1.14 ± 0.40 and 1.19 ± 0.39, respectively. The mass determined by IPSD for the J85 was substantially larger than the BC mass in both VARIAnT 3 and VARIAnT 4. IPSD results do not include the secondary mode as the effective density of that material is unknown.
8. For many test conditions, especially at low engine loads and low particle concentrations, a large or secondary particle mode >150 nm was often observed by the SMPS, EEPS, DMS500 (Figs. 8 and 9), and miniSPLAT (Fig. 10). In some cases, there was substantial particle volume present in this mode (Fig. 9b). The miniSPLAT measurements also indicated the presence of large particles having more compact morphologies, higher effective density, and contain ash and a higher fraction of organic carbon (Fig. 10). This increased large particle fraction is also associated with higher values of single scattering albedo measured by the CAPS PM_SSA_ instrument and higher OC fraction measured by the OCEC instrument (Figure S-22). Therefore, the large particle mode can significantly add to the mass of particles measured in the emissions. More detailed examination of this secondary mode will be discussed in a forthcoming paper.
9. From the presented particle size, mass, optical properties, and composition measurements, the nvPM composition can be more complicated than original E–31 assumptions (SAE International, 2012) that the nvPM emissions were comprised of mainly BC (Figs. 6 and 7). Since the BC mass instruments are not directly sensitive to the additional non-BC PM mass, a fraction of the “nvPM” is not being measured.

In summary, mass instrument comparisons indicate multiple instruments which convert light absorption to a BC mass concentration can have very good agreement on most sources. The MSS and CAPS PM_SSA_ instruments with a single transduction method of converting optical absorption to BC mass concentration show good agreement for all cases studied here. The performance of LII-300 instruments differed with each other. All instruments show some instrument response dependence on particle size distribution geometric mean diameter and should be expected in terms of the light scattering cross section dependence on particle diameter. However, the relative difference between the LII-300 response and EC decreases rather strongly with decreasing particle geometric mean diameter below approximately 35 nm or some other property of the soot. Resolving the physical mechanism for this would need a more focused study of the LII-300.

This study has been unique in its ability to measure aircraft PM emissions by multiple metrics which have allowed for a detailed description of a secondary mode. The measurements all indicate the nvPM emissions from this J85 turbine engine have a nontrivial fraction of non-BC, especially at lower engine thrusts, comparable with other aircraft engine studies (see references in Source Characterization Results). Further investigation is warranted to determine if these emissions characteristics are more common in modern engines. If so, the BC optical absorption type instruments are not measuring all nvPM emissions as currently defined by SAE E–31 and thus may warrant reconsideration by the SAE E–31 Committee.

## Supplementary Material

supplementary material

figure data

## Figures and Tables

**Table 1:** Aerosol sources, fuels, and number of test series conducted in each test campaign.

Campaign	DFCAS Type^a^	Fuel Type (Number of Test Series Conducted)			

VARIAnT 3 (1/9–1/31/17)	Cummins ISX15 Heavy Duty Diesel^d^	Certification Diesel^b^ (12)			
VARIAnT 3 (3/2–3/16/17)	General Electric J85-GE-5 Turbojet Engine	Jet-A (2)	45% Camelina/Jet-A Blend (1)	70% Camelina/Jet-A Blend (1)	
	Libby Welding (AiResearch) LGT-60 Turbine Start Cart	Jet-A (2)	45% Camelina/Jet-A Blend (1)	70% Camelina/Jet-A Blend (1)	49% HDRD/Jet-A Blend^b^ (1)
	Isuzu 4LE2T Diesel Generator Set ^e^	100% HDRD^c^ (1)			
VARIAnT 4 (8/9–8/21/18)	General Electric J85-GE-5 Turbojet Engine	Jet-A (6)	30% Camelina/Jet-A Blend (1)	70% Camelina/Jet-A Blend (1)	
	Libby Welding (AiResearch) LGT-60 Turbine Start Cart^f^	Jet-A (2)			
	Jing Model 5201 & 6200 miniCAST Burners	Propane (1)			

a DFCAS = diffusion flame combustion aerosol source. A description of each is provided in the SI.

b Fuel specification per Title 40 Code of Federal Regulations Chapter I Subchapter U Part 1065 Subpart H.

c HDRD = hydrogenation-derived renewable diesel fuel.

d These engine-out emissions tests were conducted at EPA’s National Vehicle and Fuel Emissions Laboratory in Ann Arbor, MI. All other testing was conducted at the UTSI Propulsion Research Facility operated by AEDC.

e Engine-out emissions only.

f This unit was also used for the VARIAnT 4 pre-test instrument calibration performed 7/8–7/23/18 as described below.

**Table 2:** Typical fuel analyses.

Campaign	Fuel Type^a^	Carbon (Weight Percent)^c^	Hydrogen (Weight Percent)^d^	Sulfur (ppm mass)^e^	Net Heat of Combustion (kJ/ kg)^f^

VARIAnT 3	Certification Diesel^b^	86.66	13.05	9.5	42,864
	Jet-A	85.91	14.09	220	43,319
	45% Camelina/Jet-A Blend	85.68	14.30	170	43,396
	70% Camelina/Jet-A Blend	85.29	14.70	120	43,699
	49% HDRD/Jet-A Blend	85.47	14.52	110	43,668
	100% HDRD	84.89	15.10	60	43,906
VARIAnT 4	Jet-A	85.88	14.10	227	43,359
	30% Camelina/Jet-A Blend	85.47	14.51	180	43,543
	70% Camelina/Jet-A Blend	85.24	14.75	150	43,729

a HDRD = hydrogenation derived renewable diesel fuel. All alternative fuels are blended with military grade Jet-A.

b Typical analysis for diesel engine certification fuel per Title 40 Chapter I, Subchapter U, Part 1065, Subpart H (EPA, n. d.).

c Determined by calculation.

d Determined by ASTM D3701.

e Determined by ASTM D4294.

f Determined by ASTM D4809, numbers given are as reported.

**Table 3:** Specifications of the measurement equipment used during VARIAnT 3 and 4.

Measured Parameter(s)	Principle of Operation	Instrument Make/Model	Campaign and Number of Analyzers^a^	
			V3	V4

Total particle mass	Teflon filter sampling + gravimetric analysis; 1 standard deviation of filter loading ~1 μg	Multifilter Sampler w/25-mm Teflon and backup quartz filters	1	1
Black carbon mass concentration	Optical light absorption (photoacoustic soot sensing)	AVL Model 483 Micro Soot Sensor (MSS); 0.001–300 mg/m^3^, detection limit ~0.005 mg/m^3^	1	1
		AVL Model 483 Micro Soot Sensor Plus (MSSplus); 0.001–150 mg/m^3^, detection limit ~0.001 mg/m^3^	1 or 2^b^	1
	Optical light absorption (laser-induced incandescence)	Artium Technologies LII-300 Laser Induced Incandescence Analyzer (LII-300)^d^; 0.0002–20,000 mg/m3	2	2–4^c^
	Optical light absorption, i.e., extinction minus scattering (Cavity Attenuated Phase Shift)	Aerodyne CAPS PMSSA ^e^; 0–500 μg/m^3^, detection limit <0.5 μg/m^3^ (3 σ, 1 s)	1	1
Organic and elemental carbon (OCEC)	Thermal-optical transmittance (ToT)	Sunset Model 4 Semi-Continuous OCEC Analyzer	1	1
	Filter sampling + thermal-optical transmittance (ToT); filter loading quantification lower limit ~0.3 mg/m^2^	Multifilter Sampler w/25-mm quartz filters + Sunset Laboratory Model 4 L OCEC Analyzer	1	1
		Multifilter Sampler w/25-mm Teflon and backup quartz filters + Sunset Laboratory Model 4 L OCEC Analyzer^f^	1	1
Total particle number concentration	Catalytic stripper + condensation particle counting	AVL Particle Counter (APC) Aviation; 0 to 10,000 particles/cm^3^; lower detection limit ~0.001 particles/ cm^3^		1
Particle size distribution	Differential electrical mobility analysis + condensation particle counting; concentration range: 0–10^7^ particles/cm^3^; particle mobility diameter range: 3 -	TSI Model 3936 Scanning Mobility Particle Sizer (Model 3081 differential mobility analyzer + Model 3776 condensation particle counter)	1	1
		TSI Model 3938 Scanning Mobility Particle Sizer (Model 3081 differential mobility analyzer + Model 3776 condensation particle counter)	1	3^g^
	Differential electrical mobility analysis + electrometer particle counting	TSI Model 3090 Engine Exhaust Particle Sizer (EEPS); mobility diameter range: 5.6–560 nm; 1 s average concentration lower limits: ~500 particles/cm^3^ at 5.6 nm and ~5 particles/cm^3^ at 560 nm	1	1
		Cambustion Model DMS500 Fast Particle Analyzer mobility diameter range: 5–1000 nm; 1 s average concentration RMS noise: ~10^4^ particles/cm^3^ at 5.6 nm and ~200 particles/cm^3^ at 560 nm		1
Grids for analysis by transmission electron microscopy (TEM)	Electrostatic precipitator + electrometer particle counting	Naneos Partector; concentration range: 0–20,000 μm^2^/cm^3^; lower detection limit ~1 μm^2^/cm^3^	2	1
Particle characterization	Particle effective density by electrical mobility diameter + charge/mass measurement	Model 3081 differential mobility analyzer + Cambustion Centrifugal Particle Mass Analyzer (CPMA; mass range: 0.0002–1050 fg) + Model 3025a condensation particle counter	1	1
	Aerodynamic diameter analysis	Cambustion Aerodynamic Aerosol Classifier (AAC)^I^; aerodynamic diameter range: 25–5000 nm		1
	Cascade impaction w/on-line gravimetric analysis	TSI Model 140 Quartz Crystal Microbalance/Micro-Orifice Uniform Deposit Impactor (QCM/MOUDI)^h^		1
Particle chemical composition	Size-resolved single particle mass spectrometry	PNNL single particle mass spectrometer (miniSPLAT)		1
Carbon dioxide in diluted sample	Nondispersive infrared (NDIR) analysis	CAI 601; Noise <1% of full scale of factory calibrated range	1	
		ABB EL-3020; lower detection limit (4 standard deviations): 4% of full scale of range		1
Carbon dioxide (raw)	Fourier transform infrared spectroscopy (FTIR)	MKS 2030 MultiGas Analyzer; lower detection limit: ~0.005%	1	1
Carbon monoxide	Fourier transform infrared spectroscopy (FTIR)	MKS 2030 MultiGas Analyzer; lower detection limit: ~0.5 ppm	1	1
Sulfur dioxide	Fourier transform infrared spectroscopy (FTIR)	MKS 2030 MultiGas Analyzer; lower detection limit: ~0.6 ppm	1	1
Total hydrocarbons	Flame ionization detector	CAI 300 HFID Hydrocarbon Analyzer; resolution: 0.3 ppm Carbon	1	1
Nitrogen oxides	Fourier transform infrared spectroscopy (FTIR)	MKS 2030 MultiGas Analyzer; lower detection limit: ~0.5 ppm	1	1

a V3 = VARIAnT 3 campaign; V4 = VARIAnT 4 campaign. The number indicates how many analyzers were used in that campaign and a blank indicates the instrument was not used.

b The laser wavelength used in the MSS is 808 nm. Note that in the AEDC portion of VARIAnT 3, a second MSSplus was added to the instrument suite at the last minute. Unlike the original MSS and MSSplus, this analyzer was calibrated at AVL in Gratz, Austria using a miniCAST laboratory burner.

c Number of instruments varied during the campaign due to functional problems.

d The laser wavelength used in the LII-300 is 1064 nm. Not all four analyzers were on-line at each test point due to functional problems.

e The CAPS PM_SSA_ instrument operated at a wavelength of 630 nm in VARIAnT 3 and 780 nm in VARIAnT 4. The CAPS PM_SSA_ measures extinction and scattering coefficients and derives the absorption coefficient from the difference. Black carbon (BC) mass concentrations (μg/m^3^) are obtained by dividing the absorption coefficients (Mm^−1^) by the wavelength-dependent MAC (m^2^/g).

f Teflon/quartz filter train used for gas phase OC artifact correction plus the determination of total nvPM mass concentration.

g Note that two of these SMPSs operated at a sample flow rate of 1.5 lpm and sheath flow of 15 lpm with the third (referred to here as “low flow”) operated at 0.3 lpm and sheath flow of 3.0 lpm. The low flow instrument was intended to investigate the presence of larger particles in the aerosol sampled. The low flow mode SMPS size range was 17 nm–680 nm and the high flow range was 6 nm–225 nm.

h No useful information obtained from this instrument.

i Data from the Cambustion AAC data will be reported in a subsequent paper.

## Footnotes

1 During VARIANT 4, tests were also run in the CPMA/SMPS configuration which sometimes gave somewhat different density results. These tests will be described in a subsequent publication.

2 Note that AIR6241 is the background document for ARP6320A which was published after VARIAnT 1 and 2.

3 APR6320A includes a 1 μm cut-point aerodynamic diameter cyclone which was included in this sampling system, but the VARIANT 3 and 4 sample flow rates produced the other cut-points during the tests which ranged from 0.6 to 4 μm.

4 The slopes from the linear fits reduce to the mean ratios of y-axis values to x-axis values only if the variability in each of the y-axis values is proportional to the corresponding x-axis value.

5 The 3938 NVFEL SMPS selected as the reference instrument as it was the most modern of the instruments and agreed with the NVFEL 3936 SMPS. Both were at the same location as the other sizing instruments (except for the Artium 3938 SMPS which was upstream of the plenum) and mass instruments.

6 Measured number distributions were converted to volume distributions then summed over all bins to compute the volume for each test point. The formula for this conversion is shown in SI-13.

7 Qualified refers to an instrument that has demonstrated conformity to the performance specifications of ARP6320.
