# Supplementary material for: Evaluation of methods for characterizing the fine particulate matter emissions from aircraft and other diffusion flame combustion aerosol sources
Source: J Aerosol Sci. Author manuscript; Available in PMC 2024 May 15. (PMC11095129; doi:10.1016/j.jaerosci.2024.106352)
Supplement: supplementary material [file NIHMS1984390-supplement-supplementary_material.docx]

SUPPORTING INFORMATION

**Evaluation of Methods for Characterizing the Fine Particulate Matter Emissions from Aircraft and Other Diffusion Flame Combustion Aerosol Sources**

Robert Giannelli, Jeffrey Stevens, John S. Kinsey, David Kittelson, Alla Zelenyuk, Robert Howard, Mary Forde, Brandon Hoffman, Cullen Leggett, Bruce Maeroff, Nick Bies, Jacob Swanson, Kaitlyn Suski, Gregory Payne, Julien Manin, Richard Frazee, Timothy B. Onasch, Andrew Freedman, Imad Khalek, Huzeifa Badshah, Daniel Preece, Vinay Premnath, and Scott Agnew

# Project Participants

The following organizations and individuals participated in each test campaign:

VARIAnT 3:

- U. S. Environmental Protection Agency National Risk Management Research Laboratory [NRMRL]--John Kinsey
- U. S. Environmental Protection Agency National Vehicle and Fuel Emissions Laboratory [NVFEL]--Bob Giannelli, Nick Bies, Jeff Stevens, and Scott Agnew
- U. S. Air Force, Arnold Engineering Development Complex (AEDC)--Robert Howard, Brandon Hoffman, Brad Winkleman, Robert Baltz, Mary Forde, Todd VanPelt, and Test Team
- Artium Technologies--Greg Payne and Will Bachalo
- AVL Test Systems, Inc.--Richard Frazee
- Aerodyne Research--Tim Onasch and Andrew Freedman
- University of Minnesota--David Kittelson
- Southwest Research Institute [SwRI]--Imad Khalek, Huzeifa Badshah, Daniel Preece, and Vinay Premnath
- WMS Engineering--Bill Silvis

VARIAnT 4:

- U. S. Environmental Protection Agency National Risk Management Research Laboratory [NRMRL]--John Kinsey
- U. S. Environmental Protection Agency National Vehicle and Fuel Emissions Laboratory [NVFEL]--Bob Giannelli, Jeff Stevens, Cullen Leggett and Nick Bies
- U. S. Air Force, Arnold Engineering Development Complex (AEDC)--Robert Howard, Mary Forde and Test Team
- U. S. Department of Energy, Pacific Northwest National Laboratory (PNNL)--Alla Zelenyuk and Kaitlyn Suski
- Artium Technologies--Greg Payne and Julien Manin
- Singularity Scientific--Richard Frazee
- Aerodyne Research--Tim Onasch and Andrew Freedman
- University of Minnesota--David Kittelson and Jake Swanson
- Penn State University--Randy Vander Wal and Madhu Singh
- Instrument loans--Honeywell (D. Christie); NASA-Glenn (J. Klettlinger); TSI (R. Anderson); and Cambustion (C. Nickolaus); Naneos (D. Meier); Catalytic Instruments (J. Swanson)

# Test Matrix

As discussed in the main text, a total of 40 test series were performed during VARIAnT 3 and 4. Table S-1 provides details of each test series conducted in each study according to DFCAS type, date, test point number, fuel type, run condition, and test objective. Table 2 of the main text provides information on typical fuel properties.

Table S-1. VARIAnT 3 and 4 Experimental Matrix

| **Test Campaign** | **DFCAS Tested** | **Date** | **Test ID** | **Fuel Type^a^** | **Run Conditions^b^** | **Test Objective** |
| --- | --- | --- | --- | --- | --- | --- |
| **VARIAnT 3** | Cummins ISX15 Diesel | 1/19/17 | D1 | Certification Fuel | 1500 rpm @ 10, 20, and 40% torque | Scoping |
|  |  | 1/20/17 | D2 | Certification Fuel | 1200 rpm @ 20% torque | Scoping |
|  |  | 1/21/17 | D3 | Certification Fuel | 1200 rpm @ 20% torque | Instrument comparison |
|  |  | 1/22/17 | D4 | Certification Fuel | 1200 rpm @ 20% torque | Instrument comparison |
|  |  | 1/24/17 | D5 | Certification Fuel | 1200 rpm @ 20% torque | Instrument comparison |
|  |  | 1/25/17 | D6 | Certification Fuel | 900 rpm @ 60% torque | Instrument comparison |
|  |  | 1/26/17 | D7 | Certification Fuel | 1500 rpm @ 0% torque | Instrument comparison |
|  |  | 1/27/17 | D8 | Certification Fuel | 1200 rpm @ 20% torque | Instrument comparison |
|  |  | 1/28/17 | D9 | Certification Fuel | 900 rpm @ 60% torque | Instrument comparison |
|  |  | 1/29/17 | D10 | Certification Fuel | 1500 rpm @ 0% torque | Instrument comparison |
|  |  | 1/30/17 | D11 | Certification Fuel | 1500 rpm @ 0% torque | Instrument comparison |
|  |  | 1/31/17 | D12 | Certification Fuel | 1500 rpm @ 0% torque | Instrument comparison |
|  | GT-05 Start Cart | 3/2/17 | S1 | Jet-A | No load (FAR = 0.0071) | Scoping |
|  |  | 3/3/17 | C1 | Jet-A | No load (FAR = 0.0076) | Certification Study |
|  |  | 3/4/17 | C2 | Jet-A | No load (FAR = 0.0078) | Certification Study |
|  |  | 3/5/17 | C3 | Jet-A | Load bank (FAR = 0.0090) or bleed air (FAR = 0.0147) | Certification Study |
|  |  | 3/6/17 | C4 | Jet-A | Bleed air (FAR = 0.0148) | Certification Study |
|  | LGT-60 Start Cart | 3/7/17 | IC1 | Jet-A | No load (FAR = 0.0091) | Instrument comparison |
|  |  | 3/8/17 | IC2 | Jet-A | No load (FAR = 0.0090) or bleed air (FAR = 0.141) | Instrument comparison |
|  |  | 3/9/17 | IC3 | 49% HDRD | No load (FAR = 0.0080) or bleed air (FAR = 0.0103) | Instrument comparison |
|  |  | 3/10/17 | IC4 | 45% Camelina | No load (FAR = 0.0074) or bleed air (FAR = 0.0099) | Instrument comparison |
|  | J-85-GE-5 | 3/11/17 | T1 | Jet-A | 15 PLA | Instrument comparison |
|  |  | 3/12/17 | T2 | Jet-A | 15, 40, 75, and 90 PLA | Instrument comparison and applicability |
|  |  | 3/13/17 | T3 | 45% Camelina | 10, 15, 25, 60, and 90 PLA | Instrument comparison |
|  | Isuzu 4LE2T  Diesel Generator | 3/14/17 | D13 | 100% HDRD | No load and 20 kW load @ 1800 rpm | Instrument comparison |
|  | J-85-GE-5 | 3/15/17 | T4 | 70% Camelina | 10, 30, 40, and 60 PLA | Instrument comparison |
|  | LGT-60 Start Cart | 3/16/17 | IC5 | 70% Camelina | Bleed air (FAR = 0.0150) or no load (FAR = 0.0138) | Instrument comparison |
|  |  |  |  |  |  |  |
| **VARIAnT 4** | LGT-60 Start Cart | 8/9/18 | SC1 | Jet-A | No load (FAR = 0.0095) | Calibration verification |
|  | J-85-GE-5 | 8/10/18 | T1 | Jet-A | 50 and 75 PLA | Applicability |
|  | Jing 6200 Mini-CAST | 8/11/18 | C1 | Propane | 25 ml/min propane; 0 mixing N_2_; 0.55 L/min oxidation air; 5 L/min dilution air; 2 L/min quench N_2_ | Instrument comparison |
|  | Jing 5201 Mini-CAST | 8/12/18 | C2 | Propane | 60 ml/min propane; 0 mixing N_2_; 1.55 L/min oxidation air; 20 L/min dilution air; 7 L/min quench N_2_ | Instrument comparison |
|  | J-85-GE-5 | 8/13/18 | T2 | Jet-A | 50, 75, and 90 PLA | Applicability |
|  |  | 8/14/18 | T3 | 70% Camelina | 50, 60, 70, 80, and 90 PLA | Survey |
|  |  | 8/15/18 | T4 | Jet-A | 15, 40, 50, 70, and 90 PLA | Survey |
|  |  | 8/16/18 | T5 | 30% Camelina | 15, 50, 75, and 90 PLA | Applicability |
|  |  | 8/17/18 | T6 | Jet-A | 15, 20, 30, 40, 50, 70, 80, and 90 PLA | Scoping + steady state operation |
|  | Jing 5201 Mini-CAST | 8/18/18 |  | Propane | Catalytic stripper between DMA and CPMA/miniSPLAT | CPMA/miniSPLAT testing |
|  | LGT-60 Start Cart | 8/19/18 | SC2 | Jet-A | Bleed air (FAR = 0.010)  Catalytic stripper between DMA and CPMA/miniSPLAT | Steady state operation |
|  | J-85-GE-5 | 8/20/18 | T7 | Jet-A | 20, 50, and 75 PLA | Scoping + steady state operation |
|  |  | 8/21/18 | T8 | Jet-A | 50, 60, 75, and 90 PLA | Applicability |

^a^ All blends with military specification Jet-A. HDRD = hydrogenation derived renewable diesel fuel. Aviation fuels which are comprised of at least 50% Jet-A by volume are considered “drop-in” aviation fuels. ASTM International (ASTM) D7566-22a specifications for sustainable aviation fuels (SAFs) allows for blends of 10% to 50% with Jet A (or Jet A-1) at least 50% of the blend by volume. [ASTM International. (2022). Standard Specification for Aviation Turbine Fuel Containing Synthesized

Hydrocarbons. D7566-22a, West Conshohocken, PA]

^b^ Load bank = electrical load on start cart; Bleed air = bleed air extraction from start cart; FAR = average fuel-to-air ratio; and PLA = power lever angle in degrees for J-85. See Table S-4 for nominal fuel flow for the various PLA settings.

# Test Facilities and Aerosol Sources

VARIAnT 3 testing of the Cummins Model ISX15 Heavy Duty Diesel in January 2017 was conducted in Test Cell HD2 at the U. S. Environmental Protection Agency’s National Vehicle and Fuels Emissions Laboratory (NVFEL) in Ann Arbor, MI. Figure S-1 shows a photo of the equipment installed in the test cell with specifications of the Cummins engine shown in Table S-2 below.


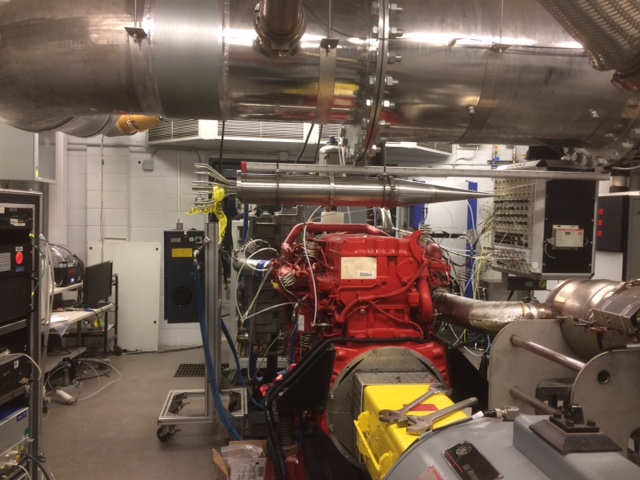

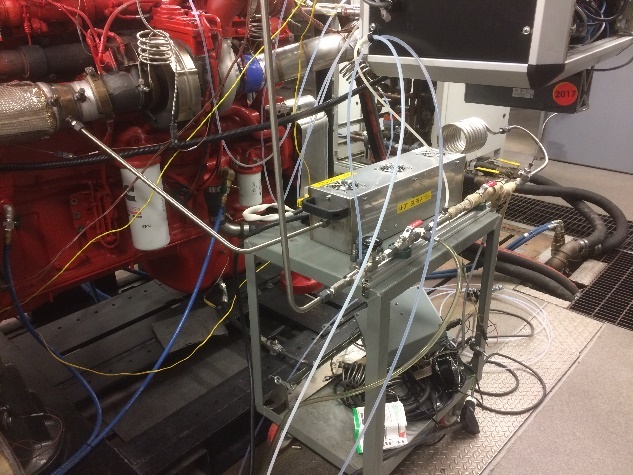


Figure S-1. Photos of test cell at NVFEL.

Table S-2. Specifications for the Cummins Model ISX15 Heavy Duty Diesel.

| **Characteristic or Operating Parameter** | **Specification** |
| --- | --- |
| Engine model | ISX15 |
| Number of cylinders | 6 |
| Bore | 102 mm |
| Stroke | 120 mm |
| Total displacement | 14.9 L |
| Curb idle speed @ 1242.9 Nm torque and 78.1 kW | 600 rpm |
| Peak power @ 1520 rpm and 1962.4 Nm torque | 312.3 kW |
| Peak torque @ 1162 rpm and 246.8 kW | 2027.9 Nm |

Testing at the University of TN Space Institute (UTSI) was conducted at its Propulsion Research Facility located on the property of the Arnold Engineering Development Complex (AEDC) at Arnold Air Force Base, TN. Figure S-2 shows a diagram of the facility and the location of the J85-GE-5 turbojet engine (J85) used during VARIAnT 3 and 4. General specifications of the test engine are shown in Table S-3. The J85 was run over a range of thrust settings associated with a power level angle (PLA). The relationship between PLA and typical normalized thrust (non-afterburning) and fuel flow is shown in Table S-4 for VARIAnT 4.

Also tested in VARIAnT 3 and 4 were two turbine powered start carts as described in Table S-5. This equipment was operated either without load, with bleed air extraction, or with electrical power generation. The engine load was characterized by the fuel-to-air (FAR) ratio occurring at each load condition as indicated in Table S-1 above.


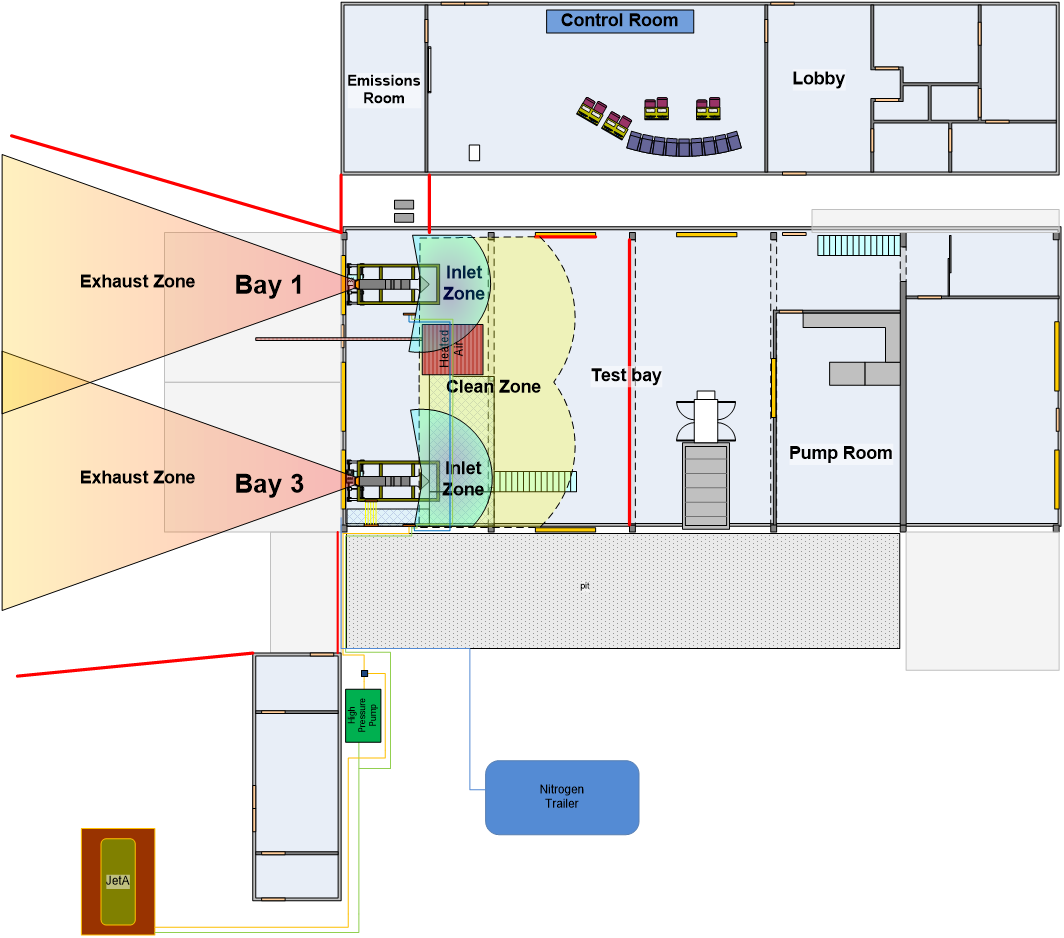


Test Equipment Location

J85 engine

Figure S-2. UTSI Propulsion Research Facility. All measurement equipment was in the Control Room.

Table S-3. Specifications for J85-GE-5 turbojet engine.

| **Characteristic or Operating Parameter** | **Specification** |
| --- | --- |
| Distance from turbine to exit plane | 181-186 cm |
| Exit plane nozzle diameter | Variable from 30 to 40 cm |
| Compressor | 8-stage axial |
| Combustors | Annular |
| Turbine | 2-stage |
| Thrust | 11 kN (16 kN w/afterburner) |
| Pressure ratio | 8.3 |
| Maximum air flow | 20 kg/s |
| Turbine inlet temperature | 977 °C |
| Specific fuel consumption | 27 g/kN-s |

Table S-4. Normalized thrust and normalized fuel flow versus J85 power lever angle (PLA) for non-afterburning engine operations from VARIAnT 4.

| **J85 Engine PLA**  **(degrees)** | **Estimated Percent Normalized Thrust (Non-Afterburning)** | **Jet-A Fuel Flow (kg/s)^a^** |
| --- | --- | --- |
| 15 | 3.8 | 0.048 |
| 20 | 3.9 | 0.064 |
| 30 | 9.1 | 0.096 |
| 40 | 23.5 | 0.128 |
| 50 | 54.3 | 0.160 |
| 60 | 74.4 | 0.192 |
| 70 | 82.7 | 0.224 |
| 75 | 84.5 | 0.240 |
| 80 | 92.4 | 0.256 |
| 90 | 100.0 | 0.288 |

**^a^** Taken from linear regression of PLA vs. fuel flow which includes data for all test points in the study.

A second DFCAS tested during VARIAnT 3 and 4, was the Libby Welding (Garrett now Honeywell) LGT-60 turbine powered start cart. Table S-5 provides specifications for this unit as published by the manufacturer.

Table S-5. General specifications for LGT-60 and GT-05 start carts.^a^

| **Characteristic or Operating Parameter** | **Specification** |
| --- | --- |
| Turbine model number | Garrett GTCP85-129(J) |
| Maximum rated rotor speed at full load | 41,000 revolutions/minute |
| Sea level shaft power | 64.9 kW |
| Bleed air flow | 47.6 kg/minute |
| Bleed air total pressure | 2540 mm Hg (absolute) |
| Maximum exhaust gas temperature | 691 degrees C |

^a^ Gas Turbine Auxiliary Power Unit, GAPD Model GTCP85-129(J), Document No. SC-5651M-5A, Allied Signal Aerospace, Garrett Auxiliary Power Division (now Honeywell), December 16, 1988.

During VARIAnT 3 an Isuzu 4LE2T diesel generator set was operated on hydrogenation derived renewable diesel (HDRD) fuel. Table S-6 provides specifications for the Isuzu 4LE2T diesel generator set system as operated by AEDC. Finally, during VARIAnT 4 test aerosols were also produced by Jing Model 5201 and 6200 Mini-CAST laboratory burners using propane as the fuel. Table S-1 provides the operating parameters for each burner.

Table S-6. Specifications of the Isuzu 4LE2T Diesel Generator Set

| **Characteristic or Operating Parameter** | **Specification** |
| --- | --- |
| Number of cylinders | 4 |
| Inlet air method | Turbocharged |
| Bore | 85 mm |
| Stroke | 96 mm |
| Total displacement | 2.2 L |
| Prime power output @ 1800 rpm | 30 kW |
| Maximum run time at 100% load | 28 hrs |
| Electrical output (maximum) | 25 kVA |

# J85 Sampling Probes

For the two VARIAnT campaigns, a small probe rake was fabricated by AEDC and mounted at the centerline of the J85 engine exit. The probe rake mounting included a remotely controlled positioning system which could move the probe rake horizontally along the entire engine exit plane. Figure S-3 shows a diagram of the VARIAnT 1 sampling probe system used in VARIAnT 3. Figure S- 4 illustrates the VARIAnT 4 rake system with a detailed picture of the 4 individual sampling probes.


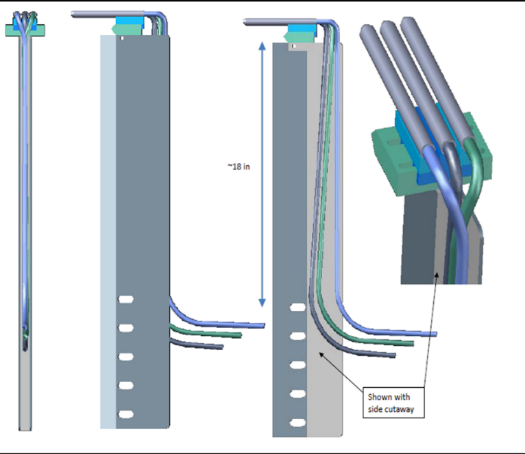

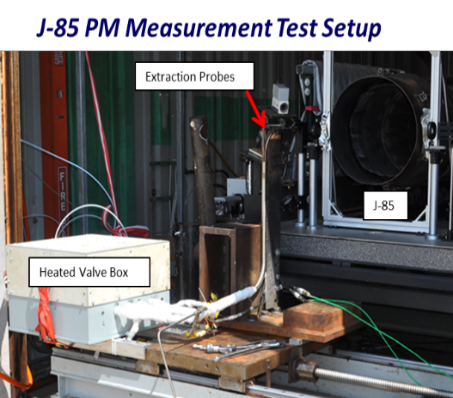


Figure S-3. Diagram of the VARIAnT 3 sampling probes (left) and photo of the VARIAnT 1 probes (also used in VARIAnT 3 test campaign) mounted behind the J-85 engine (right).


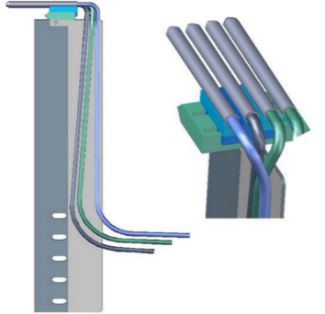

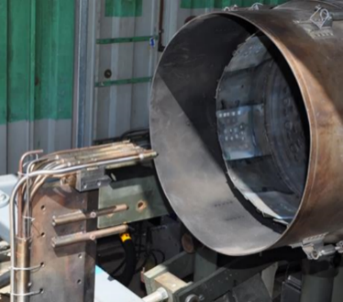

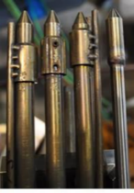


Figure S-4. Diagram of VARIAnT 4 probe system (left) with more detailed picture of the probes (center) and a picture of the probe system mounted behind the J85 engine (right).

# Sampling System Configurations

As described in the main text, a variety of sampling system configurations were used in both campaigns. A description of each instrument type is provided in Table 3 of the main text. Shown in Figure S-5, Figure S-6, Figure S-7, and Table S-7 are the sampling configurations used at NVFEL in VARIAnT 3, UTSI in VARIAnT 3 and VARIAnT 4, respectively.

# Sampling Plenum Design

As described in the main text, multi-port sampling plenums were used to distribute the aerosol sample to the various instruments. Geometric dimensioning of each plenum was aided by using a numerical methods software package (COMSOL Multiphysics^®^) to calculate gas flows and separate calculations of particle transport time and monodisperse particle coagulation assuming a monodisperse aerosol (e.g., Fuchs, 1989). The plenums used in the two campaigns differed in diameter, length, number of probes and total flow through the plenum. Due to the additional


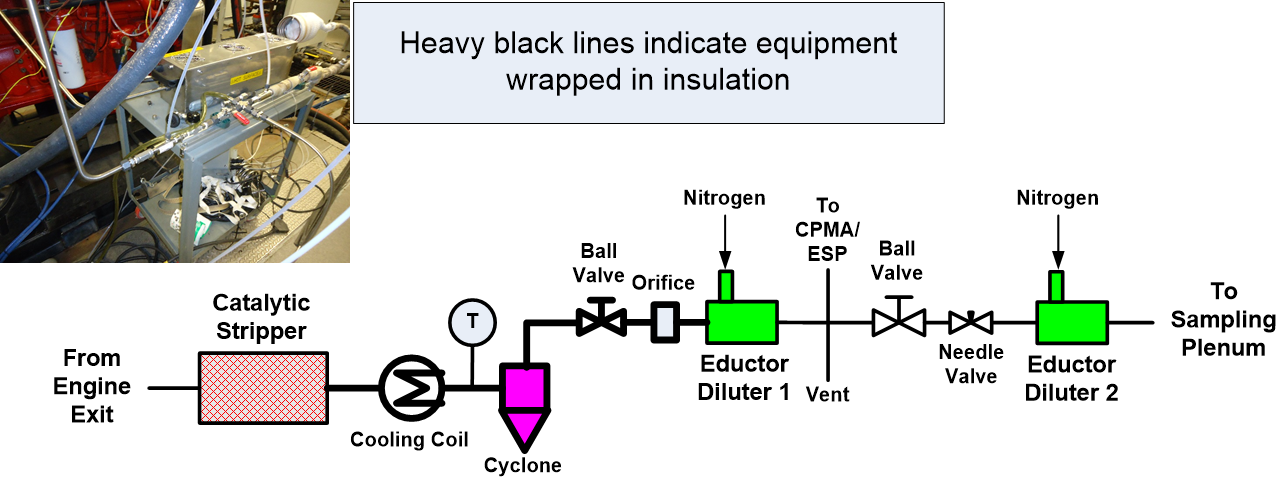


Figure S-5. The sampling system configuration used for testing at NVFEL in VARIAnT 3 (Cummins diesel).


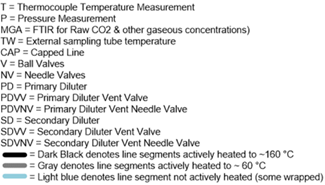
**
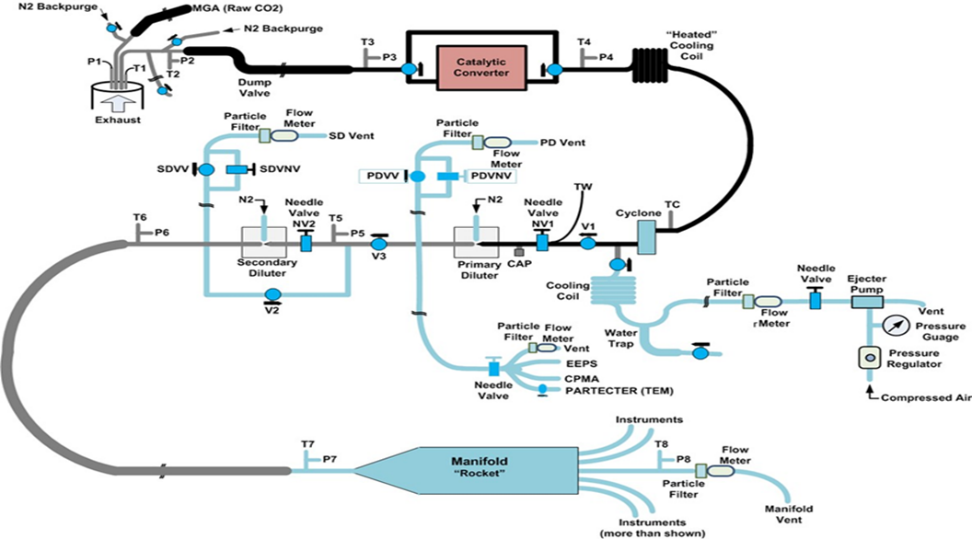
**

Figure S-6. Sampling system configuration for testing at UTSI during VARIAnT 3.


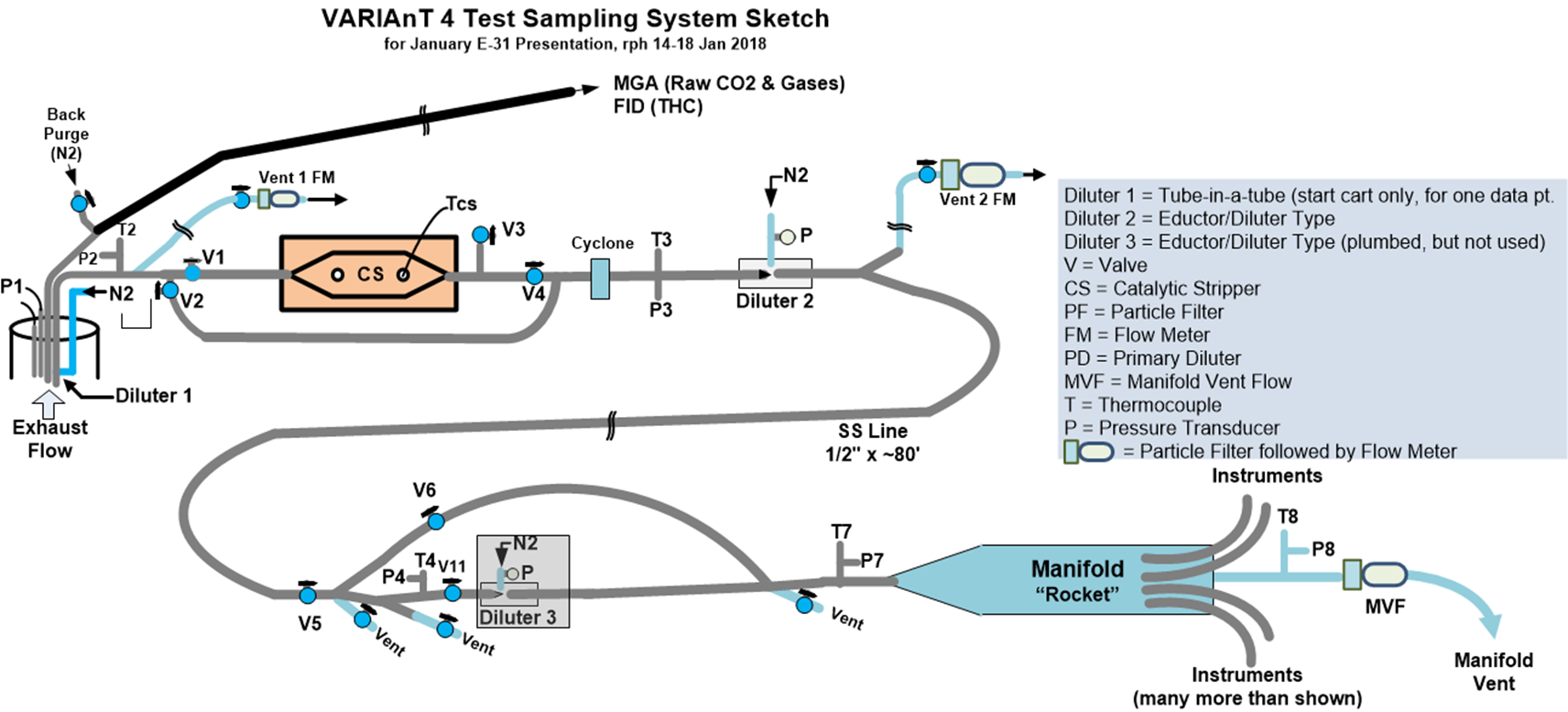


Figure S-7. Sampling system used in VARIAnT 4.

Table S-7. Nominal sample line lengths and diameters for Figure S-5, Figure S-6 and Figure S-7.

| Figure reference | Line segment | Sample line length  (m) | Sample line diameter  (mm) |
| --- | --- | --- | --- |
| Figure S-5 | Exhaust probe to catalytic stripper | 1 | 9.525 |
| Figure S-5 | Inlet to out of catalytic stripper | 0.53 |  |
| Figure S-5 | Catalytic stripper to first diluter | 0.81 | 9.525 |
| Figure S-5 | 1^st^ diluter to 2^nd^ diluter | 0.203 | 9.525 |
| Figure S-5 | 2^nd^ diluter to plenum | 2 | 9.525 |
| Figure S-5 | Plenum to instruments | 5 | 7.6 |
| Figure S-6 and S-7 | Probe tip to catalytic stripper | 2.03 | 9.525 |
| Figure S-6 and S-7 | Inlet to outlet of catalytic stripper | 0.53 |  |
| Figure S-6 and S-7 | catalytic stripper to first diluter | 0.81 | 9.525 |
| Figure S-6 and S-7 | 1st diluter to transport lines | 0.64 | 9.525 |
| Figure S-6 and S-7 | transport lines (2 lines in parallel) | 6.1 | 9.525 |
| Figure S-6 and S-7 | from transport lines to 2nd diluter | 0.76 | 9.525 |
| Figure S-6 and S-7 | from 2nd diluter to the plenum | 0.43 | 9.525 |

number of instruments used in VARIAnT 4, total flows were near 75 lpm or greater with VARIAnT 3 flows ranging from about 40 to 80 lpm. To assure that an identical sample was provided by each probe, preliminary measurements were performed prior to each campaign by connecting the same instrument sequentially from one port to another to assure that both the concentration and particle size distribution was the same at each port location. A plenum of the same basic design but with different dimensions and flowrates were provided for each study. Figure S-8 and Figure S- 9 show construction details for the plenum used in VARIAnT 3 and 4, respectively.

Figure S-8. VARIAnT 3 sampling plenum (a) detailed dimensions; and (b) probe assembly detail.


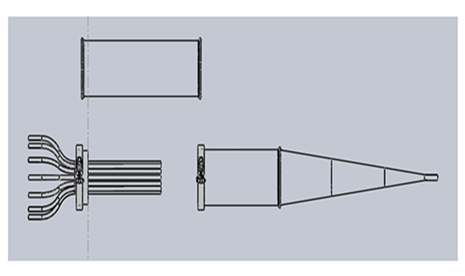

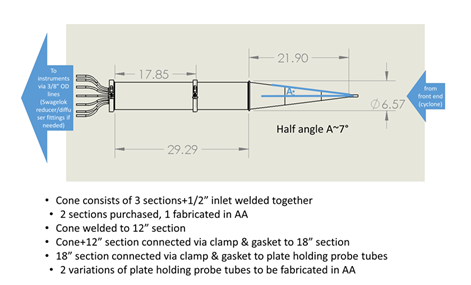


**(b)**

**(a)**


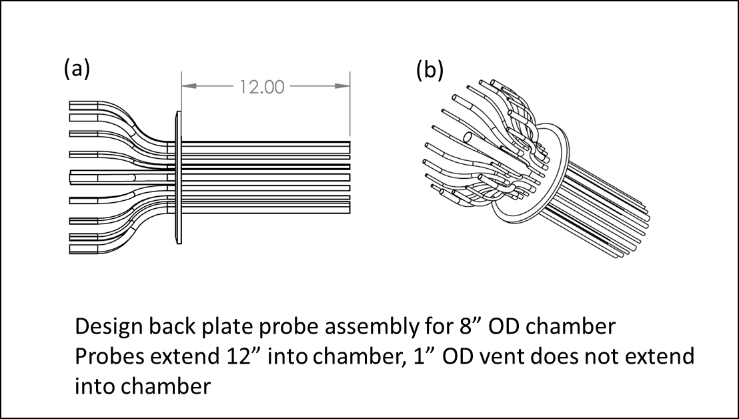


Figure S-9. VARIAnT 4 sampling plenum backplate design for the 8 inch diameter plenum showing (a) side view to highlight the depth of probes into the chamber; and (b) angled view to show the probes and 1 inch vent details. The VARIAnT 4 plenum used the same design strategies as the VARIAnT 3 plenum but for an 8 inch outer diameter.

# Instrument Calibrations and Quality Control Checks

Calibrations of the black carbon mass instruments were performed prior to each campaign according to the requirements specified in ARP6320A, including the optional test points at a target concentration of 50 µg/m^3^ (SAE, 2021). In these campaigns, however, the calibrations performed did not use a laboratory scale propane diffusion burner as was done during VARIAnT 1 and 2 due to the measured performance of the Artium LII-300 laser induced incandescence (LII) analyzer when this type of DFCAS was used. During VARIAnT 1 and 2, the readings from the LII-300 were up to 45 % lower than those produced by the AVL Micro Soot Sensor (MSS) photoacoustic instrument during testing of the J-85 (Kinsey et al., 2021). It was determined, therefore, that either a full gas turbine or turbine engine combustor rig was needed as the calibration source for the LII-300.

During VARIAnT 3, the calibration was conducted at Southwest Research Institute (SwRI) using a combustor sector rig as the aerosol source. In the case of VARIAnT 4, a Libby Welding Company (Garrett now Honeywell) LGT-60 turbine powered start cart located at Arnold Air Force Base, TN was used for the calibration. Both the combustor rig and start cart burned available Jet-A fuel. It should be noted during VARIAnT 3, however, that the CAPS PM_SSA_ instrument only received a partial calibration due to window contamination during early test points. This was not the case during VARIAnT 4 which included a complete qualification of the instrument. Details of both calibrations are provided below.

## VARIAnT 3 Calibration at Southwest Research Institute (SwRI)

Prior to VARIAnT 3, except for the MSS+ calibrated at AVL, all BC mass instruments were calibrated at SwRI using a 3-sector cup from a GE T700 combustor liner (Figure S-10) and the ARP6320 thermal-optical reference method ASTM D6877-13 for elemental carbon (EC) determination using a Sunset laboratory semicontinuous OCEC analyzer RT-3079 (calculation program version RTCalc631, instrument SBasic version 0, VBasic version RTOCEC62359). The mass concentrations reported by the BC mass instruments were compared to the elemental carbon mass collected on 25 mm diameter quartz filters at a sample flow rate of 5 lpm and a target filter mass loading of 12 ± 5 μg/cm^2^. A schematic of the sampling system used by SwRI is provided in Figure S-11. Stainless steel lines were used from the probe to each of the instruments. The lines were chosen so that the residence time in each line was approximately 1.25 seconds from the probe tip in the tunnel to each instrument inlet and the sample plenum was checked to ensure there was no sample bias amongst the instrument sample probe lines. Lastly, daily instrument checks were conducted for each instrument at the start of each day.

The combustor rig was run at a constant condition, simulating cruise, with a Jet A fuel flow rate of 63.5 kg/hour, inlet air pressure of 1655 kPa, and air temperature of 449 °C. The exhaust from the combustor rig was conditioned with a catalytic converter maintained at 300 °C to remove volatile and semi-volatile species and maintain an elemental to total carbon ratio of 0.8 or greater. Just downstream of the catalytic converter a cooling coil maintained to 60 °C was used to cool the exhaust sample. An ejector diluter was then used to dilute the exhaust sample with N_2_ to concentrations of 50, 100, 250, and 500 μg/cm^3^ and maintain a flow rate of 90 lpm into the plenum for each of these 4 concentrations. There were 6 data points taken at each of these concentration levels to determine the calibration factors listed in Table S-8. Additionally, 6 runs with clean N_2_ at a dilution ratio of 6:1 were conducted to determine a zero concentration point. The combustor soot size distribution delivered to the instruments at the 50 μg/m^3^ test point was measured with an EEPS over a 10 minute test interval at the bypass of the plenum. The measured size distribution had a geometric mean of 41.98 nm, a mode of 45.32 nm, concentration peak of 7.8 x 10^5^ particles/cm^3^ and geometric standard deviation of 1.64.


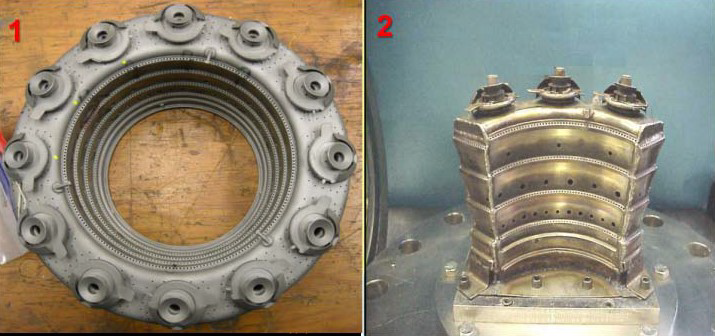


Figure S-10. GE T700 combustor liner


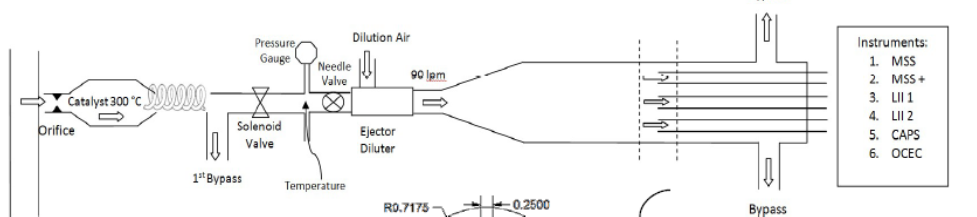


Figure S-11. SwRI calibration sampling configuration and plenum. MSS = AVL Micro Soot Sensor; MSS + = AVL Micro Soot Sensor Plus; LII = Artium LII-300; CAPS = Aerodyne CAPS PM_SSA_; and OCEC = Sunset Model RT-3079

**Table S-8.** Results of VARIAnT 3 calibrations

| Target EC mass conc. (μg/m^3^) |  | | Date | Test # | Test duration (minutes) | Filter loading, EC (μg/cm^2^) | EC/ (EC+OC) | Mass concentrations at 0 °C and 101.325 kPa^a^ | | | | | |
| --- | --- | --- | --- | --- | --- | --- | --- | --- | --- | --- | --- | --- | --- |
|  |  | |  |  |  |  |  | Thermal EC (μg/m^3^) | LII-300  (SN 477) (μg/m^3^) | LII-300  (SN 369) (μg/m^3^) | MSS  (SN 1293) (μg/m^3^)^b^ | MSSplus (SN 2246) (μg/m^3^) | CAPS PM_SSA_ (μg/m^3^)^c^ |
| 50 |  | | 2016-12-08 | 1 | 120 | 11.1 | 0.86 | 51.1 | 84.3 | 112.6 | 54.7 | 50.9 | 62.0 |
|  |  | | 2016-12-09 | 2 | 120 | 10.3 | 0.83 | 47.2 | 87.2 | 117.6 | 54.1 | 50.9 | 63.0 |
|  |  | | 2016-12-09 | 3 | 120 | 10.8 | 0.84 | 49.7 | 84.3 | 111.1 | 53.9 | 50.9 | 58.7 |
|  |  | | 2016-12-09 | 4 | 120 | 11.3 | 0.86 | 51.9 | 87.6 | 115.7 | 56.2 | 52.2 | 65.2 |
|  |  | | 2016-12-09 | 5 | 120 | 11.7 | 0.86 | 53.8 | 85.8 | 111.9 | 56.4 | 52.6 | 65.3 |
|  |  | | 2016-12-10 | 6 | 120 | 11.1 | 0.86 | 51.4 | 89.4 | 116.5 | 56.6 | 53.0 | 63.6 |
| 100 |  | | 2016-12-06 | 1 | 60 | 13.0 | 0.96 | 121.8 | 182.6 | 300.4 | 120.4 | 111.2 | 135.9 |
|  |  | | 2016-12-06 | 2 | 60 | 9.7 | 0.86 | 91.3 | 152.2 | 209.6 | 103.2 | 95.2 | 116.3 |
|  |  | | 2016-12-07 | 3 | 60 | 12.2 | 0.87 | 114.1 | 186.4 | 257.2 | 119.7 | 109.3 | 135.2 |
|  |  | | 2016-12-07 | 4 | 60 | 12.5 | 0.85 | 118.2 | 189.5 | 259.9 |  | 118.7 | 145.6 |
|  |  | | 2016-12-07 | 5 | 60 | 10.9 | 0.84 | 103.1 | 163.7 | 223.7 | 114.2 | 104.6 | 127.5 |
|  |  | | 2016-12-07 | 6 | 60 | 11.4 | 0.84 | 108.0 | 162.9 | 223.1 | 114.7 | 105.3 | 128.3 |
|  |  | | 2016-12-08 | 7 | 60 | 10.6 | 0.83 | 97.8 | 164.1 | 222.3 | 106.8 | 99.0 | 123.5 |
| 250 |  | | 2016-12-03 | 1 | 25 | 12.6 | 0.88 | 287.0 | 424.1 | 726.9 | 288.8 | 264.8 |  |
|  |  | | 2016-12-03 | 2 | 25 | 12.4 | 0.88 | 283.7 | 456.1 | 804.7 | 314.4 | 289.5 |  |
|  |  | | 2016-12-03 | 3 | 25 | 12.5 | 0.91 | 284.9 | 432.2 | 736.9 | 294.1 | 270.5 |  |
|  |  | | 2016-12-03 | 4 | 25 | 11.2 | 0.91 | 254.4 | 398.5 | 662.8 | 268.4 | 246.7 |  |
|  |  | | 2016-12-03 | 5 | 25 | 12.4 | 0.91 | 281.2 | 449.4 | 742.5 | 299.4 | 275.1 |  |
|  |  | | 2016-12-03 | 6 | 25 | 12.4 | 0.92 | 281.2 | 449.9 | 747.6 | 300.6 | 276.5 |  |
| 500 |  | | 2016-12-02 | 1 | 13 | 11.6 | 0.87 | 502.7 | 812.5 | 1204.4 | 554.7 | 513.0 | 768.7 |
|  |  | | 2016-12-02 | 2 | 13 | 11.6 | 0.88 | 509.6 | 787.4 | 1129.4 | 539.1 | 499.4 | 656.5 |
|  |  | | 2016-12-02 | 3 | 13 | 11.8 | 0.89 | 515.6 | 825.7 | 1186.4 | 563.5 | 523.3 | 606.8 |
|  |  | | 2016-12-02 | 4 | 13 | 12.8 | 0.91 | 563.7 | 872.6 | 1269.3 | 597.6 | 555.6 | 538.0 |
|  |  | | 2016-12-02 | 5 | 13 | 11.1 | 0.91 | 488.4 | 764.5 | 1095.7 | 523.4 | 485.0 | 475.3 |
|  |  | | 2016-12-02 | 6 | 13 | 11.4 | 0.92 | 503.7 | 789.9 | 1135.9 | 544.0 | 504.1 | 483.0 |
|  | | Calibration constants | | | | | | | 0.63 | 0.42 | 0.93 | 1.00 | 0.83 |
|  | | one standard deviation of calibration constant averages | | | | | | | 0.03 | 0.03 | 0.03 | 0.04 | 0.10 |

^a^ The LII-300s, MSS and MSSplus have their serial numbers (SN) listed in parenthesis.

^b^ MSS (SN 1293) data was not recorded at 100 μg/m^3^ during test #4.

^c^ CAPS PM_SSA_ data at 500 μg/m^3^ not used and at 250 μg/m^3^ not available due to dirty optics and cleaning. LII-300 (SN 369) data at 250 μg/m^3^ were taken when the LII-300 (SN 369) was in the single channel mode.

## VARIAnT 4 Mass Instrument Calibrations

The mass instrument calibrations were performed prior to the VARIAnT 4 test campaign on July 19-22, 2018 at the U. S. Air Force, Arnold Engineering Development Complex in collaboration with the U. S. Environmental Protection Agency, National Risk Management Research Laboratory. The calibrations were conducted in compliance with the protocols outlined in SAE ARP6320 and by the International Civil Aviation Organization (ICAO) in ANNEX 16 Volume II, Appendix 7, Attachment B. The following provides details of the work conducted and the results obtained.

A Libby Welding Company LGT-60 turbine engine start cart (see Table S-5) burning military grade Jet-A was used as the nvPM source for the calibration. The sampling system is illustrated in Figure S-12. The nvPM gas sample entered a single probe sampling system and was immediately diluted to about 4:1 (dilution factor) using dry nitrogen. The diluted sample passed through a catalytic stripper, a BGI Scc 2.354 cyclone (1 µm cut-point at 17.8 lpm), a second dilution stage, a long sample line and into a sample plenum where it was distributed among several instruments. The plenum had a conic inlet attached to a cylinder with multiple outlets sized for the differing flow requirements of the instruments (Figure S-~~9~~8). Pre-calibration testing of the sampling plenum for particle mass, number and size was conducted to assure that all instruments received the identical sample aerosol regardless of position on the sampling plenum.

**
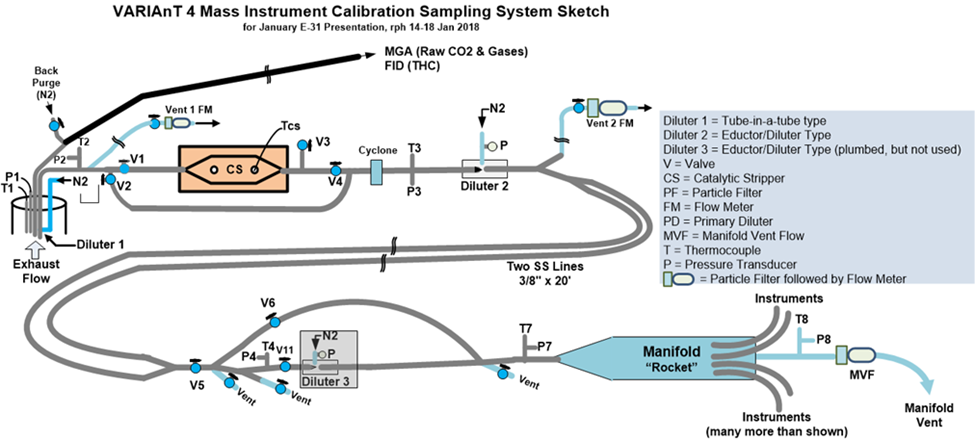
**

Figure S-12. Sampling system for mass instrument calibrations in VARIAnT 4.

The start cart was operated with no load and fueled with Jet-A meeting ICAO specifications from ANNEX 16 Volume II, Appendix 4. The nvPM mass concentration supplied to the instruments was varied primarily by diluting the sample with an ejector diluter at the second dilution stage, and as necessary, small adjustments to the Diluter 1. The nvPM mass concentration (more strictly, elemental carbon concentration) was quantified using two different OCEC analyzer types produced by Sunset Laboratory, Incorporated: a Model-4 Semi-Continuous OCEC Field Analyzer and a Model 4L Laboratory OCEC Aerosol Analyzer. The raw data collected from these instruments were analyzed to determine the elemental carbon (EC) and organic carbon (OC) mass concentrations using the Sunset laboratory analysis programs listed in Table S-9. Since the laboratory OCEC analyzer is considered more accurate, the EC concentrations as determined from measurements by this instrument were used to calculate the calibration coefficients for the mass instruments. The semi-continuous OCEC Field Analyzer measurements agreed within uncertainty of the measurements and, thus, corroborated the Laboratory OCEC results.

Table S-9. Primary equipment list used in the VARIAnT 4 mass instrument calibration.

| **Equipment description** | **Model number** | **Instrument software** | | **Data analysis software** |
| --- | --- | --- | --- | --- |
| Sunset Laboratory, Inc. Semi-continuous OCEC instrument | 4 | SBasic,  version 17300 | VBasic,  Version RTOCEC62361 | RTCalc661 |
| Sunset Laboratory, Inc. Laboratory OCEC instrument | 4L | SBasic,  Version 206 | VBasic,  Version OCEC828 | Calc319 |
| Libby Welding Company (Garrett) turbine engine start cart | LGT-60 | - | - | - |
| AIR-VAC TDSS vacuum pump (ejector diluter)^a^ | TD260HSS  TD190HSS  TD110HSS | - | - | - |
| BGI cyclone,  D50=2.5μm at 8 lpm | Scc 2.354 | - | - | - |
| Catalytic instruments catalytic stripper | CS15, 15 lpm | - | - | - |

^a^https://airvacpumps.com/TDstainless.html

Nitrogen was used to dilute the sample to target concentrations of 50, 100, 250, and 500 µg/m^3^. Table S-10 lists the concentrations as measured by the CAPS PM_SSA_, MSSs, LII-300s, and laboratory OCEC analyzer. The table includes values of the percent of elemental carbon mass concentration relative to the total carbon mass measured using the OCEC analyzer, % EC. Note that the manual EC mass concentrations have been blank corrected and the manual OC mass concentrations have been both blank corrected and corrected for gas phase artefacts in the same manner as discussed in the main text for the VARIAnT 4 campaign.

After the VARIAnT 4 campaign was complete, it was determined by Artium that the NASA LII had issues with its laser. For this reason, we do not report any results for the NASA LII.

During the VARIAnT 4 calibration, the online semicontinuous OCEC analyzer was run in addition to collecting filters for offline manual EC analysis. The data from both EC analysis methods are shown in Table S-11 for each of the calibration test points. Note that data from the semicontinuous instrument was scaled from a reference temperature of 20°C to 0°C. The data in Table S-11 is shown with the data corrected to 0°C for both the semicontinuous and manual EC analysis methods. The comparison between the two methods is shown in Figure S-13, where the blue line with a slope of 1.07 represents the fit and the dashed line is a line with a slope of 1 and intercept 0. Additional fit statistics for Figure S-13 are shown in Table S-12.

Table S-10. Results of VARIAnT 4 instrument calibration^a^.

| **Target Conc.** | **Date** | **Test Point** | **Manual EC (µg/m^3^)** | **% EC** | **CAPS PM_SSA_ (Aerodyne) (µg/m^3^)** | **LII-300 (AEDC) (µg/m^3^)** | **LII-300 (HW) (µg/m^3^)** | **LII-300 (Artium) (µg/m^3^)** | **LII-300 (NASA) (µg/m^3^)** | **MSS (AEDC,** **S/N1293 ) (µg/m^3^)** | **MSSplus (HW) (µg/m^3^)** |
| --- | --- | --- | --- | --- | --- | --- | --- | --- | --- | --- | --- |
| 50 µg/m^3^ | 7/19/2018 | CT2-1 | 44.00 | 90 | 47.13 | 73.30 | 56.77 | Data Lost | 55.18 | 43.67 | 45.06 |
|  | 7/20/2018 | CT2-2b | 46.18 | 90 | 50.70 | 82.88 | 61.82 | 41.37 | 61.79 | 48.18 | 48.83 |
|  | 7/20/2018 | CT2-3 | 60.11^b^ | 98 | 57.67 | 91.95 | 70.07 | 47.20 | 68.80 | 54.01 | 55.55 |
|  | 7/20/2018 | CT2-4 | 46.07 | 89 | 50.22 | 79.92 | 61.52 | 40.78 | 59.07 | 46.97 | 48.36 |
|  | 7/20/2018 | CT2-5 | 45.58 | 92 | 49.85 | 78.75 | N/A | 40.91 | 57.55 | 46.21 | 47.47 |
|  | 7/21/2018 | CT2-6a | 48.25 | 86 | 50.94 | 86.57 | 62.10 | 36.94 | 61.58 | 47.88 | 49.02 |
|  | 7/22/2018 | CT2-7 | 46.17 | 91 | 51.44 | 82.07 | 63.32 | 38.99 | 60.24 | 46.62 | 47.99 |
| 100 µg/m^3^ | 7/18/2018 | CT3-1 | 91.06 | 84 | 95.81 | 150.40 | 112.49 | 83.12 | 115.60 | 89.97 | 93.05 |
|  | 7/18/2018 | CT3-2 | 95.83 | 94 | 95.75 | 150.57 | 113.86 | 83.62 | 115.39 | 90.08 | 92.78 |
|  | 7/18/2018 | CT3-3 | 86.98 | 92 | 97.37 | 153.10 | 116.14 | 85.13 | 117.51 | 91.35 | 94.25 |
|  | 7/19/2018 | CT3-4 | 96.22 | 88 | 102.52 | 163.76 | 119.87 | Void | 123.56 | 94.89 | 96.60 |
|  | 7/19/2018 | CT3-5 | 90.76 | 87 | 100.15 | 156.01 | 117.61 | Void | 117.77 | 93.25 | 94.60 |
|  | 7/19/2018 | CT3-6 | 90.81 | 83 | 98.65 | 152.20 | 116.35 | Void | 115.06 | 92.21 | 93.30 |
| 250 µg/m^3^ | 7/21/2018 | CT4-1 | 236.37 | 89 | 249.20 | 437.03 | 315.07 | 217.19 | 302.27 | 245.33 | 251.04 |
|  | 7/21/2018 | CT4-2 | 233.86 | 90 | 248.24 | 435.15 | 314.58 | 215.75 | 312.18 | 243.19 | 248.69 |
|  | 7/21/2018 | CT4-3 | 249.76 | 98 | 255.52 | 445.11 | 319.99 | 220.70 | 315.93 | 249.69 | 255.50 |
|  | 7/21/2018 | CT4-4 | 234.92 | 91 | 245.81 | 425.46 | 315.02 | 213.48 | 296.68 | 238.96 | 244.59 |
|  | 7/21/2018 | CT4-5 | 241.85 | 89 | 257.22 | 444.48 | 331.32 | 223.65 | 303.75 | 250.22 | 256.23 |
|  | 7/21/2018 | CT4-6 | 243.72 | 91 | 261.62 | 449.96 | 337.84 | 227.05 | 309.81 | 254.66 | 260.88 |
| 500 µg/m^3^ | 7/22/2018 | CT5-1 | 463.03 | 99 | 461.84 | 872.28 | 637.17 | 467.82 | 709.04 | 477.51 | 491.81 |
|  | 7/22/2018 | CT5-2 | 458.66^c^ | 94 | 467.67 | 889.29 | 649.31 | 457.24 | 688.69 | 482.41 | 496.36 |
|  | 7/22/2018 | CT5-3 | 451.77 | 95 | 469.26 | 866.97 | 652.07 | 457.12 | 652.51 | 481.49 | 496.22 |
|  | 7/22/2018 | CT5-4 | 447.36 | 95 | 462.00 | 858.42 | 638.32 | 458.89 | 677.36 | 473.84 | 488.16 |
|  | 7/22/2018 | CT5-5 | 501.27 | 100 | 462.71 | 868.30 | 640.50 | 459.75 | 688.12 | 474.19 | 489.14 |
|  | 7/22/2018 | CT5-6 | 499.52 | 100 | 455.28 | 845.92 | 629.87 | 444.00 | 660.80 | 464.94 | 479.19 |
| 0 µg/m^3^ |  |  | 0 | NA | 0 | 0 | 0 | 0 | 0 | 0 | 0 |
|  |  |  | 0 | NA | 0 | 0 | 0 | 0 | 0 | 0 | 0 |
|  |  |  | 0 | NA | 0 | 0 | 0 | 0 | 0 | 0 | 0 |
| **Calibration constant** | | | | | **1.074** | **0.587** | **0.794** | **1.122** | **0.764** | **1.061** | **1.032** |
| **one standard deviation of calibration constant averages** | | | | | **0.05** | **0.03** | **0.04** | **0.08** | **0.04** | **0.04** | **0.03** |

^a^ ARP6320 annual calibration requirements: 3 runs minimum; > 80% EC generated; actual EC concentration + 20% target; and EC filter loading 12 + 5 µg/m^2^.

^b^ Outside target concentration + 20%.

^c^ Filter loading above 12 + 5 µg/m^2^.


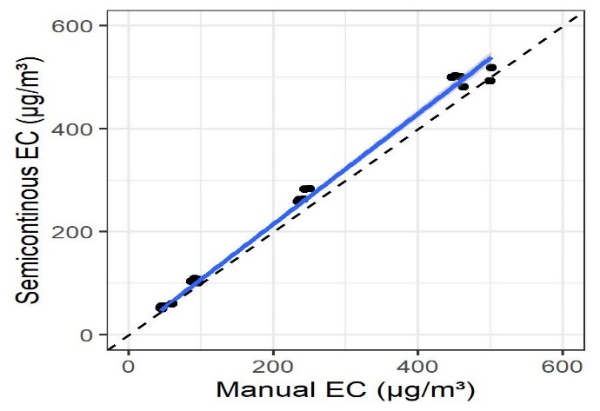


Figure S-13. Comparison of VARIAnT 4 manual EC to semicontinuous EC measurements taken during mass instrument calibration.

Table S-11. VARIAnT 4 mass instrument calibration manual to semicontinuous EC comparison.

| **Target Conc.** | **Date** | **Test Point** | **Manual EC (µg/m^3^)** | **Semicontinuous EC (µg/m^3^)** |
| --- | --- | --- | --- | --- |
| 50 µg/m^3^ | 7/19/2018 | CT2-1 | 44 | 52.3 |
|  | 7/20/2018 | CT2-2b | 46.2 | 50.4 |
|  | 7/20/2018 | CT2-3 | 60.1 | 60.1 |
|  | 7/20/2018 | CT2-4 | 46.1 | 51.4 |
|  | 7/20/2018 | CT2-5 | 45.6 | 55.2 |
|  | 7/21/2018 | CT2-6b | 48.3 | 55.9 |
|  | 7/22/2018 | CT2-7 | 46.2 | 55.3 |
| 100 µg/m^3^ | 7/18/2018 | CT3-1 | 91.1 | 109 |
|  | 7/18/2018 | CT3-2 | 95.8 | 101 |
|  | 7/18/2018 | CT3-3 | 87 | 104 |
|  | 7/19/2018 | CT3-4 | 96.2 | 107 |
|  | 7/19/2018 | CT3-5 | 90.8 | 104 |
|  | 7/19/2018 | CT3-6 | 90.8 | 106 |
| 250 µg/m^3^ | 7/21/2018 | CT4-1 | 236 | 262 |
|  | 7/21/2018 | CT4-2 | 234 | 259 |
|  | 7/21/2018 | CT4-3 | 250 | 283 |
|  | 7/21/2018 | CT4-4 | 235 | 260 |
|  | 7/21/2018 | CT4-5 | 242 | 263 |
|  | 7/21/2018 | CT4-6 | 244 | 282 |
| 500 µg/m^3^ | 7/22/2018 | CT5-1 | 463 | 481 |
|  | 7/22/2018 | CT5-2 | 459 | 501 |
|  | 7/22/2018 | CT5-3 | 452 | 503 |
|  | 7/22/2018 | CT5-4 | 447 | 500 |
|  | 7/22/2018 | CT5-5 | 501 | 519 |
|  | 7/22/2018 | CT5-6 | 500 | 493 |

Table S-12. Fit statistics for comparison of VARIAnT 4 mass instrument calibration comparing Manual EC to Semi-Continuous EC.

| **Slope** | **Slope Standard Error** | **R-Squared** | **Degrees of freedom** |
| --- | --- | --- | --- |
| 1.07 | 0.01 | 1.00 | 24 |

## OCEC Analyzer Calibration

Prior to the start of each experimental program, multi-point calibrations were performed for the Sunset Model 4 semi-continuous OCEC analyzer (Sunset Laboratory, Inc., n.d.) using a standard sucrose solution (C_12_H_22_O_11_ plus distilled water). Sucrose checks and method blanks were also conducted daily during testing for quality control purposes. For the laboratory OCEC analyzer (Sunset Laboratory, Inc., n.d.), calibration checks of the instrument were conducted each day. These checks consisted of multi-point tests using a range of sucrose loadings, as well as running filter blanks. Both types of OCEC analyzers used the same operating parameter file (NIOSH930.par) supplied by Sunset Laboratories.

For the manual determination of EC, prior to each campaign, all 25-mm quartz filters were pre-baked at a temperature of 550 °C for at least 12 hours and stored in a laboratory freezer at a nominal temperature of – 50 °C. All filters were stored in a portable freezer at a nominal temperature of – 20 °C prior to sampling and analysis including transport to the test site in the EPA vehicle.

## Quality Control Checks

In addition to the calibration of the mass and OCEC instruments, pre-test evaluations of the sizing instruments and other key measurement equipment were also performed. For the SMPSs, EEPS, and DMS 500 these checks consisted of challenging each analyzer using a reference oil aerosol from the atomization of ~ 10 ppm dioctyl sebacate (DOS) in high purity isopropyl alcohol. Sizing accuracy was also checked periodically using certified standard polystyrene latex (PSL) particles. Other checks included monitoring upstream and downstream CO_2_ concentrations for dilution factor determination and ensuring excess flow through the plenum dump.

Finally, prior to each day’s testing, additional quality control checks were performed. This included completion of a detailed operating parameter checklist for each mass analyzer to verify proper operation of each instrument, sampling media logs to identify the filters used for determination of OCEC and total PM mass installed in the multi-filter samplers, and leak checks of these samplers to assure reliable operation. In addition, method blank and field blank quartz and Teflon filters were collected for quality assurance purposes.

Daily zero checks were performed to ensure no shedding or leaks issues occurred in the sampling system. During these zero checks, MSS mass concentrations and APC number concentrations were typically 0.5 μg/m^3^ and < 100 particles/cm^3^, respectively. Additionally, a post check/analysis of measured size and number concentrations confirmed there were no measurable particle size distributions or number concentrations when no source was in operation throughout the entire test campaign.

## Daily Consistency Checks

Figure 7 in the main text shows significant test-to-test variation at a given fuel mass flow rate, especially for VARIAnT 4. We believe this is due mainly to day-to-day variation in the J‑85 operation at the same nominal test condition. Figure S-14, Figure S-15, and Figure S-16 are plots of apparent density based on the ratio of AEDC MSS black carbon mass concentration to SMPS 3938 (NVFEL) volume concentration for the J-85 operating with Jet-A fuel in VARIAnT 4 plotted against fuel mass flow rate, MSS concentration and geometric mean mobility diameter, GMD, respectively.

For each test series there is a distinct pattern with most cases slightly decreasing apparent density with fuel flow (load), MSS concentration (function of load and dilution factor), and geometric mean diameter. There is somewhat more scatter with test series T4. For all but one case (T1), there is an inverse relationship between apparent density and geometric mean diameter, consistent with larger more open structures as size increases.


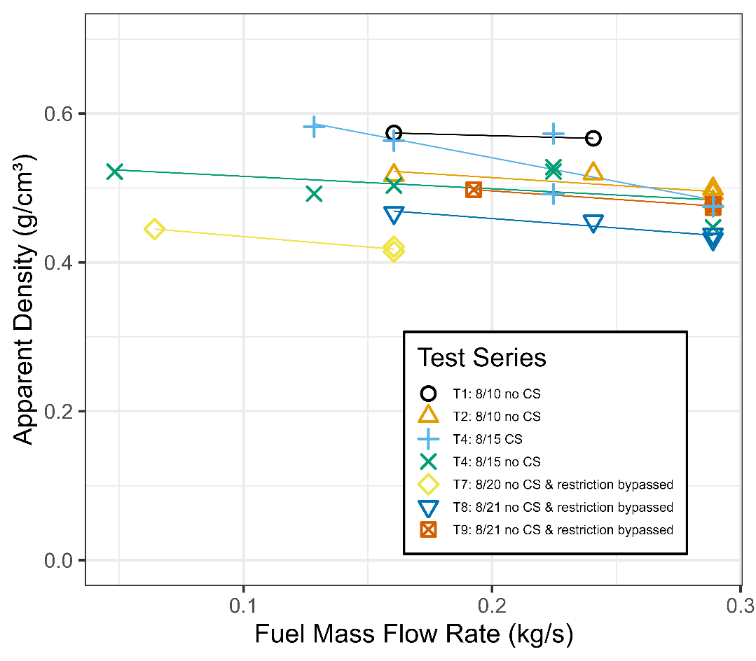


Figure S-14. Apparent density as a function of fuel mass flow rate for different test series. Apparent density is calculated from taking the MSS (AEDC) black carbon mass concentration divided by the SMPS 3938 (NVFEL) volume concentration plotted against fuel mass flow rate. All points are for the J-85, with Jet-A fuel in VARIAnT 4. Note that the lines are drawn only to guide the readers eyes and to group the data by test series.


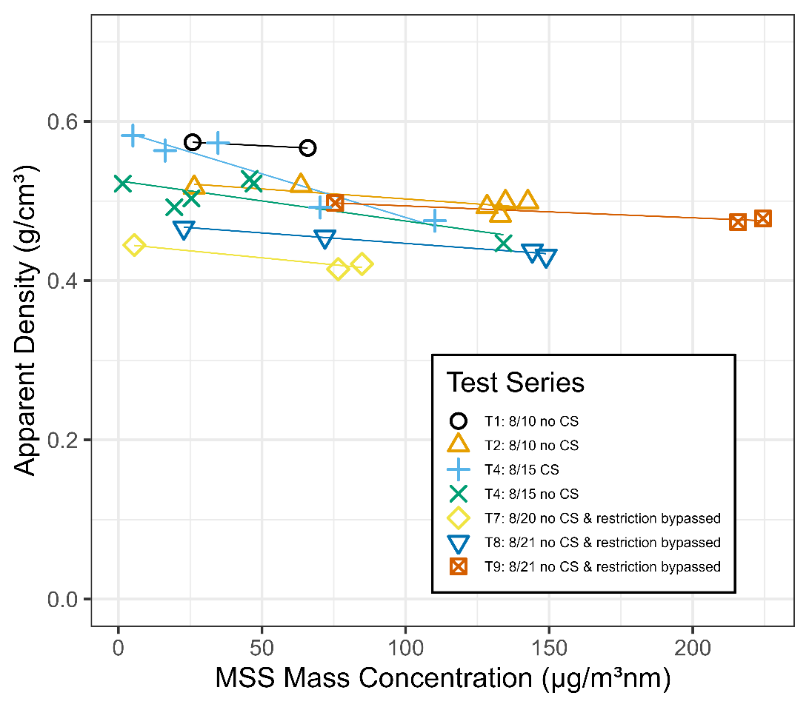


Figure S-15. Apparent density as a function of MSS mass concentration for different test series. Apparent density is calculated from taking the MSS (AEDC) black carbon mass concentration divided by the SMPS 3938 (NVFEL) volume concentration plotted against MSS BC mass concentration. All points are for the J-85, with Jet-A fuel in VARIAnT 4. The lines are drawn only to guide the readers eyes and to group the data by test series.


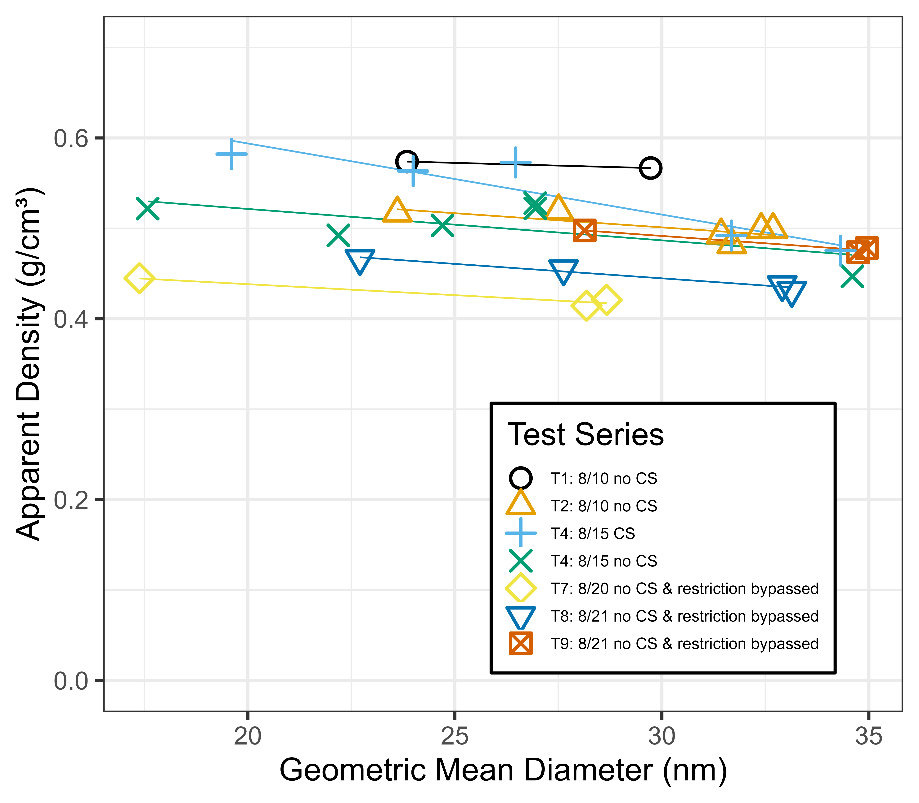


Figure S-16. Apparent density as a function of geometric mean diameter (GMD) for different test series. Apparent density is calculated from taking the MSS (AEDC) black carbon mass concentration divided by the SMPS 3938 (NVFEL) volume concentration plotted against SMPS geometric mean mobility diameter. All points are for the J-85, with Jet-A fuel in VARIAnT 4. The lines are drawn only to guide the readers eyes and to group the data by test series.

Another indicator of inconsistent day-to-day operation is the CO_2_ concentration. Figure S-17 and Figure S-18 are plots of CO_2_ overall and CO_2_ by test series, respectively, against fuel mass flow rate. There is clearly significant day-to-day variation.

The purpose of this work was not to characterize DFCASs themselves but rather to use them for instrument comparisons. Based on the discussion above, we suggest that much of the variability in instrument comparisons results from source variability, especially in the case of the J-85, rather than instrument variability.


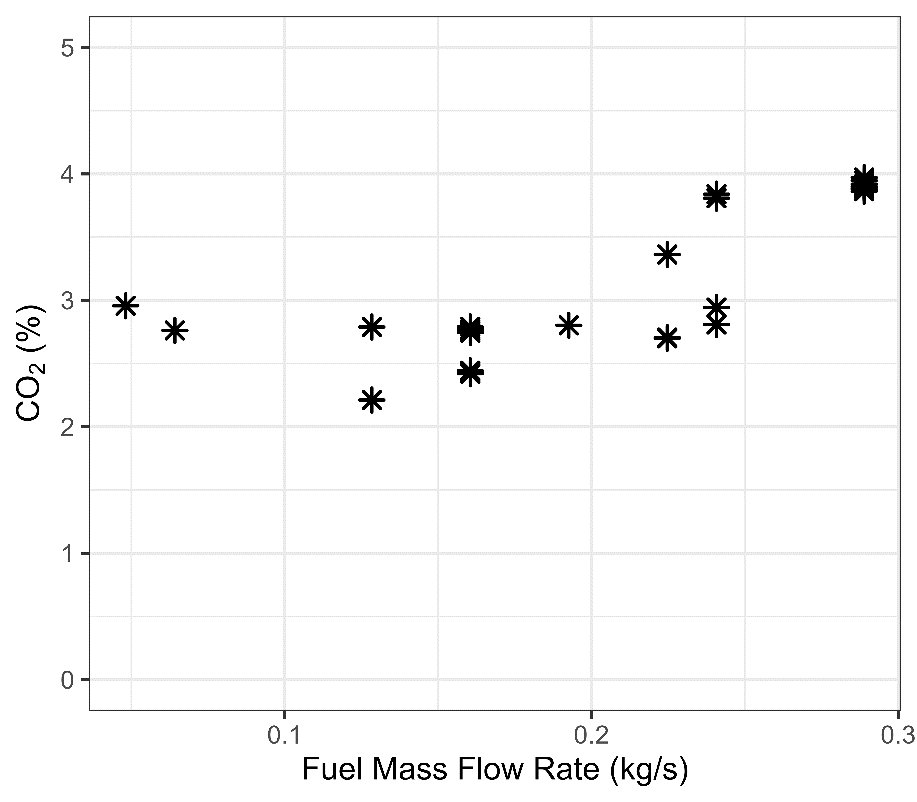


Figure S-17. Exhaust CO_2_ concentration plotted against fuel mass flow rate for the J-85 burning Jet-A fuel during VARIAnT 4.


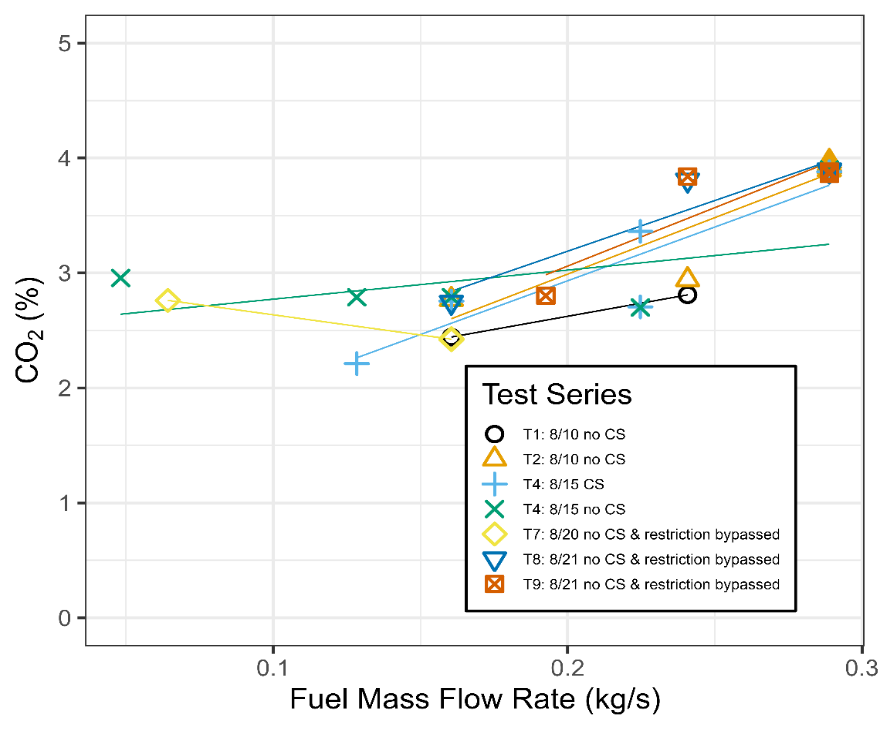


Figure S-18. Exhaust CO_2_ concentration plotted against fuel mass flow rate by test series for the J85 burning Jet-A fuel during VARIAnT 4. The lines are drawn only to guide the readers eyes.

# Regression Statistics

A number of linear regressions were performed on the data as discussed in the main text. These include the instrument comparisons in Figures 2 and 3, the comparison of sizing instrument performance in Figure 5, and measurement of carbonaceous particles versus total PM in Figure 6 of the main text. Each line that was fit was a linear regression with a fixed y-intercept of 0 and the reported statistics are the slope of the line, the slope standard error, the r-squared value and degrees of freedom which are provided in Table S-13 for Figure 2, Table S-14 for Figure 3, Figure S-15 for Figure 5, and Table S-16 for Figure 6. Table S-16 provides another method to examine the x and y values relative to one another: average and standard deviation of the ratio of y over x for all points in each plot panel.

Table S-13. Results of VARIAnT 3 instrument comparison to the EC reference, statistics of linear regressions from Figure 2.

| **Aerosol Source** | **Instrument** | **Slope** | **Slope Standard Error** | **R-Squared** | **Degrees of Freedom** |
| --- | --- | --- | --- | --- | --- |
| Cummins | CAPS PM_SSA_ (Aerodyne) | 0.91 | 0.04 | 0.96 | 24 |
|  | LII-300 (AEDC) | 1.08 | 0.01 | 1.00 | 24 |
|  | LII-300 (Artium) | 1.01 | 0.02 | 0.99 | 24 |
|  | MSS (AEDC) | 1.05 | 0.01 | 1.00 | 24 |
|  | MSS plus (AVL) | 1.05 | 0.01 | 1.00 | 24 |
| Isuzu Diesel Gen Set | CAPS PM_SSA_ (Aerodyne) | 0.90 | 0.02 | 1.00 | 3 |
|  | LII-300 (AEDC) | 0.94 | 0.02 | 1.00 | 3 |
|  | LII-300 (Artium) | 0.94 | 0.02 | 1.00 | 3 |
|  | MSS (AEDC) | 0.92 | 0.01 | 1.00 | 3 |
|  | MSS plus (AVL) | 0.95 | 0.01 | 1.00 | 3 |
|  | MSS plus (AVL) [AVL Cal.] | 1.01 | 0.01 | 1.00 | 3 |
| GT-05 | CAPS PM_SSA_ (Aerodyne) | 0.87 | 0.01 | 1.00 | 3 |
|  | LII-300 (AEDC) | 1.02 | 0.02 | 1.00 | 3 |
|  | LII-300 (Artium) | 1.05 | 0.01 | 1.00 | 3 |
|  | MSS (AEDC) | 0.94 | 0.01 | 1.00 | 3 |
|  | MSS plus (AVL) | 0.97 | 0.01 | 1.00 | 3 |
|  | MSS plus (AVL) [AVL Cal.] | 1.05 | 0.01 | 1.00 | 3 |
| LGT-60 | CAPS PM_SSA_ (Aerodyne) | 0.88 | 0.01 | 0.99 | 19 |
|  | LII-300 (AEDC) | 0.90 | 0.02 | 0.99 | 19 |
|  | LII-300 (Artium) | 0.95 | 0.02 | 0.99 | 19 |
|  | MSS (AEDC) | 0.91 | 0.01 | 1.00 | 19 |
|  | MSS plus (AVL) | 0.93 | 0.01 | 1.00 | 19 |
|  | MSS plus (AVL) [AVL Cal.] | 0.99 | 0.01 | 1.00 | 19 |
| J85 | CAPS PM_SSA_ (Aerodyne) | 0.80 | 0.01 | 1.00 | 13 |
|  | LII-300 (AEDC) | 0.82 | 0.01 | 1.00 | 13 |
|  | LII-300 (Artium) | 0.87 | 0.01 | 1.00 | 13 |
|  | MSS (AEDC) | 0.84 | 0.01 | 1.00 | 13 |
|  | MSS plus (AVL) | 0.86 | 0.01 | 1.00 | 13 |
|  | MSS plus (AVL) [AVL Cal.] | 0.92 | 0.01 | 1.00 | 13 |

Table S-14. Results of VARIAnT 4 instrument comparison to the EC reference: statistics of linear regressions from Figure 3.

| **Aerosol Source** | **Instrument** | **Slope** | **Slope Standard Error** | **R-Squared** | **Degrees of Freedom** |
| --- | --- | --- | --- | --- | --- |
| CAST | CAPS PM_SSA_ (Aerodyne) | 1.09 | 0.00 | 1.00 | 3 |
|  | LII-300 (HW) | 1.24 | 0.04 | 1.00 | 3 |
|  | MSS (AEDC) | 1.03 | 0.03 | 1.00 | 3 |
|  | MSSplus (HW) | 1.02 | 0.03 | 1.00 | 3 |
| LGT-60 | CAPS PM_SSA_ (Aerodyne) | 1.00 | 0.01 | 1.00 | 11 |
|  | LII-300 (AEDC) | 1.06 | 0.01 | 1.00 | 2 |
|  | LII-300 (Artium) | 0.96 | 0.01 | 1.00 | 5 |
|  | LII-300 (HW) | 0.99 | 0.01 | 1.00 | 11 |
|  | MSS (AEDC) | 0.96 | 0.01 | 1.00 | 11 |
|  | MSSplus (HW) | 0.95 | 0.01 | 1.00 | 11 |
| J85 | CAPS PM_SSA_ (Aerodyne) | 0.92 | 0.01 | 0.99 | 30 |
|  | LII-300 (AEDC) | 0.94 | 0.01 | 1.00 | 13 |
|  | LII-300 (Artium) | 0.73 | 0.03 | 0.95 | 29 |
|  | LII-300 (HW) | 0.70 | 0.01 | 0.99 | 30 |
|  | MSS (AEDC) | 0.87 | 0.01 | 0.99 | 30 |
|  | MSSplus (HW) | 0.86 | 0.01 | 0.99 | 30 |

Table S-15. Results of VARIAnT 4 sizing instrument comparisons: statistics of linear regressions from Figure 5.

| **Panel** | **Instrument** | **Slope** | **Slope Standard Error** | **Slope p-value** | **Slope T-statistic** | **R-Squared** | **Degrees of Freedom** |
| --- | --- | --- | --- | --- | --- | --- | --- |
| Panel A: Total Number | DMS500 (AEDC) | 1.03 | 0.03 | 3.8e-42 | 38.8 | 0.96 | 56 |
|  | EEPS (NVFEL) | 0.67 | 0.04 | 3.1e-23 | 16.1 | 0.81 | 59 |
|  | SMPS 3936 (NVFEL) | 0.89 | 0.01 | 3.8e-68 | 89.6 | 0.99 | 63 |
|  | SMPS 3938 (Aerodyne) | 0.52 | 0.01 | 3.4e-73 | 101.1 | 0.99 | 65 |
|  | SMPS 3938 (Artium) | 1.06 | 0.02 | 3.6e-59 | 67.9 | 0.99 | 61 |
| Panel B: GMD | DMS500 (AEDC) | 0.88 | 0.00 | 1.5e-78 | 176.2 | 1.00 | 56 |
|  | EEPS (NVFEL) | 0.89 | 0.01 | 1.3e-64 | 89.1 | 0.99 | 59 |
|  | SMPS 3936 (NVFEL) | 1.02 | 0.00 | 1e-91 | 212.6 | 1.00 | 63 |
|  | SMPS 3938 (Aerodyne) | 1.26 | 0.01 | 4.4e-84 | 148.8 | 1.00 | 65 |
|  | SMPS 3938 (Artium) | 0.93 | 0.01 | 1.2e-75 | 127.0 | 1.00 | 61 |
| Panel C: Volume | DMS500 (AEDC) | 1.12 | 0.05 | 1.3e-30 | 23.4 | 0.91 | 56 |
|  | EEPS (NVFEL) | 0.54 | 0.03 | 2.4e-24 | 17.0 | 0.83 | 59 |
|  | SMPS 3936 (NVFEL) | 1.09 | 0.15 | 1e-09 | 7.2 | 0.45 | 63 |
|  | SMPS 3938 (Aerodyne) | 1.23 | 0.02 | 1.9e-64 | 73.9 | 0.99 | 65 |
|  | SMPS 3938 (Artium) | 0.81 | 0.01 | 1.4e-78 | 142.0 | 1.00 | 61 |
| Panel D: Volume [15-225nm] | DMS500 (AEDC) | 0.91 | 0.02 | 1.3e-48 | 50.9 | 0.98 | 56 |
|  | EEPS (NVFEL) | 0.50 | 0.03 | 3.6e-23 | 16.0 | 0.81 | 59 |
|  | SMPS 3936 (NVFEL) | 1.09 | 0.15 | 1e-09 | 7.2 | 0.45 | 63 |
|  | SMPS 3938 (Aerodyne) | 1.03 | 0.01 | 9.1e-88 | 169.7 | 1.00 | 65 |
|  | SMPS 3938 (Artium) | 0.81 | 0.01 | 1.1e-78 | 142.4 | 1.00 | 61 |

Table S-16. Results of VARIAnT 3 and 4 mass instrument comparisons: statistics of linear regressions from Figure 6.

| **Panel** | **X-axis** | **Y-axis** | **Campaign** | **Aerosol Source** | **Slope** | **Slope Standard Error** | **R-Squared** | **Degrees of Freedom** | **Ratio of Y over X (Average±Standard Deviation)** |
| --- | --- | --- | --- | --- | --- | --- | --- | --- | --- |
| A | Manual EC | PTFE Filter Mass | VARIAnT 3 | J85 | 1.30 | 0.04 | 0.99 | 12 | 1.65±0.51 |
|  |  |  |  | LGT-60 | 1.46 | 0.06 | 0.97 | 17 | 1.51±0.38 |
|  |  |  | VARIAnT 4 | J85 | 1.41 | 0.04 | 0.98 | 26 | 1.47±0.22 |
|  |  |  |  | LGT-60 | 1.00 | 0.07 | 0.95 | 11 | 1.14±0.40 |
| B | MSS AEDC |  | VARIAnT 3 | J85 | 1.54 | 0.04 | 0.99 | 12 | 2.03±0.72 |
|  |  |  |  | LGT-60 | 1.61 | 0.07 | 0.97 | 17 | 1.66±0.41 |
|  |  |  | VARIAnT 4 | J85 | 1.61 | 0.03 | 0.99 | 26 | 1.67±0.20 |
|  |  |  |  | LGT-60 | 1.04 | 0.07 | 0.95 | 11 | 1.19±0.39 |
| C |  | IPSD Mass | VARIAnT 3 | J85 | 1.25 | 0.02 | 1.00 | 11 | 1.39±0.27 |
|  |  |  | VARIAnT 4 |  | 1.56 | 0.03 | 1.00 | 12 | 1.50±0.14 |

# Comparison of Particle Size Distributions by Various Instruments

As indicated in Table 3 of the main text, an extensive array of sizing instruments was employed during VARIAnT 4. These instruments consisted of up to three TSI 3938 Scanning Mobility Particle Sizers (SMPSs) and one TSI 3936 equipped with various models of TSI Condensation Particle Counters (CPCs), a TSI Model 3090 Engine Exhaust Particle Sizer (EEPS), and a Cambustion Model DMS500 Fast Particle Analyzer (VARIAnT 4). During VARIAnT 4, three of the SMPSs were run at a sample flowrate of 1.5 lpm and sheath flow of 15 lpm and the fourth at a sample flowrate of 0.3 lpm and sheath flow of 3.0 lpm (referred to here as the “low flow” SMPS). The low flow instrument was intended to investigate the presence of larger particles in the engine exhaust as suggested in prior VARIAnT testing. A comparison of the particle sizing instruments in VARIAnT 4 is provided in the Figure S-19 below.

During the two campaigns, a number of specialized instruments were introduced to further characterize the aerosol being sampled. These instruments included a Cambustion Centrifugal Particle Mass Analyzer (CPMA) which classifies particles by their mass to charge ratio from 0.002 -1050 fg/e (corresponding to 7 nm – 1.3 µm diameter for singly charged unit density spheres), a Cambustion Aerodynamic Aerosol Classifier (AAC) which classifies particles by their relaxation time in the 18 ns - 140 ms range (corresponding to 25 nm - 5 µm aerodynamic diameter), and a single particle mass spectrometer (miniSPLAT) (Zelenyuk et al., 2015). The miniSPLAT was used for real-time characterization of individual aerosol particles, making it possible to identify the presence of particles with different properties (compositions, sizes, vacuum aerodynamic diameters (d_va_), masses, shapes, morphologies, and effective densities).

## Example of Size Distributions for Each Instrument for a Single Test Point

As discussed in the main text, there were slight differences in the particle size distribution (PSD) produced by the various online sizing instruments. As an example, Figure S-19 shows the differential number PSDs produced by each instrument during a representative test point (test point T8.3) in VARIAnT 4. Panel (a) in Figure S-19 shows the averaged number distribution for each sizing instrument, panel (b) the number PSDs translated to volume distributions, and panel (c) the total number concentration as a function of time over the test point. As shown, except for the SMPS 3938 low flow (Aerodyne), all of the other analyzers produced slightly different PSDs for the same aerosol. Although GMDs agreed to within ± 2 nm, the modes varied from about 20 to 30 nm for the same aerosol. The size distribution for the low flow (Aerodyne) SMPS instrument is an exception with both the GMD and the mode shifted to significantly larger sizes as shown in panel (a) of Figure S-19 due to undercounting of particles smaller than about 50 nm. Additionally, the range of the low flow SMPS is different from the high flow SMPSs and truncates particles with diameters below about 15 nm~~)~~. As mentioned in the main text, the differences below 50 nm may in part be due to diffusional losses and particle coagulation, but these do not fully reconcile the undercounting differences.

Panel (b) of Figure S-19 shows that the agreement in the volume domain for the low flow (Aerodyne) SMPS is like the other sizing instruments. Finally, panel (c) of Figure S-19 shows that the total concentrations, as measured by each sizing instrument, plotted as a function of time are stable over the length of the test point.

**
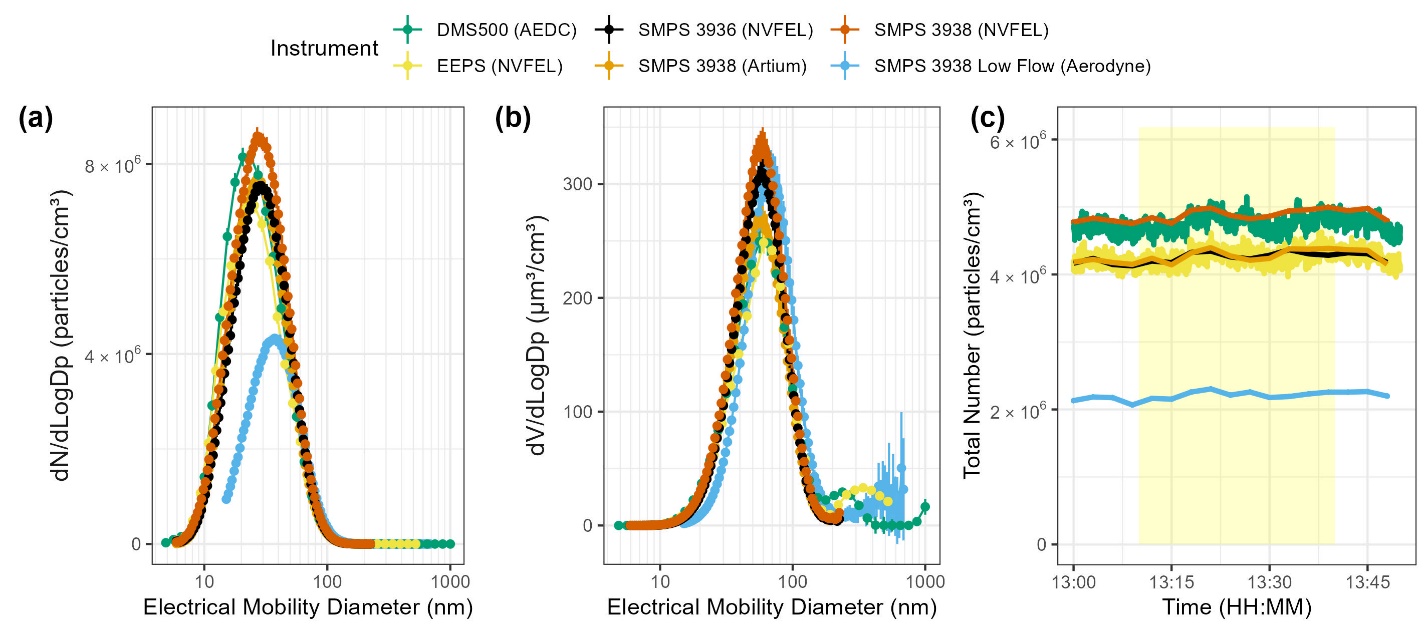
**

Figure S-19. Example of particle size distributions obtained by various sizing instruments during VARIAnT 4 during test point T8.3. Panel (a) is the average size distribution, including error bars, that represent the standard deviation at each particle diameter over the test point. Note the error bars are small and not visible for most points. Panel (b) is the volume distribution translated from the number distribution for each sizing instrument. The error bars for the SMPS 3938 low flow (Aerodyne) are more prominent for particle diameters greater than 500 nm. Panel (c) is the total number concentration as a function of time measured by each sizing instrument from 13:00 to 13:50. The yellow highlight indicates the time of the test window for T8.3, from 13:10 to 13:40.

## EEPS Size Distribution by Test Point

There was considerable variation in the EEPS as mentioned in the main text and highlighted in Figure 5. Figure S-20 shows the ratio of between the EEPS and SMPS 3938 (NVFEL) for the computed geometric standard deviation, geometric mean diameter, and total number concentration by test point during VARIAnT 4. The order of test points is listed in the order which they were run day to day over the course of the test campaign.

During test series T3 on August 14^th^, the total number concentrations of the EEPS relative to NVFEL 3938 SMPS showed a wide range of variability. During test series T5 on August 16^th^, the response of the EEPS total number concentration relative to the NVFEL SMPS decreased over the course of the day.


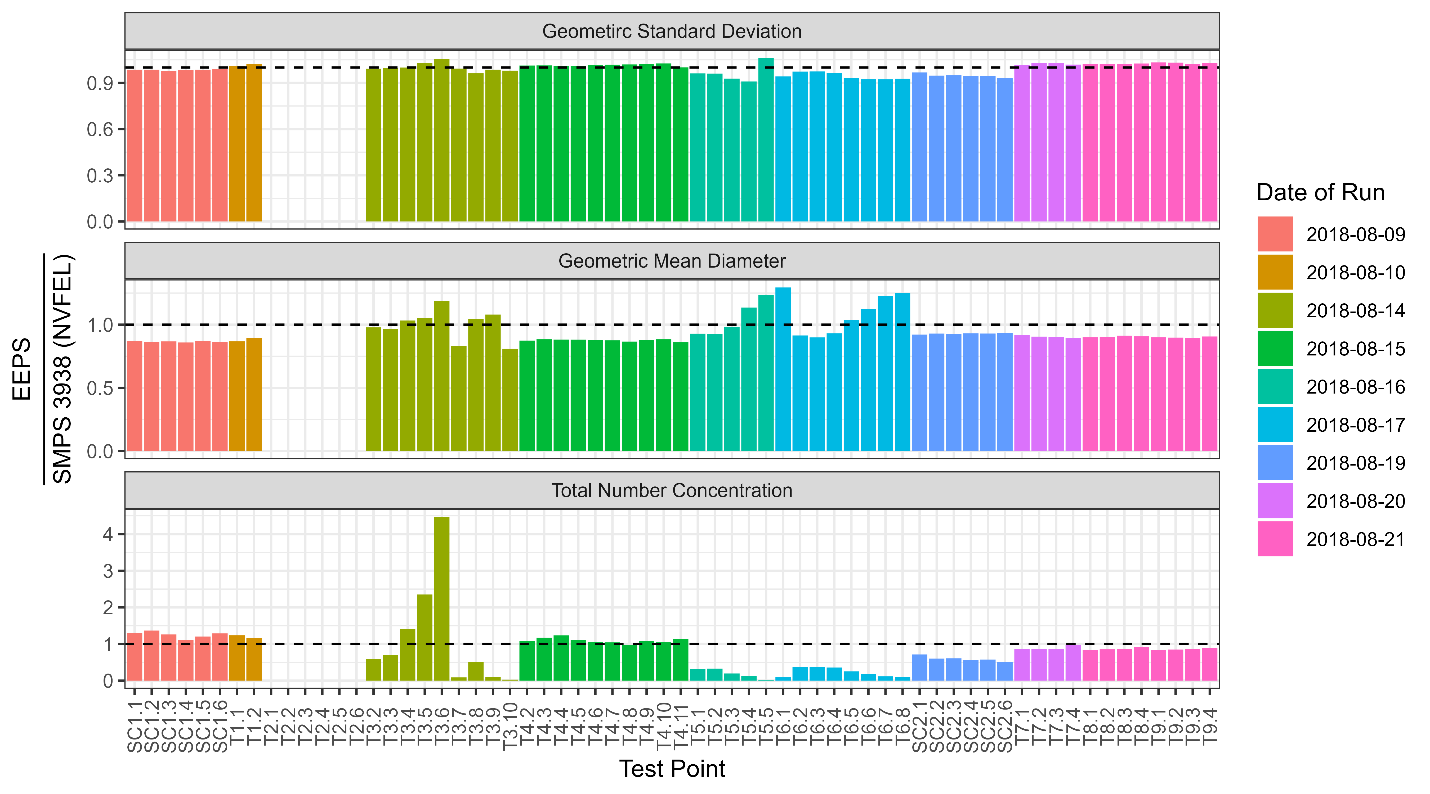


Figure S-20. Ratio between the EEPS and NVFEL 3938 SMPS for geometric standard deviation, geometric mean diameter, and total number concentration by test point during VARIAnT 4. The test points are listed in the order run with the fill color indicating the day of the run.

Similarly on August 17^th^, during test series T6, the EEPS total number concentration was lower than the NVFEL 3938 SMPS and the ratio between the two generally decreased over the course of the day. On the next day during the test series SC2, the EEPS total number concentration relative to the SMPS 3938 (NVFEL) was better than the previous 2 days and relatively stable over the course of the day. Then for the remaining test days of the campaign (test series T7, T8 and T9), the EEPS total number concentration relative to the SMPS 3938 (NVFEL) showed closer to 1:1 agreement.

The data for the 3 parameters (geometric standard deviation, geometric mean diameter and total number concentration) in Figure S-20 was grouped by date of run and fit to a simple linear regression through zero with the parameters from the EEPS on the y-axis and the SMPS 3938 on the x-axis. The slopes from the fits of each parameter for each run day are shown in Figure S-21.


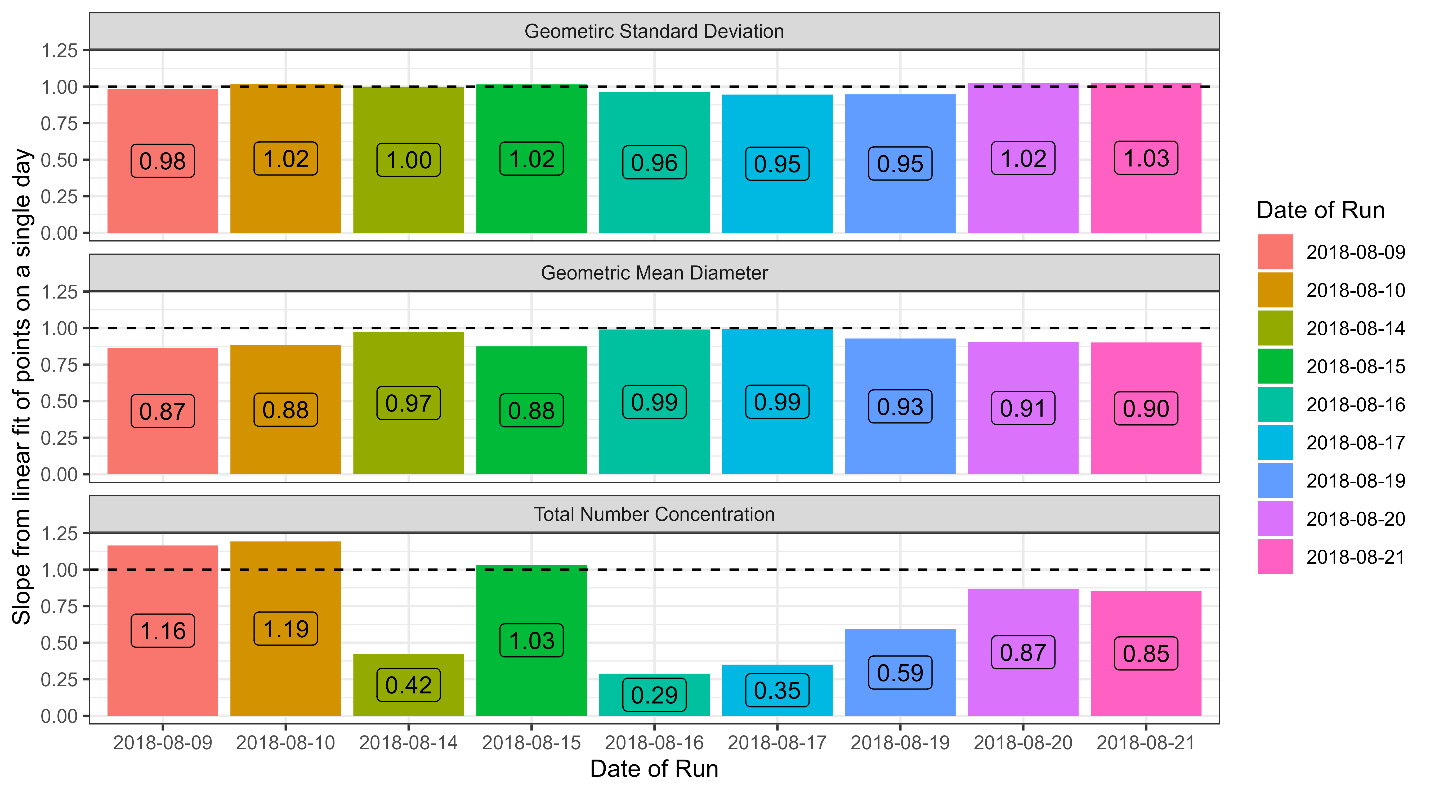


Figure S-21. Slopes from a simple linear regression with a fixed intercept at 0 with the EEPS as the y and NVFEL 3938 SMPS as the x variable for geometric standard deviation, geometric mean diameter, and total number concentration by each test day during VARIAnT 4 where data are available. The values of the slopes are shown on each bar with the fill color indicating the day of the run like that of Figure S-20.

On a daily average basis, the slopes between the EEPS and NVFEL 3938 SMPS for the geometric standard deviation varied by 5% or less in the top panel of Figure S-21. The middle panel of Figure S-21 with the daily slopes for the geometric mean diameter shows that the EEPS measured smaller geometric mean diameters than the NVFEL SMPS by at most 13%. For the total number concentration, the bottom panel of Figure S-21 shows 5 test days where the slopes indicate the difference between the EEPS and NVFEL SMPS was 20% or better on average. For the other 4 days that the EEPS was operational, the EEPS measured total concentrations on average that were 40% to 70% lower than the NVFEL 3938 SMPS.

The reason for the differences in total number concentrations between the EEPS and NVFEL SMPS is unknown. While the total number concentrations do not always agree day to day and test point to test point, the EEPS generally measured a similar shape of size distribution (geometric mean diameter and geometric standard deviation). There is still variability in these size distribution shape parameters, it is not on the same magnitude as the total number concentration.

# CAPS PM_SSA_ Monitor Measurements from VARIAnT 3 and 4

## Black Carbon Mass Measurements

Details on Aerodyne’s CAPS PM_SSA_ monitors are described elsewhere (Onasch et al., 2015). For this work, two different CAPS PM_SSA_ monitors were used to measure the black carbon (BC) mass from 6 different diffusion flame combustion aerosol sources (DFCAS). A 630 nm wavelength CAPS PM_SSA_ monitor was used in VARIAnT 3 and a 780 nm in VARIAnT 4. The CAPS PM_SSA_ measures aerosol optical extinction and scattering coefficients and obtains the aerosol optical absorption coefficient from the difference. BC mass concentrations (µg/m^3^) are derived by dividing the absorption coefficients (Mm^-1^) by a wavelength-dependent MAC (m^2^/g). The wavelength-dependent MAC represents the instrument calibration factor for BC mass measurements. MAC values of 6.6 m^2^/g at 630 nm and 5.25 m^2^/g at 780 nm were obtained from laboratory generated diffusion flame soot and used in the VARIAnT studies. The CAPS PM_SSA_ monitors were calibrated during both VARIAnT studies, following SAE ARP6320 calibration protocols, and the obtained calibration correction factors (0.83 and 1.074, for VARIAnT 3 and 4, respectively) were subsequently applied to all measurements.

As noted in Onasch et al. (2015), the uncertainty in the aerosol absorption measurements is a function of the uncertainty in extinction (~5%) and in the Single Scattering Albedo (SSA), which is a function of particle size. Please refer to Table 1 in Onasch et al. (2015). As most of the measured black carbon particle populations measured in VARIAnT 3 and 4 had particle mobility diameters below 200 nm, we did not correct the CAPS PM_SSA_ monitor scattering and SSA measurements for truncation effects. These truncation effects are estimated to be negligible at wavelengths of 630 nm and 780 nm (Refer to Figure 6 and Appendix truncation model in Onasch et al., 2015), in agreement with results published in Modini et al. (2021).

## Single Scattering Albedo Measurements

Single Scattering Albedo measurements from the CAPS PM_SSA_ have been previously described (Kinsey et al., 2021).^[[1]](#footnote-2)^ The CAPS PM_SSA_ Monitors in VARIAnT 3 used a MAC of 6.6 m^2^/g (at a wavelength of 630 nm) and in VARIAnT 4 a MAC of 5.25 m^2^/g (at a wavelength of 780 nm).

Figure S-22 shows CAPS PM_SSA_ SSA measured in VARIAnT 3 and 4 for J85 jet engine operation with Jet-A fuel and fuel blends of 30%, 45% and 70% Camelina with Jet-A. The SSA values increased with decreasing engine power similar to the trend displayed in VARIAnT 2 (Kinsey et al., 2021). As noted in the main text, the measured nvPM geometric mean diameter increased with increasing PLA.

In most cases, measured SSA values were above 0.1 and increased with decreasing PLA. This could be caused by changing particle refractive index/chemical composition (i.e., less absorbing material and more scattering material), changing particle shape (compact shapes scatter more), and particle size (larger particles scatter more). More discussion of compact particles and large secondary mode particles will be provided in a subsequent publication.


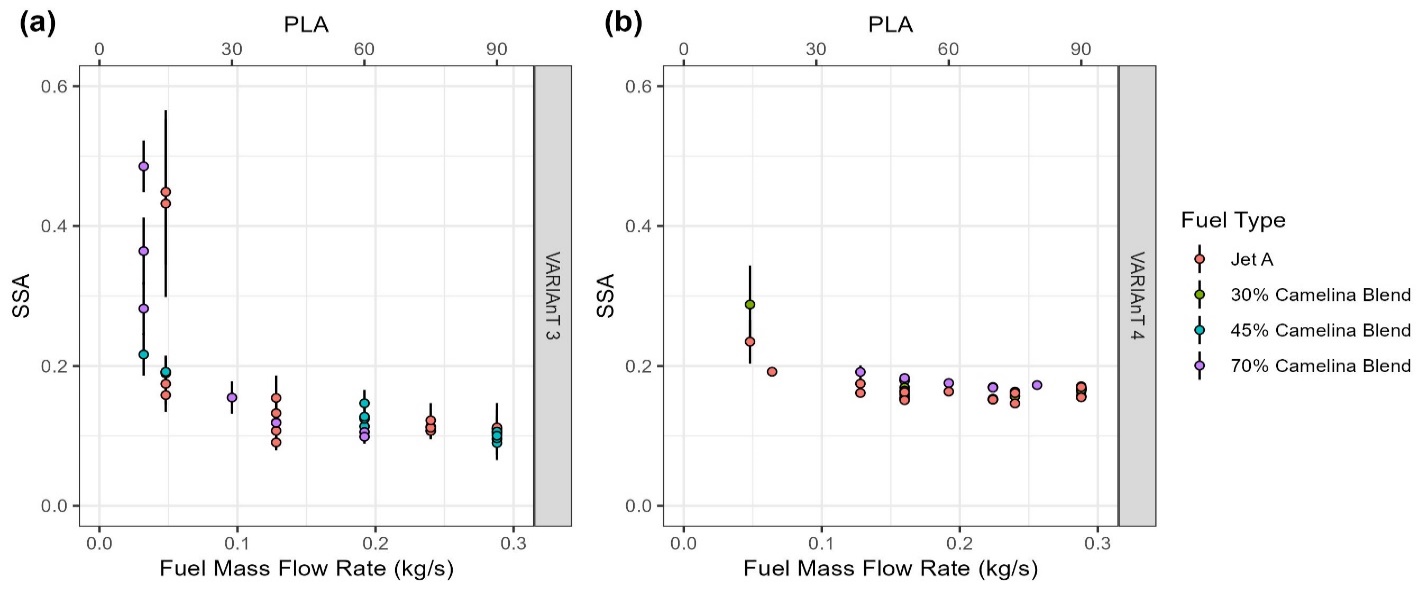


Figure S-22. CAPS PM_SSA_ SSA measurements (scattering to extinction ratio) during VARIAnT 3 in panel (a) and VARIAnT 4 in panel (b) as a function of Fuel Mass Flow Rate. Data are colored by the fuel type. Error bars represent the standard deviation of the measurement for each test point. Additionally, the engine power lever angle (PLA) in degrees for J-85 are shown on the top of each plot for the corresponding fuel flow. Table S-4 for nominal fuel flow for the various PLA settings.

# LII fluence Sweeps from VARIAnT 4

Several test points during VARIAnT 4 include pretest-point fluence sweeps. This was to examine possible effects of fluence on LII operation (see Yuan et al., 2022 for a discussion of fluence sweeps). A ratio of the mass instrument concentration to reference EC mass concentration was calculated for each J85 test point in VARIAnT 4. The J85 was the only source that had data points with and without fluence sweeps before a test point. A histogram was then constructed from these ratios for each mass instrument which is shown in Figure S-23 with a single count representing one computed ratio. The color in the histograms indicates if a fluence sweep was done before the test point or if the instrument was not an LII-300.^[[2]](#footnote-3)^

The number of points with a fluence sweep before the point was limited with only the AEDC and HW LII-300 being capable of doing fluence sweeps. Data are only shown for the J85 in VARIAnT 4 for those test points where filters were collected for reference EC measurements. Figure S-23 demonstrates that test points with a fluence sweep immediately before the point showed a similar response to the test points that did not have a fluence sweep conducted prior to the test point.


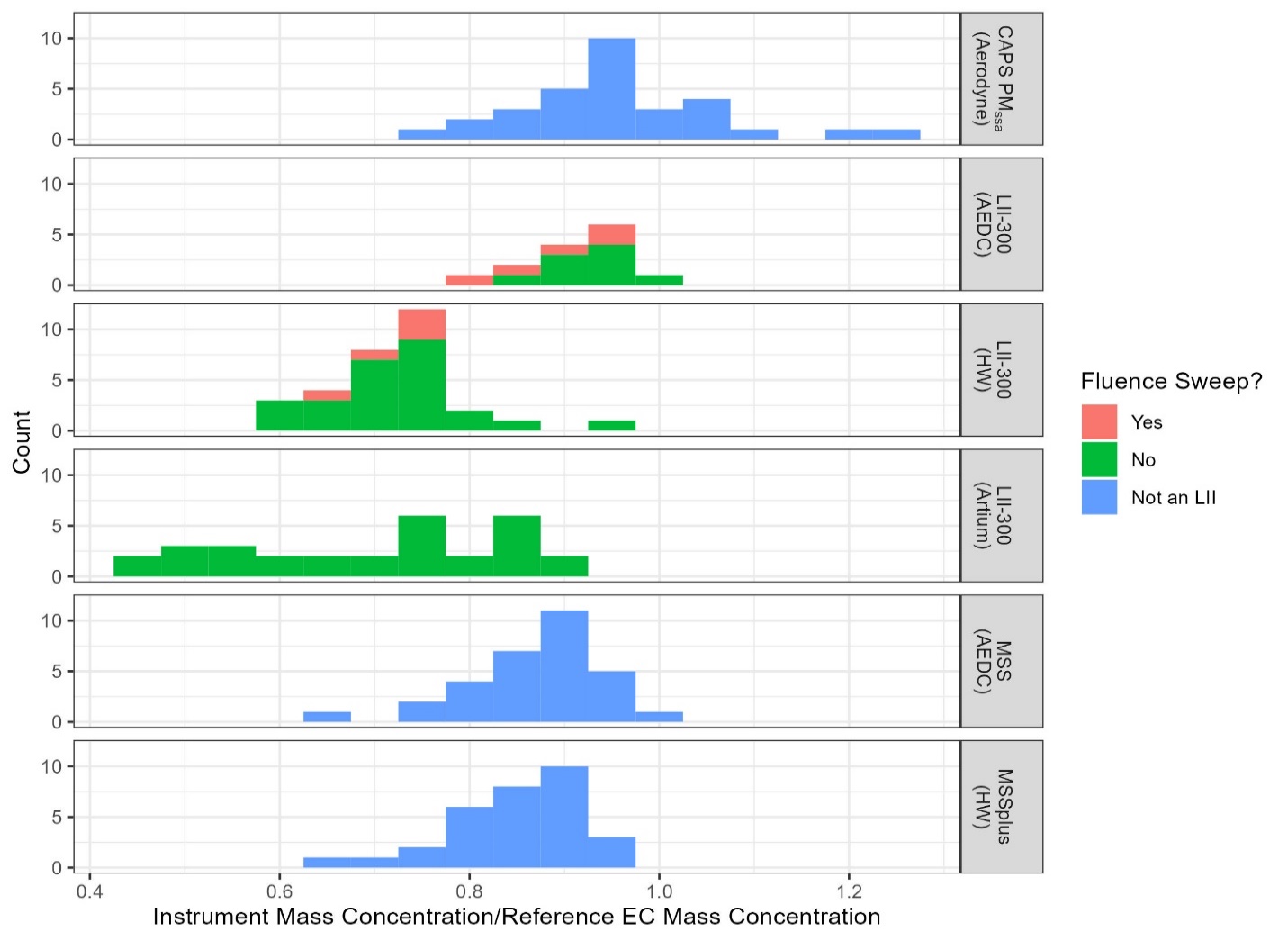
**Figure S-23.** Histograms of the ratio each real-time mass instrument concentration to Reference EC mass concentration for J85 test points during VARIAnT 4. Each panel shows the histogram for individual instruments. Data are colored by whether a fluence sweep was performed or if the instrument is not an LII-300.

# Student’s t-test results of LII instrument response associated with particle electrical mobility geometric mean diameters

Figure 4 of the main text showed that although there was significant scatter, the relative response of the LII appeared to decrease with decreasing particle size. We tested the hypothesis that the instrument relative differences measured are significantly different above and below a given diameter. Table S-17 lists the Student’s t-test probabilities of the MSS plus and the AEDC MSS instrument relative differences paired with the instrument relative differences of the other BC mass instruments for 4 different ranges of electrical mobility geometric mean diameters. Of note are the Student’s t-test probabilities for the AEDC LII and the loaner LII above and below 36 nm. These data are indicative of the trend that we are reporting in the paper. That is, the relative differences below about 35 nm deviate from the relative differences of the MSS instruments. For geometric diameters above 35 nm the relative differences of the instruments are more comparable with one another. The other instruments do not show these very distinct differences associated with the particle electrical mobility geometric mean diameters.

Table S-17. Results from t-tests of instrument relative differences (see Figure 4b in the main text) paired with the AEDC MSS and MSS plus relative responses from the VARIAnT3 test campaign.

| Data ranges | 1 - (MSS_plus_/MSS_AEDC_) | 1- (LII_AEDC_/MSS_AEDC_) | 1 – (LII _loaner_/MSS_AEDC_) | 1 - (MSS_plus[AVL Cal.]_/MSS_AEDC_) | 1 – (CAPS/MSS_AEDC_) |
| --- | --- | --- | --- | --- | --- |
|  | Student’s t-test probabilities using MSS_plus_ & MSS_AEDC_ relative differences as the reference data set | | | | |
| geometric means <30nm | 1 | 1.52×10^-10^ | 4.6×10^-07^ | 1.64×10^-14^ | 0.0042 |
| geometric means <36nm | 1 | 1.41×10^-08^ | 5.4×10^-06^ | 1.00×10^-26^ | 0.00025 |
| geometric means >=36nm | 1 | 0.42 | 0.57 | 1.27×10^-9^ | 0.013 |
| geometric means >=40nm | 1 | 0.31 | 0.56 | 1.31×10^-7^ | 0.027 |

# Equations for calculation of IPSD and SMPS volume

Number distributions from sizing instruments were converted to volume distributions using the following expression:

$$\frac{dV}{dLogDp}=\frac{\pi}{6} {Dp}^{3}\frac{dN}{dLogDp}$$

Where:

$\frac{dN}{dLogDp}$ is the measured number for a given bin width, in units of particles/cm³

Dp is the midpoint of the particle bin, in micrometers

Using the above units will result in units of µm³/cm³ for the $\frac{dV}{dLogDp}$ term.

The average volume for a single test point was computed from the volume from a size distribution using the following expression:

$$V=\int_{l}^{u} \frac{dV}{dLogDp}dDp=\frac{\pi}{6}\int_{l}^{u} {Dp}^{3}\frac{dN}{dLogDp}dDp$$

IPSD mass was calculated from the number distribution using the following expression:

$$M_{IPSD}=\frac{\pi}{6}\int_{l}^{u} \rho{Dp}^{3}\frac{dN}{dLogDp}dDp$$

Where:

$\frac{dN}{dLogDp}$ is the measured number for a given bin width, in units of particles/cm³

Dp is the midpoint of the particle bin, in micrometers

$\rho$ is the effective density, in g/cm³

*l* is the smallest particle diameter of the size distribution

*u* is the smallest particle diameter of the size distribution

Using the above units will result in units of µg³/m³ for the $M_{IPSD}$ term.

A few more details on the method of determining the IPSD from the SMPS and CPMA measurements follow. A density distribution as a function of particle mobility diameter was determined using the instruments configured with the DMA upstream of the CPMA (DMA-CPMA-CPC configuration). In this configuration, a particle diameter, d_i_, was selected with the DMA and then a mass scan was performed to measure the mass distribution by selecting a range of CPMA mass to charge ratio (and assuming the charge of each particle traversing through CPMA was 1e = 1.602 × 10^-19^ Coulomb). At a given DMA selected particle mobility diameter, d_i_, the density is a function of the normalized particle mass fractions, m_total_·f(d_i_, m_j_), and the particle volume, πd_i_^3^/6. For the IPSD mass calculations in this work the density was defined at the DMA selected diameter, d_i_, and the mode of the mass distribution, m_j_, (determined from a lognormal fit of the CPMA measured mass distribution),

$$\rho(d_{i})=m_{total}\cdot\left\{ f\left( d_{i},m_{1} \right)+f\left( d_{i},m_{2} \right)+\ldots+f\left( d_{i},m_{N_{m}} \right) \right\}/[\frac{\pi d_{i}^{3}}{6}])\equiv m_{i}/[\frac{\pi d_{i}^{3}}{6}]$$

The integrated particle size distribution (IPSD) mass was then calculated using the SMPS measured size distributions and the size dependent density,

$$M_{IPSD}=\sum_{d_{i}} \frac{\pi\cdot d_{i}^{3}}{6}\cdot n\left( d_{i} \right)\cdot\rho(d_{i})\cdot\Delta\log(d_{i})$$

where the n(d_i_) are the number concentrations from the size distribution measurements. Using the units of micrometers for $d_{i}$, g/cm³ for $\rho$, particles/cm³ for $n\left( d_{i} \right)$ results in a mass concentration of µg/m³ for $M_{IPSD}$.


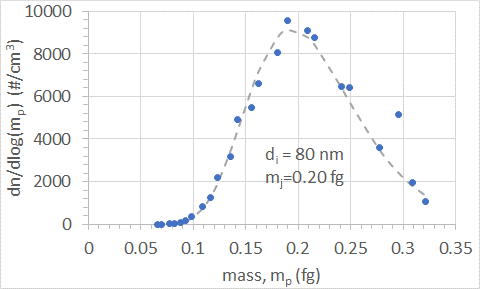


**Figure S-24.** Example of a CPMA mass scan for a DMA selected diameter of d_i_=80 nm. The mass mode, m_j_=0.20 fg for this scan was determined from a lognormal fit of the mass distribution depicted with the dashed grey line.

Note : The relationship between elemental carbon, organic carbon, and total carbon in PM emissions from turbine engines is well documented in the literature (Birch et al., 1996; Bond et al., 2006; Kinsey et al., 2010; Kinsey, 2011; Kinsey et al., 2012; Drozd, et al., 2012; Abegglen et al., 2016; Klingshirn, et al., 2019; Smith et al., 2022)

# Example of the effect of catalytic stripper on secondary particle mode

Figure S-25 shows an example of the presence of the secondary particle mode in the volume domain with and without a catalytic stripper as measured with the SMPS 3938 (Aerodyne) in low flow mode. Also shown is the number distribution for each test point. The test points with a catalytic stripper (T4.5) and without a catalytic stripper (T4.6) were run in succession on August 15, 2018. Each test point was approximately 1 hour.


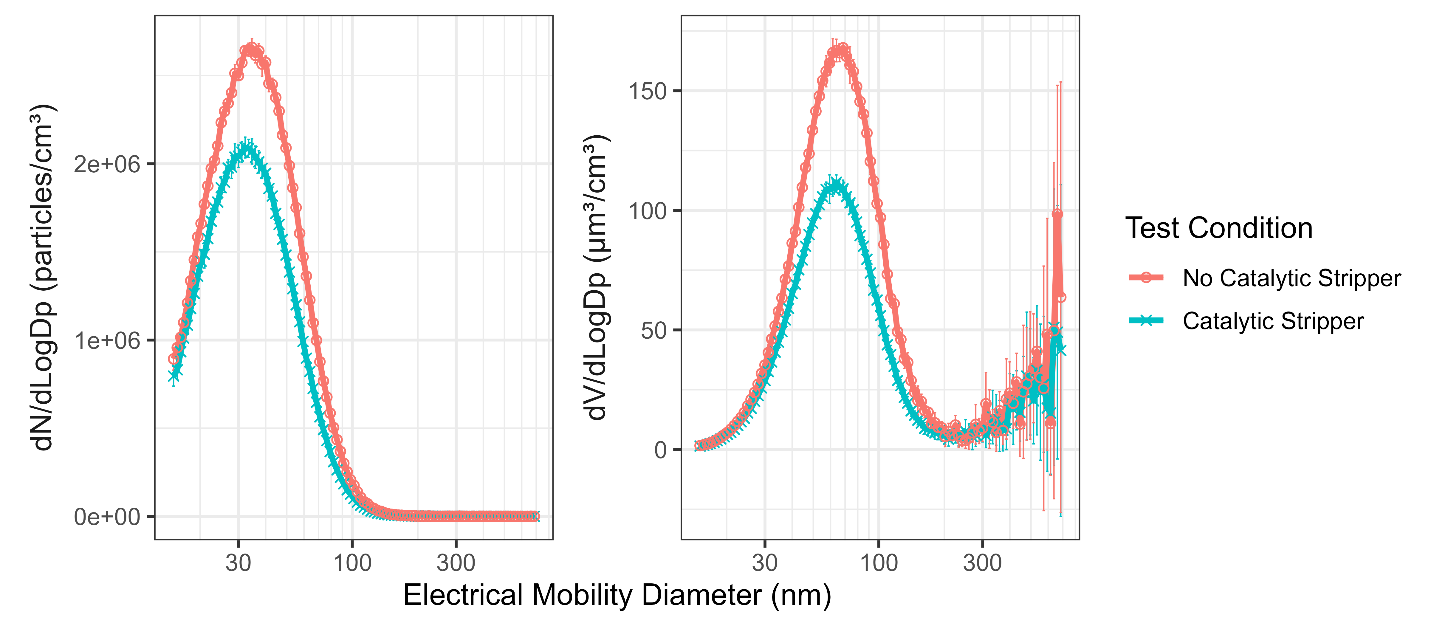


**Figure S-25.** Example of SMPS size distributions in the number and volume domain with and without a catalytic stripper for the J85 burning Jet-A at PLA 70. The points are the average at each particle bin over each test point. The error bars represent 1 standard deviation for each particle bin over the time of the test point. The solid line represents the average distribution over each test point.

# References

Birch ME, and Cary RA 1996. Elemental carbon-based method for monitoring occupational exposures to particulate diesel exhaust. Aerosol Sci. Technol 25:221–41. doi: 10.1080/02786829608965393.

Bond TC, and Bergstrom RW 2006. Light absorption by carbonaceous particles: An investigative review. Aerosol Sci. Technol 40:27–67. doi: 10.1080/027868205000421521.

Kinsey JS, Dong Y, Williams DC, and Logan R 2010. Physical characterization of the fine particle emissions from commercial aircraft engines during the aircraft particle emissions eXperiment (APEX) 1–3. Atmos. Environ 44:2147–56. doi: 10.1016/j.atmosenv.2010.02.010.

Kinsey JS, Hays MD, Dong Y, Williams DC, and Logan R 2011. Chemical characterization of the fine particle emissions from commercial aircraft engines during the aircraft particle emissions eXperiment (APEX) 1 to 3. Environ. Sci. Technol 45:3415–21. doi: 10.1021/es103880d.

Greg T. Drozd, Marissa A. Miracolo, Albert A. Presto, Eric M. Lipsky, Daniel D. Riemer, Edwin Corporan, and Allen, Robinson 2012. Particulate Matter and Organic Vapor Emissions from a Helicopter Engine Operating on Petroleum and Fischer–Tropsch Fuels, Energy Fuels, 26, 8, 4756–4766, <https://doi.org/10.1021/ef300651t>

Kinsey JS, Timko MT, Herndon SC, Wood EC, Yu Z-H, Miake-Lye RC, Lobo P, Whitefield P, Hagen D, Wey C, et al. 2012. Determination of the emissions from an aircraft auxiliary power unit (APU) during the alternative aviation fuels experiment (AAFEX). 2012. J. Air & Waste Manage. Assoc 62:420–30.

Onasch, Timothy B., Paola Massoli, Paul L. Kebabian, Frank B. Hills, Fred W. Bacon, and Andrew Freedman 2015. “Single Scattering Albedo Monitor for Airborne Particulates.” Aerosol Science and Technology 49, no. 4 (April 3, 2015): 267–79. <https://doi.org/10.1080/02786826.2015.1022248>

Abegglen M, Brem BT, Ellenrieder M, Durdina L, Rindlisbacher T, Wang J, Lohmann U, and Sierau B 2016. Chemical characterization of freshly emitted particulate matter from aircraft exhaust using a single particle mass spectrometer. Atmos. Environ 134:181–97. doi: 10.1016/j.atmosenv.2016.03.051.

Christopher D. Klingshirn, Zachary J. West, Matthew J. DeWitt, Ashil Higgins, John Graham & Edwin Corporan 2019. Quantification of elemental and total carbon in combustion particulate matter using thermal-oxidative analysis, Journal of the Air & Waste Management Association, 69:8, 1003-1013, DOI: 10.1080/10962247.2019.1630025

Kinsey, J. S., Giannelli, R., Howard, R., Hoffman, B., Frazee, R., Aldridge, M., Leggett, C., et al. 2021. “Assessment of a Regulatory Measurement System for the Determination of the Non-Volatile Particulate Matter Emissions from Commercial Aircraft Engines.” Journal of Aerosol Science 154 (May): 105734. <https://doi.org/10.1016/j.jaerosci.2020.105734>

Modini, Rob L., Joel C. Corbin, Benjamin T. Brem, Martin Irwin, Michele Bertò, Rosaria E. Pileci, Prodromos Fetfatzis, et al. 2021. “Detailed Characterization of the CAPS Single-Scattering Albedo Monitor (CAPS PMssa) as a Field-Deployable Instrument for Measuring Aerosol Light Absorption with the Extinction-Minus-Scattering Method.” Atmospheric Measurement Techniques 14, no. 2 (February 3, 2021): 819–51. <https://doi.org/10.5194/amt-14-819-2021>

Liam D. Smith, James Allan, Hugh Coe, Ernesto Reyes-Villegas, Mark P. Johnson, Andrew Crayford, Eliot Durand, Paul I. Williams, 2022. Examining chemical composition of gas turbine-emitted organic aerosol using positive matrix factorisation (PMF), Journal of Aerosol Science, Volume 159, January 2022, 105869

Yuan, Ruoyang, et al. "Measurement of black carbon emissions from multiple engine and source types using laser-induced incandescence: sensitivity to laser fluence." *Atmospheric Measurement Techniques* 15.2 (2022): 241-259.

Zelenyuk, A., D. Imre, J. Wilson, Z. Zhang, J. Wang, K. Mueller (2015). Airborne single particle mass spectrometers (SPLAT II & miniSPLAT) and new software for data visualization and analysis in a geo-spatial context, *J. Am. Soc. Mass Spectrom*., 26:257Y270.

1. [↑](#footnote-ref-2)
2. Instruments not capable of fluence sweeps were included as a comparison to the LII-300s that were able to perform fluence sweeps. [↑](#footnote-ref-3)
